# The *GRAS* gene family in pine: transcript expression patterns associated with the maturation-related decline of competence to form adventitious roots

**DOI:** 10.1186/s12870-014-0354-8

**Published:** 2014-12-30

**Authors:** Dolores Abarca, Alberto Pizarro, Inmaculada Hernández, Conchi Sánchez, Silvia P Solana, Alicia del Amo, Elena Carneros, Carmen Díaz-Sala

**Affiliations:** Department of Life Sciences, University of Alcalá, Ctra. de Barcelona Km 33.600, 28805 Alcalá de Henares, Madrid Spain; Department of Plant Physiology, Instituto de Investigaciones Agrobiológicas de Galicia (CSIC), Apartado 122, 15080 Santiago de Compostela, Spain

**Keywords:** Age, Cell fate, Conifer, Developmental plasticity, Intrinsically disordered proteins, Pluripotency, Root meristem, Vegetative propagation

## Abstract

**Background:**

Adventitious rooting is an organogenic process by which roots are induced from differentiated cells other than those specified to develop roots. In forest tree species, age and maturation are barriers to adventitious root formation by stem cuttings. The mechanisms behind the respecification of fully differentiated progenitor cells, which underlies adventitious root formation, are unknown.

**Results:**

Here, the *GRAS* gene family in pine is characterized and the expression of a subset of these genes during adventitious rooting is reported. Comparative analyses of protein structures showed that pine GRAS members are conserved compared with their relatives in angiosperms. Relatively high *GRAS* mRNA levels were measured in non-differentiated proliferating embryogenic cultures and during embryo development. The mRNA levels of putative GRAS family transcription factors, including *Pinus radiata*’s SCARECROW (SCR), *PrSCR*, and SCARECROW-LIKE (SCL) 6, *PrSCL6*, were significantly reduced or non-existent in adult tissues that no longer had the capacity to form adventitious roots, but were maintained or induced after the reprogramming of adult cells in rooting-competent tissues. A subset of genes, *SHORT-ROOT* (*PrSHR*), *PrSCL1*, *PrSCL2*, *PrSCL10* and *PrSCL12*, was also expressed in an auxin-, age- or developmental-dependent manner during adventitious root formation.

**Conclusions:**

The GRAS family of pine has been characterized by analyzing protein structures, phylogenetic relationships, conserved motifs and gene expression patterns. Individual genes within each group have acquired different and specialized functions, some of which could be related to the competence and reprogramming of adult cells to form adventitious roots.

**Electronic supplementary material:**

The online version of this article (doi:10.1186/s12870-014-0354-8) contains supplementary material, which is available to authorized users.

## Background

**Figure 1:**
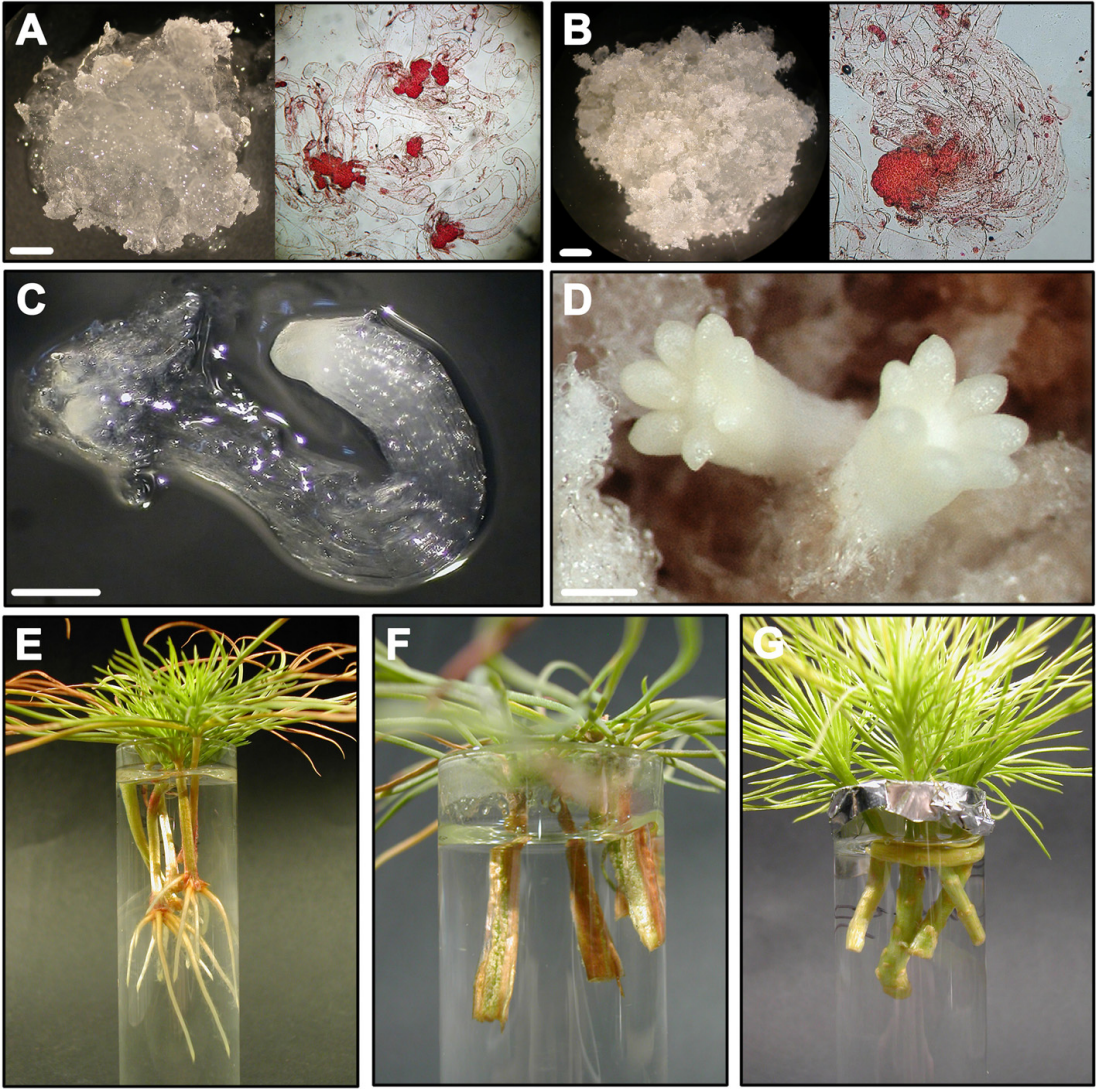
**Experimental system used for analysis.** **A**, **B)** Embryogenic masses of *Pinus radiata* after 7 (P7) and 14 (P14) days of proliferation. Embryogenic tissue (in red) was stained with 1% acetocarmine. Bar: 2 mm. **C)** Early-maturation embryo at polarization stage (M1). Bar: 0.5 mm. **D)** Late-maturation embryo at tissue differentiation stage (M3). Bar: 0.8 mm. **E)** Hypocotyls from 21-day-old seedlings treated with 10 μM indole-3-butyric acid (IBA) after 28 days of culture. **F, G)** Hypocotyls **(F)** and epicotyls **(G)** from 90-day-old seedlings treated with 10 μM IBA.

Adventitious root formation is an organogenic process induced in stem cuttings, or in intact plants, by which roots are induced from differentiated cells other than those specified to develop roots. In forest tree species, a decline in the capacity to regenerate shoots, roots or embryos from somatic differentiated cells in an ectopic location is associated with tree age and maturation [1]. Maturation is an age-related developmental process described in vascular plants that affects morphology, growth rate and other physiological and developmental traits [2-6]. Four phases of maturation have been recognized: (1) the embryonic phase, (2) the post-embryonic juvenile vegetative phase, (3) the adult vegetative phase, and (4) the adult reproductive phase [1,7]. The decline in the ability to form adventitious roots from stem cuttings is a maturational trait that limits the successful vegetative propagation of adult trees. Regeneration efficiency is much higher in tissues at earlier stages of development. However, the mechanisms behind the respecification of fully differentiated progenitor cells to induce a root meristem in an ectopic location, especially in relation to the cell’s developmental age, are unknown [8-14]. Experimental systems based on the differential rooting capacities in response to auxin in hypocotyl and epicotyl cuttings from young seedlings of pine have revealed clues to the underlying mechanisms [10,11,15-18]. Hypocotyl cuttings from 21-day-old seedlings rapidly form adventitious roots, while hypocotyl or epicotyl cuttings from 90-day-old *Pinus radiata* seedlings do not root or root poorly (Figures 1 E, F, G). A continuous ring of mature and active cambium, and a complete ring of secondary xylem were developed in non-competent hypocotyls and epicotyls from 90-day-old seedlings, with interruptions at the primary leaf-axillary bud traces in epicotyls. However, while the cambium was beginning to form, it was not yet differentiated or active in competent hypocotyls from 21-day-old seedlings [10,17,19,20]. Cells competent to form adventitious roots are confined to the cambial region, which is mostly located centrifugal to the resin canal at the xylem poles of the hypocotyl from 21-day-old seedlings. These cells exhibit rapid division and the re-orientation of divisional planes to directly organize a root meristem in response to exogenous auxin, without becoming a developmentally non-identified callus cell. Hypocotyl or epicotyl cambial cells from 90-day-old seedlings respond to the presence of exogenous auxin by dividing, but the re-orientation of the divisional planes needed for the direct organization of a root meristem does not occur or occurs infrequently. Therefore, auxin-induced adventitious root meristem organization appears to occur independently of cell reorganization and division, and the capacity to re-enter the cell division cycle alone [21,22] is not sufficient to reset the previous cellular state in non-competent cells [10,15,18]. De Almeida et al. [23] described the procambial cells as niches of pluripotent and totipotent stem-like cells for organogenesis and somatic embryogenesis, and Hutchison et al. [11] proposed that the maturation-related decline of adventitious root formation could result from the suppression of gene expression levels that are needed for adult cells to re-enter the embryonic root formation pathway. The mechanisms that enable a somatic differentiated cell to become a pluripotent or totipotent cell, which can develop a root, shoot, or embryo, or repair damaged tissues, are unknown.

While auxins do not seem to be the limiting factor at the rooting site in the ability to form adventitious roots at the mature stage [10,24-26], the capacity to recruit root meristem or embryonic programs, and the effects of auxin and cytokinin signaling pathways on the regulation of genes involved in the organization of stem cell niches seem to be key factors in the *de novo* regeneration of several plant species [27-36]. The capacity of cells to generate polar changes in the local distribution of auxin can also influence cell fate [37]; alternatively, transcriptional regulatory networks can function as developmental signals underlying changes in a cell’s fate [33,34,38,39]. The establishment of an embryonic root meristem involves members of the GRAS family of putative transcription factors, which includes SCARECROW (SCR), SCARECROW-LIKE (SCL) and SHORT-ROOT (SHR) proteins. These genes are also involved in the radial patterning of roots, hypocotyls and aerial organs. Their expression is associated with auxin distribution in the root apical meristem [40-45]. A *P. radiata SCARECROW-LIKE* (*PrSCL1*) gene and a *Castanea sativa SCARECROW-LIKE* (*CsSCL1*) gene, which are expressed in roots and root primordia, and are induced in rooting-competent cells at the earliest stages of adventitious root formation in the presence of exogenous auxin, have been previously reported [16,17,46]. Additionally, Solé et al. [17] described a *P. radiata SHORT-ROOT* (*PrSHR*) gene that is also expressed in roots and root primordia, and is induced in rooting-competent cells at the earliest stages of adventitious root formation in the absence of exogenous auxin. These authors concluded that these genes and, perhaps, a GRAS cascade of transcription factors play roles during the earliest stages of adventitious root induction via auxin-dependent and auxin-independent pathways [18].

To investigate if GRAS transcription factors could be associated with the maturation-related decline in adventitious rooting, the GRAS family in pine was characterized. Additionally, the transcript profiles of 13 *GRAS* genes in rooting-competent and rooting-non-competent cuttings in response to auxin were compared at the earliest stages of adventitious root formation, the cell reorganization state, prior to the onset of cell divisions leading to the formation of an adventitious root meristem. The expression analysis was also performed until after the initiation of the rapid cell divisions that organize the root meristem. Auxin distribution was analyzed over the same time course. We also examined the transcript profiles of *GRAS* genes during somatic embryogenesis [47], at the stages of initial-cell formation, embryo polarization and embryo differentiation (Figures 1 A, B, C, D).

## Results

### The pine *GRAS* gene family: *in silico* identification of *GRAS* genes, motif prediction and phylogenetic analysis of GRAS proteins

To further our previous work on pine *GRAS* genes and their roles in the maturation-related decline of adventitious root formation [16,17,46], an *in silico* search was conducted to identify new members of the pine GRAS family. An initial BLAST search of *Pinus* and *Picea* sequences in the Genbank database [48], using a conserved sequence of the GRAS motif, led to the identification of 31 EST sequences that were classified into 13 groups representing putative unigene sequences. *P. radiata* sequences obtained in our lab were used to design primers for expression analyses (see below).

After a second round of searching using the Europine database [49], a total of 90 ESTs and genomic sequences from *Picea glauca*, *Picea sitchensis*, *Pinus albaucalis*, *Pinus ayacahuite*, *Pinus banksiana*, *Pinus bungeana*, *Pinus cembra*, *Pinus contorta*, *Pinus densiflora*, *Pinus flexilis*, *Pinus gerardiana*, *Pinus korainensis*, *Pinus lambertiana*, *Pinus monticola*, *Pinus morrisonicola*, *Pinus pinaster*, *Pinus pinea*, *Pinus radiate*, *Pinus strobiformis*, *Pinus sibirica*, *Pinus squamata*, *Pinus sylvestris*, *Pinus taeda*, *Pinus thumbergii*, and *Pinus wallichiana* were obtained. Additionally, three full-length cDNAs from *P. radiata* [16,17] and five 3′end cDNAs from *P. radiata, P. pinea* and *P. pinaster* that were available in our databases were included, for a total of 98 cDNA sequences. The *in silico* comparison of these sequences resulted in the identification of 21 unique members of the *GRAS* gene family in pine.

After the release of the *Picea abies* and *P. taeda* genomic sequences, a third round of searching using the Congenie and Dendrome databases [50,51] was performed. A total of 36 *P. abies* and 65 *P. taeda* genes models were classified and, together with the previously identified ones, led to the identification of 32 unique members of the pine *GRAS* gene family (Additional file 1). In addition to the *SCR* and *SHR* genes, the predicted genes were named following the nomenclature of our previous work [16], *SCL1* to *SCL30* (Additional file 1).

For 25 of the 32 *GRAS* genes, at least one predicted gene was identified in both pine and spruce (Additional file 1). Seven additional predicted genes were found in *P. taeda* that had no putative orthologs in *P. abies* or other pine species (Additional file 1). Pairwise comparisons among the predicted amino acid sequences of the 25 members for which more than one complete sequence was found revealed a high degree of conservation. Sequence identities ranged from 89.7% to 99.5% between pine sequences and from 84.7% to 97.2% between pine and spruce sequences, except for sequences related to AtSCL26 (see below), which showed a higher divergence between pine and spruce, ranging from 72.0 to 88.4%.

**Figure 2:**
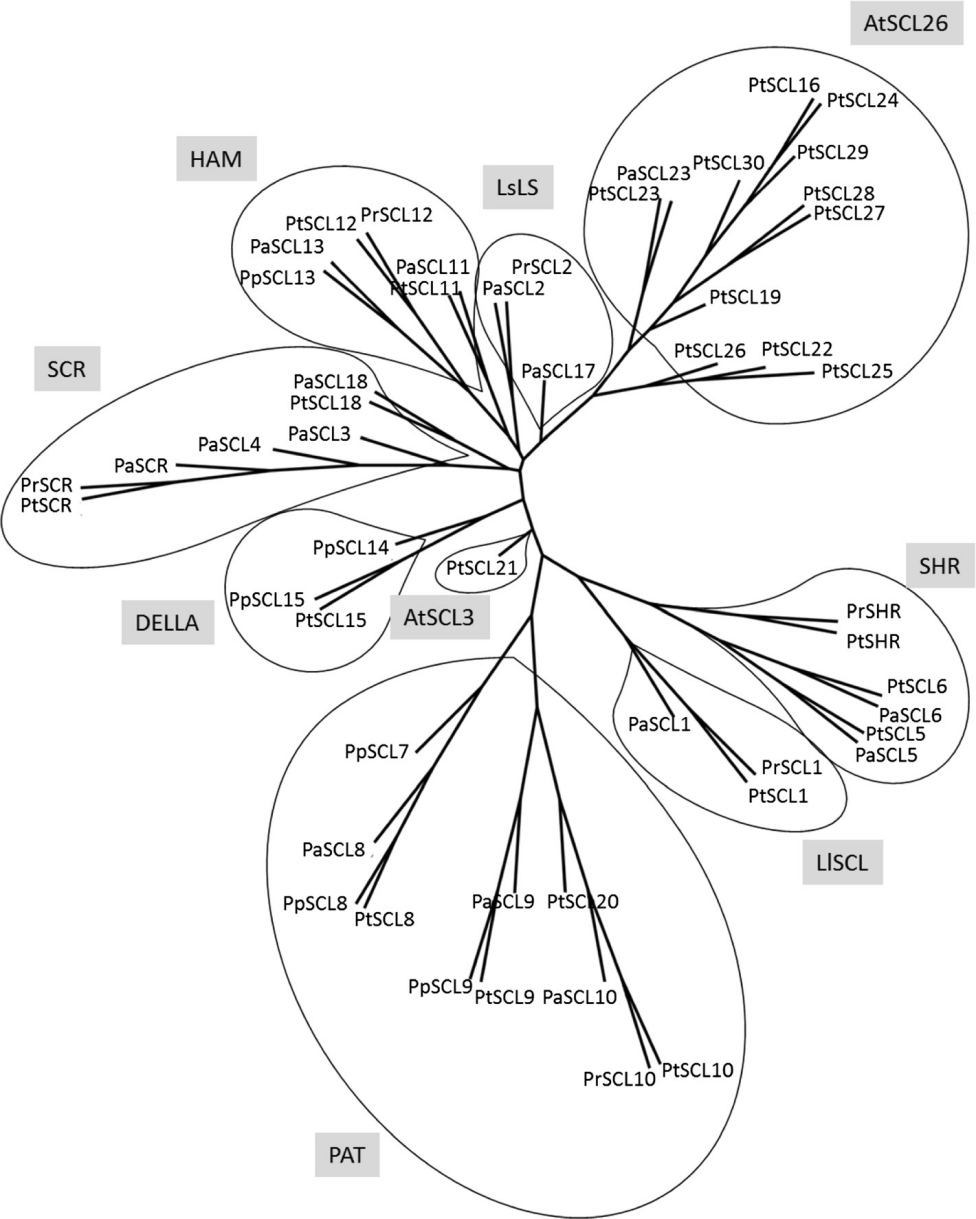
**Phylogenetic tree of GRAS proteins SCARECROW-LIKE (SCL), SCARECROW (SCR), and SHORT-ROOT (SHR) from conifer species.** Accession no. or gene references in parentheses. *Picea abies* SCR (MA_1793p0010), *P. abies* SCL1 (MA_45656p0030), *P. abies* SCL2 (MA_10435790p0010), *P. abies* SCL3 (MA_140003p0010), *P. abies* SCL4 (MA_18234p0010), *P. abies* SCL5 (MA_73870p0010), *P. abies* SCL6 (MA_94287p0010), *P. abies* SCL8 (MA_52903p0010), *P.abies* SCL9 (MA_10426489p0020), *P.abies* SCL10 (MA_10432093p0010), *P. abies* SCL11 (MA_19310p0010), *P. abies* SCL13 (MA_96029p0010), *P. abies* SCL17 (MA_10255p0010), *P. abies* SCL18 (MA_10430319p0010), *P. abies* SCL23 (MA_73173p0010); *Pinus pinaster* SCL7 (sp_v2.0_unigene8594), *P. pinaster* SCL8 (sp_v2.0_unigene8378), *P. pinaster* SCL9 (sp_v2.0_unigene4531), *P. pinaster* SCL13 (sp_v2.0_unigene1634), *P. pinaster* SCL14 (sp_v2.0_unigene1578), *P. pinaster* SCL15 (sp_v2.0_unigene10599); *Pinus radiata* SCR (KM264388), *P. radiata* SHR (EU044786), *P. radiata* SCL1 (DQ683567), *P. radiata* SCL2 (KM264389), *P. radiata* SCL10 (KM264395), *P. radiata* SCL12 (KM264397); *Pinus taeda* SCR (PITA_000043499-RA), *P. taeda* SHR (PITA_000092405-RA), *P. taeda* SCL1 (PITA_000021589-RA), *P. taeda* SCL5 (PITA_000017225-RA), *P. taeda* SCL6 (PITA_000022609-RA), *P. taeda* SCL8 (PITA_000040137-RA), *P. taeda* SCL9 (PITA_000009055-RA), *P. taeda* SCL10 (PITA_000009053-RA), *P. taeda* SCL11 (PITA_000068827-RA), *P. taeda* SCL12 (PITA_000010887-RA), *P. taeda* SCL15 (PITA_000016257-RA), *P.taeda* SCL16 (PITA_000056676-RA), *P.taeda* SCL18 (PITA_000086415-RA), *P. taeda* SCL19 (PITA_000075302-RA), *P. taeda* SCL20 (PITA_000051405-RA), *P. taeda* SCL21 (PITA_000056428-RA), *P. taeda* SCL22 (PITA_000080766-RA), *P. taeda* SCL23 (PITA_000072928-RA), *P.taeda* SCL24 (PITA_000072831-RA), *P. taeda* SCL25 (PITA_000041536-RA), *P. taeda* SCL26 (PITA_000026833-RA), *P. taeda* SCL27 (PITA_000049193-RA), *P. taeda* SCL28 (PITA_000066307-RA), *P. taeda* SCL29 (PITA_000051712-RA) and *P. taeda* SCL30 (PITA_000035221-RA). PtSCL25 was used as the outgroup. Branches with bootstrap values lower than 500 were collapsed.

To classify the conifer GRAS proteins, a phylogenetic analysis of 52 pine and spruce predicted GRAS protein sequences was performed using a 493 amino acid fragment that included the conserved GRAS C-terminal motif. To avoid possible pseudogenes, only sequences of complete predicted GRAS proteins were included. At least one sequence per conifer GRAS family member, either from pine or from spruce, was included in the analysis. The tree grouped the sequences according to their homology with the classical GRAS protein subfamilies [52] and revealed the existence of an additional group, containing mostly pine sequences, with homology to AtSCL26 (Figure 2, Additional file 2).

The evolutionary relationship of the conifer and angiosperm GRAS proteins was phylogenetically analyzed using 400 amino acid fragments from 100 sequences, including the 52 conifer and 47 angiosperm sequences belonging to the GRAS protein subfamilies [52]. A sequence from *Physcomitrella patens* was used as the outgroup (Additional file 2). The phylogenetic tree showed that the predicted pine GRAS proteins do not cluster into a separate branch, but are distributed among the angiosperm GRAS subfamilies (Additional file 2). The distribution of the conifer sequences was similar to that obtained from the conifer tree, and showed that the AtSCL26 branch is indeed a subfamily that includes 12 pine, two spruce and one *Arabidopsis* sequences (Additional file 2).

In addition to the 52 complete putative GRAS sequences, a total of 37 (*P. taeda*) and 22 (*P. abies*) hypothetical genes encoding partial GRAS proteins were identified (Additional file 1). These could represent pseudogenes resulting from gene duplication, and were more frequent in the SCR, SHR, PAT and AtSCL26 subfamilies of *P. taeda* and in the PAT and DELLA subfamilies of *P. abies* (Additional file 1).

### Conserved motifs and intrinsically disordered N-terminal domains of the pine GRAS proteins

Comparisons of the putative GRAS sequences with previously described proteins revealed that they contain domains characteristic of the GRAS proteins. An analysis of the predicted sequences revealed the presence of the highly conserved VHIID motif, with changes in the valine, leucine and isoleucine residues among members, as well as the PFYRE and SAW motifs in the C-terminal region of the proteins (Additional file 3). Two leucine repeats (LHRI and LHRII) were also identified in the C- terminus. In addition, the LXXLL motif and several additional amino acid residues conserved in other known GRAS members of the protein family, such as the RVER or the LRITG motifs, were identified. The SAW motif contains pairs of the conserved residues RX4E, WX7G and WX10W. Full-length sequences were obtained for 32 members of the multigene family. The N-terminal region of GRAS proteins is variable; however, acidic-residue-rich regions flanking repeated hydrophobic/aromatic residues, similar to those found in PrSCL1 and PrSHR [16,17], were also found in other GRAS proteins from pine (Additional file 4). Homopolymeric stretches of proline and asparagine were only found in SCL5 and SCL12, respectively, while a glycine stretch was found in the GRAS region of the SCL21.

A common feature of the N-terminal region of the analyzed proteins was the enrichment in disorder-promoting residues such as proline, glutamic acid, serine, glutamine, lysine, or in amino acids that are indifferent to disorder or structure, such as alanine, arginine or aspartic acid [53]. A comparison of the disorder profiles of these proteins and the corresponding proteins from angiosperms belonging to the same subfamily shows that the N-terminal region is intrinsically disordered. The intrinsically disordered profile is conserved among members of the same subfamily (Additional file 5).

The structure of the *GRAS* multigene family in pine suggests different roles of individual *GRAS* members in constitutive or induced processes. To extend our previous analysis of the gene expression patterns of *GRAS* genes [16,17], and to show possible differences in spatial and induced expression patterns associated with the maturation-related decline of adventitious root formation, the relative transcript abundance of 13 of the 32 *GRAS* genes was measured by qRT-PCR in organs during vegetative development, during somatic embryo development, at the developmental transition from embryonic to postembryonic development, and during the early stages of adventitious root induction in response to auxin in rooting-competent and non-competent cuttings from *P. radiata*. Genes selected for expression analysis were those initially identified from the EST collection in Genbank, which included members of all of the subfamilies except AtSCL3. *PrSCL1* and *PrSHR* expression levels had already been measured in organs during vegetative development and in hypocotyl cuttings from 21-day-old seedlings during adventitious root formation [16,17].

### Constitutive transcript profiles of *GRAS* genes in organs and changes in the *GRAS* mRNA levels during somatic embryo development and at the embryonic-postembryonic developmental transition

**Figure 3:**
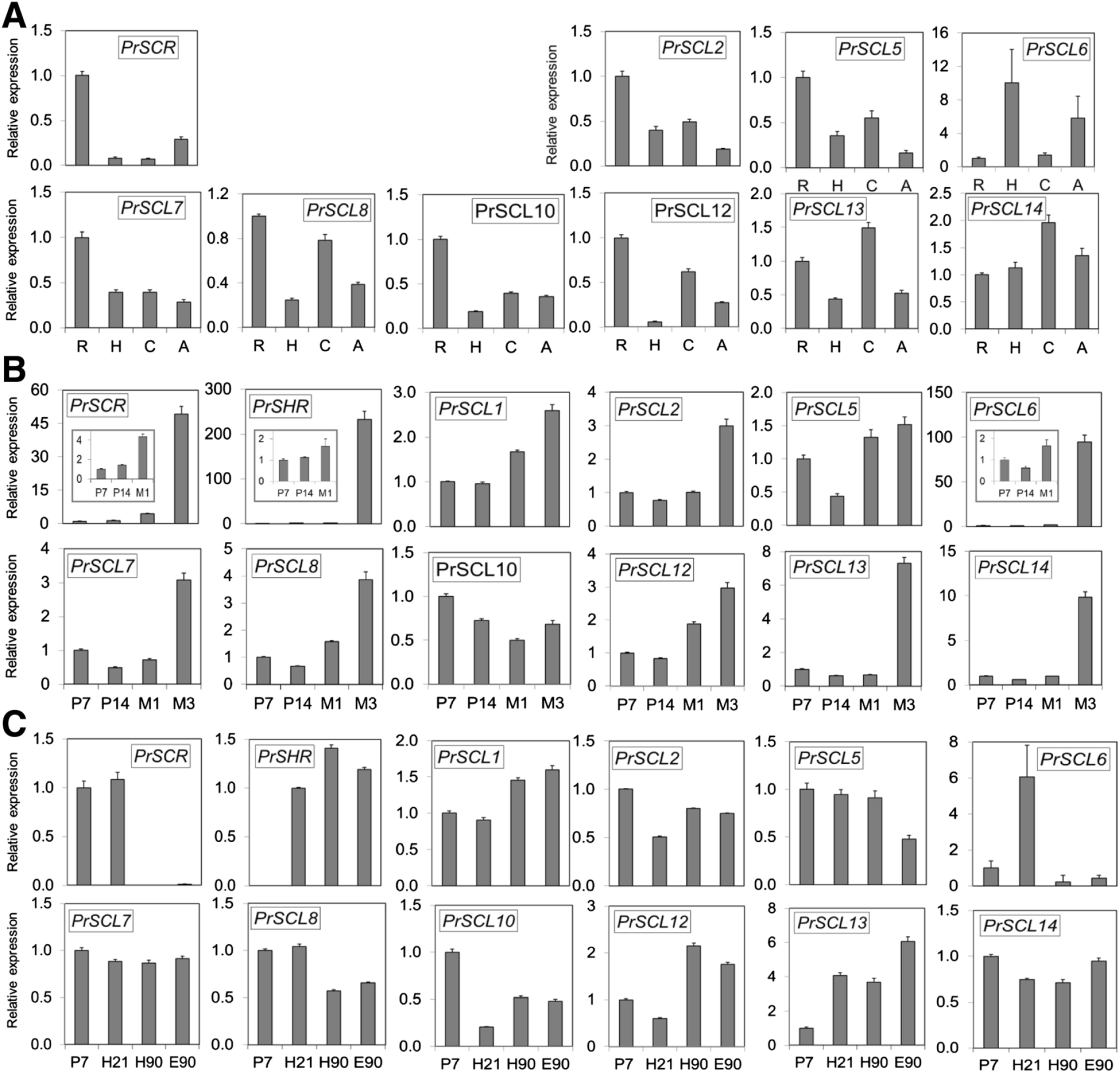
**Expression of** ***GRAS*** **genes in vegetative** ***Pinus radiata*** **organs and at the embryonic-postembryonic develop** **mental transition. A)** Organs from 35-day-old pine seedlings. qRT-PCR was performed using RNAs from roots (R), hypocotyls (H), cotyledons **(C)** or shoot apex nodal segments **(A)**. **B)** Embryo development. qRT-PCR was performed using RNAs from embryogenic masses at 7 (P7) and 14 (P14) days of proliferation, early-maturation embryo (M1) and late-maturation embryo (M3). **C)** Embryonic-postembryonic development. qRT-PCR was performed using RNAs from embryogenic masses at 7 (P7) days of proliferation, rooting-competent hypocotyls (H21) and non-competent hypocotyls (H90) or epicotyls (E90) from seedlings of 21- and 90-day-old seedlings, respectively. A total of 1 μg RNA was reverse transcribed, and 12.5 ng of cDNA was amplified with 400 nM of specific primers. Pine *Ri18S* was used as the control. Results are expressed as mean values of the relative expression to roots **(A)** or P7 **(B and C)** ± SE from at least three biological replicates. Insets in B show details of early developmental stages. Results of *PrSHR* expression in C are expressed as mean values of relative expression to H21. Expression levels of *PrSCL1* and *PrSHR* had already been measured in organs during vegetative development [16,17]. Expression of *PrSCL16* was not detected in any of the RNA samples tested. SCL, SCARECROW-LIKE; SHR, SHORT-ROOT.

To characterize the expression patterns of *GRAS* genes in different organs during vegetative development, RNAs isolated from roots, hypocotyls, shoot apex nodal segments (including the apical meristem, young needles and shoot segments) and cotyledons from 35-day-old pine seedlings were used. Results were expressed as values relative to the expression levels in roots (Figure 3A). Additionally, changes in *GRAS* mRNA levels were also studied during somatic embryo development (Figure 3B) and at the embryonic-postembryonic developmental transition (Figure 3C). To that end, mRNA levels were analyzed in developing somatic embryos and in organs of embryonic and postembryonic origin from seedlings of different ages. Zygotic embryos are very difficult to isolate at specific developmental stages, but *P. radiata* somatic embryos show a very similar developmental pattern; therefore, specific developmental stages can be defined and isolated. RNAs isolated from embryogenic masses in the proliferation stage, from somatic embryos at the early and late maturation stage, and from rooting-competent and non-competent hypocotyl or epicotyl cuttings from 21- and 90-day old seedlings were used to analyze the expression patterns during embryo development and at the embryonic-postembryonic developmental transition. Results were expressed as values relative to the expression in embryogenic masses after 7 days in proliferation medium. The expression of *PrSCL16* was not detected in any of the RNA samples tested.

Most *GRAS* genes showed relatively high mRNA levels in roots, except *PrSCL6*, which showed relatively high levels in hypocotyls and in the shoot apices of young seedlings. *PrSCL13* and *PrSCL14* also showed relatively higher expression levels in cotyledons. The relative abundances in other tissues depended on the individual GRAS genes (Figure 3A).

The analysis of *GRAS* transcript profiles during somatic embryo development (Figure 3B) showed that the transcript levels of all *GRAS* genes, except *PrSCL1*0, which showed relatively high levels in embryogenic masses, were significantly higher in the embryos at the late maturation stage than in other stages. mRNA levels of *PrSCR*, *PrSHR*, *PrSCL1*, *PrSCL6*, *PrSCL8* and *PrSCL12* increased between two and four times in the embryo during the early maturation stage (Figure 3B). The analysis of *GRAS* transcript profiles at the developmental transition from embryonic to postembryonic development (Figure 3C) showed that *PrSCR* and *PrSCL6* maintained relative high levels in rooting-competent hypocotyls from 21-day-old seedlings, whereas the other *GRAS* genes also maintained relatively high levels in rooting-non-competent hypocotyls and epicotyls from older seedlings (Figure 3C).

### Transcript profiles of *GRAS* genes during adventitious rooting in competent and non-competent stem cuttings

**Figure 4:**
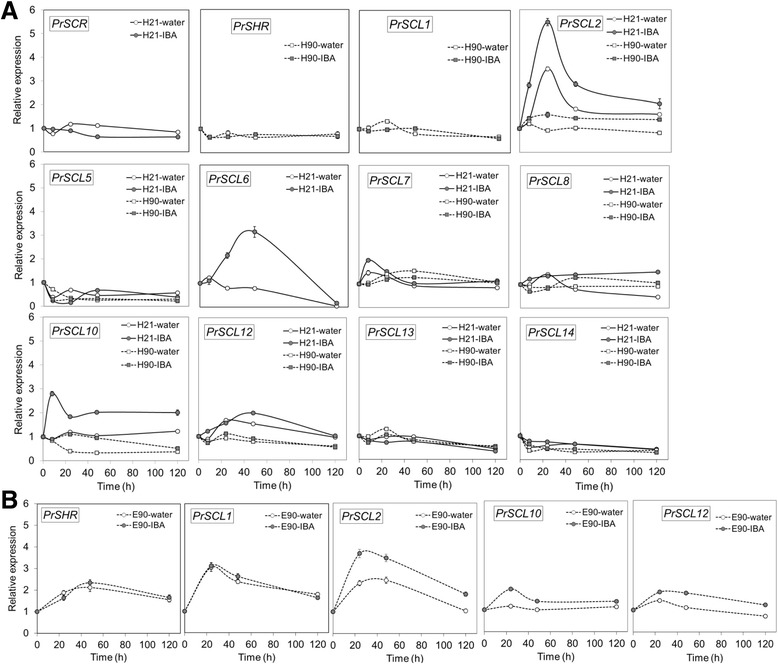
**Expression of** ***GRAS*** **genes during adventitious root formation in** ***Pinus radiata*** **. A)** qRT-PCR was performed using RNAs from rooting-competent hypocotyls (H21) and non-competent hypocotyls (H90) from 21- and 90-day-old seedlings, respectively. **B)** qRT-PCR was performed using RNAs from non-competent epicotyls (E90) from 90-day-old seedlings. RNA was extracted from the base of hypocotyl (H) or epicotyl (E) cuttings treated with 10 μM indole-3-butyric acid at the indicated times. Hypocotyl or epicotyl cuttings maintained in water were used as controls. A total of 1 μg RNA was reverse transcribed, and 12.5 ng of cDNA was amplified with 400 nM of specific primers. Pine *Ri18S* was used as the control. Results are expressed as mean values of relative expression to time 0 ± SE from at least three biological replicates. Expression levels of *PrSCL1* and *PrSHR* had already been measured in competent hypocotyls from 21-day-old seedlings during adventitious rooting [16,17]. Expression of *PrSCL16* was not detected in any of the RNA samples tested. SCL, SCARECROW-LIKE; SHR, SHORT-ROOT.

A possible role of *GRAS* genes in the loss of rooting capacity was analyzed by assessing their temporal expression patterns in response to auxin in hypocotyl and epicotyl cuttings from 21- and 90-day-old seedlings (Figure 4). Cuttings were treated with 10 μM indole-3-butyric acid (IBA) [16,17]. Then, transcript profiles were analyzed in the basal ends of cuttings during the initial 24 h, at 48 h and 5 d after the onset of the treatment, and compared with control tissues at their time of excision (time 0). Data are presented as mRNA levels normalized to *ribosomal 18S* [16,17] and as fold inductions relative to their time of excision (time 0). The expression of *PrSCL16* was not detected in any of the RNA samples tested.

Several patterns of expression were observed in hypocotyl cuttings from 21-day-old seedlings during adventitious rooting (Figure 4A). *PrSCL2*, *PrSCL6*, *PrSCL7*, *PrSCL10* and *PrSCL12* mRNA levels increased in the presence of exogenous auxin, similar to *PrSCL1*’s expression pattern [16]. *PrSCL2* and *PrSCL12* mRNA levels were even increased in the absence of exogenous auxin similar to *PrSHR*’s expression pattern [17]. *PrSCR*, *PrSCL5*, *PrSCL8*, *PrSCL13* and *PrSCL14* mRNA levels did not show any change in their expression level during the root-induction process. No *GRAS* genes showed increases in mRNA levels in the absence or presence of exogenous auxin in the rooting-non-competent hypocotyl cuttings from 90-day-old seedlings (Figure 4A). *PrSCR* and *PrSCL6* mRNAs were not detected in non-competent hypocotyls under root-induction conditions (Figure 4A).

**Figure 5:**
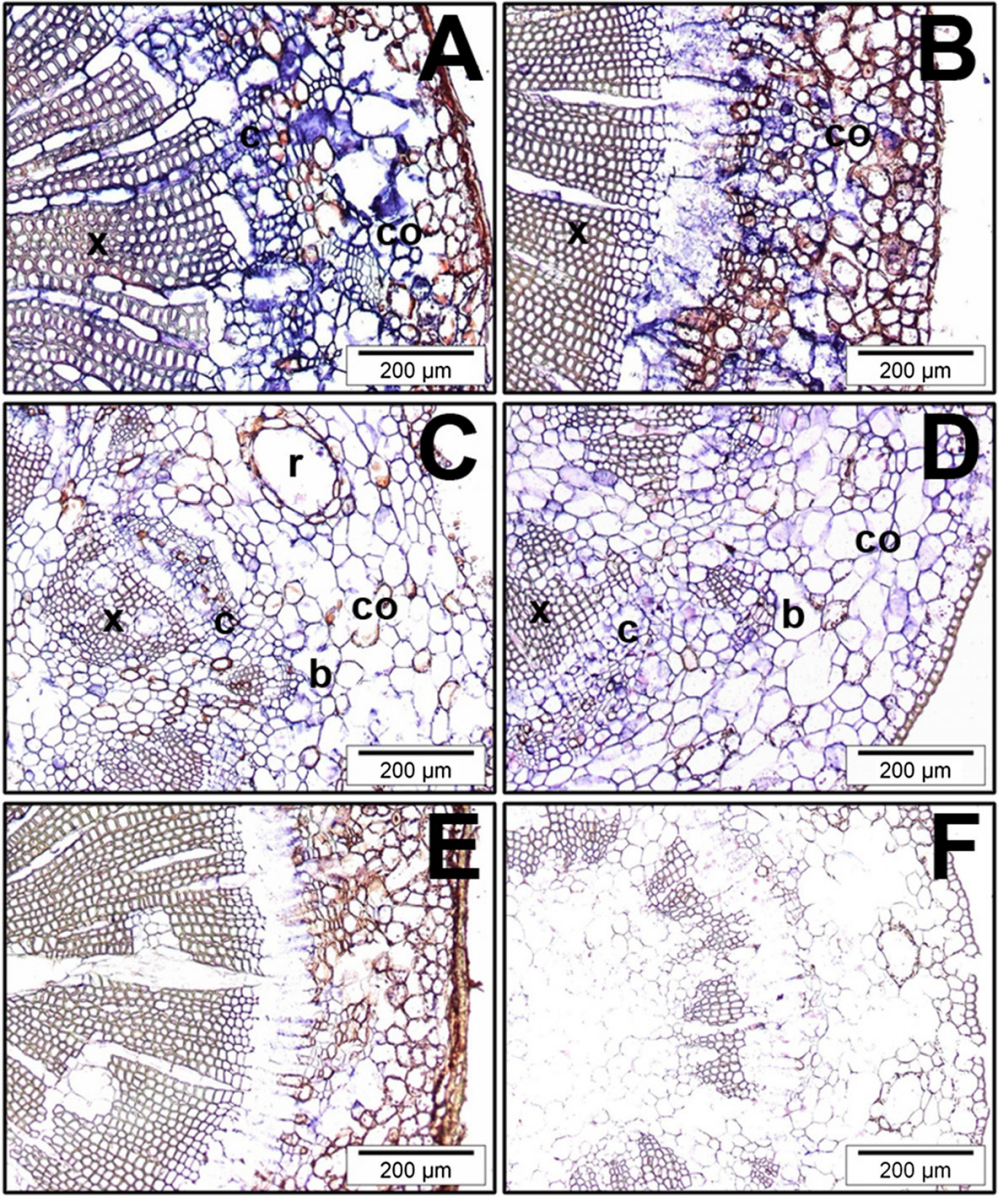
***In situ*** **localization of** ***Pinus radiata SHORT-ROOT*** **(** ***PrSHR*** **) mRNA. A**, **B)** Transverse sections of hypocotyls from 90-day-old seedlings at time 0 **(A)**, and after 24 h of culture in the presence of 10 μM indole-3-butyric acid (IBA) **(B)**. **C**, **D)** Transverse sections of epicotyls from 90-day-old seedlings at time 0 **(C)**, and after 24 h of culture in the presence of 10 μM IBA **(D)**. The sections were hybridized with an RNA probe obtained by *in vitro* transcription of *PrSHR* in either the antisense **(A, B, C, D)** or sense **(E, F)** orientation. Note the absence of hybridization in the controls. Similar results were obtained using an RNA probe obtained by *in vitro* transcription of *PrSCL1* in either the antisense or sense orientation. ab, axillary bud; c, cambial region; co, cortex; r, resin canal; x, xylem. *In situ* localization of *PrSCL1* and *PrSHR* had already been described in competent hypocotyls from 21-day-old seedlings during adventitious rooting [17]. SCL, SCARECROW-LIKE.

Several expression patterns were also observed in epicotyl cuttings from 90-day-old seedlings during adventitious rooting (Figure 4B). *PrSCL2*, *PrSCL10* and *PrSCL12* mRNA levels increased in the presence of exogenous auxin, while *PrSHR*, *PrSCL1* and *PrSCL2* mRNA levels even increased in the absence of exogenous auxin. *PrSCR* and *PrSCL6* mRNAs were not detected in the presence or absence of auxin. *PrSCL5*, *PrSCL7*, *PrSCL8*, *PrSCL13*, *PrSCL14* and *PrSCL16* were not tested for in epicotyls. *PrSCL2* mRNA levels increased in the absence of exogenous auxin in both rooting-competent hypocotyl cuttings from 21-day-old seedlings and rooting-non-competent epicotyl cuttings from 90-day-old seedlings. However, the increase in transcript levels was significantly higher in the presence of exogenous auxin (Figures 4A, B).

The expression of two genes, *PrSCL1* and *PrSHR*, which are associated with auxin-dependent and auxin-independent signaling pathways, respectively, in rooting-competent cuttings [16,17] were also analyzed by *in situ* hybridization in non-competent cuttings. In our previous work, it was shown that increased transcript levels of both genes accumulated in the rooting-competent tissues of hypocotyls from 21-day-old seedlings after 24 h of root induction [17]. These genes were not predominantly expressed in the cambial region of non-competent hypocotyls or epicotyls at the time of excision, nor under rooting conditions (Figures 5A, B, C, D). No specific tissue-localization was observed in any samples during adventitious rooting. No signal was observed when tissues were hybridized sense-oriented probes (Figures 5E, F).

**Figure 6:**
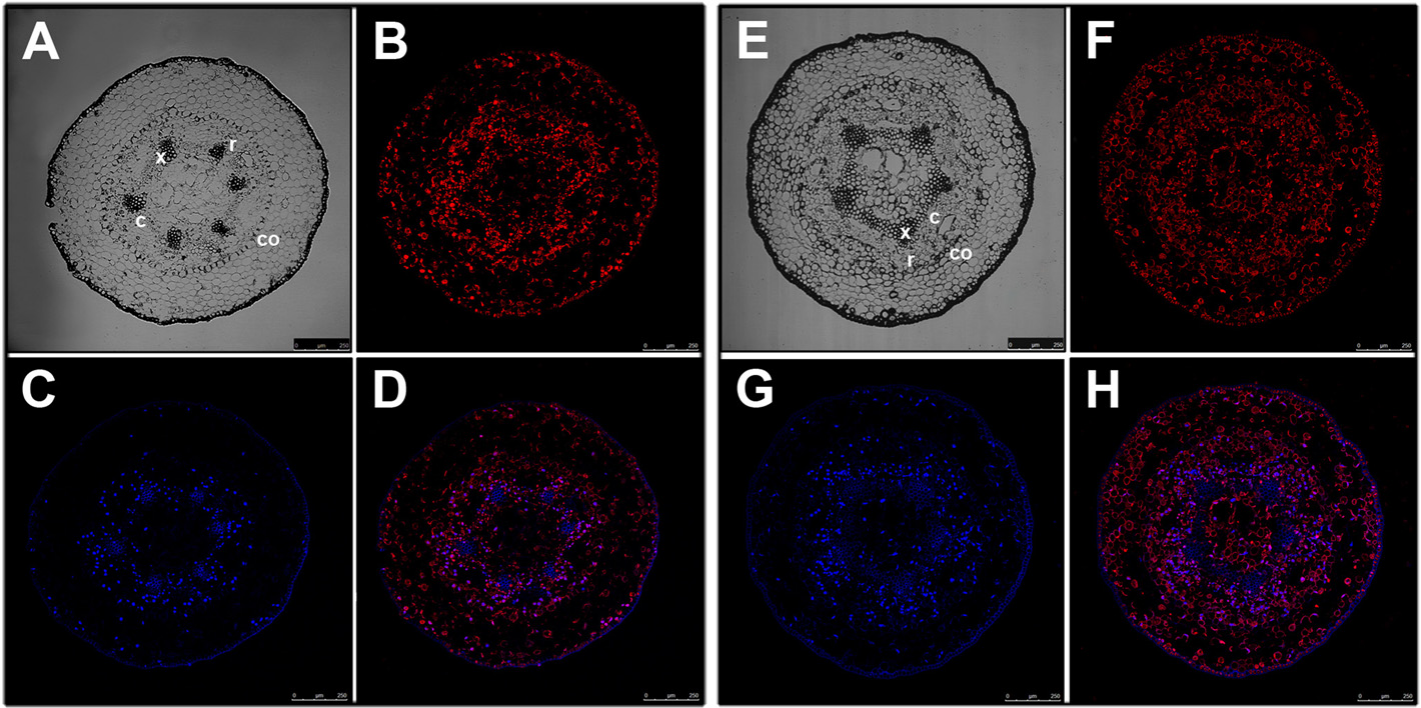
**Endogenous distribution of indole-3-acetic acid (IAA) in hypocotyl cuttings from 21-day-old** ***Pinus radiata*** **seedlings.** Transverse sections from the base of hypocotyls after 24 h of culture in the presence of 10 μM indole-3-butyric acid (IBA) **(A, B, C, D)** or in the presence of 10 μM IBA + 10 μM 1-N-naphthylphthalamic acid **(E, F, G, H)**. **A, E)** Differential interference contrast (DIC) image, **B**, **F)** Immunodetection of IAA, **C**, **G)** DAPI nuclear staining, **D**, **H)** merged immunodetection of IAA and DAPI staining. c, cambial region; co, cortex; r, resin canal; x, xylem.

**Figure 7:**
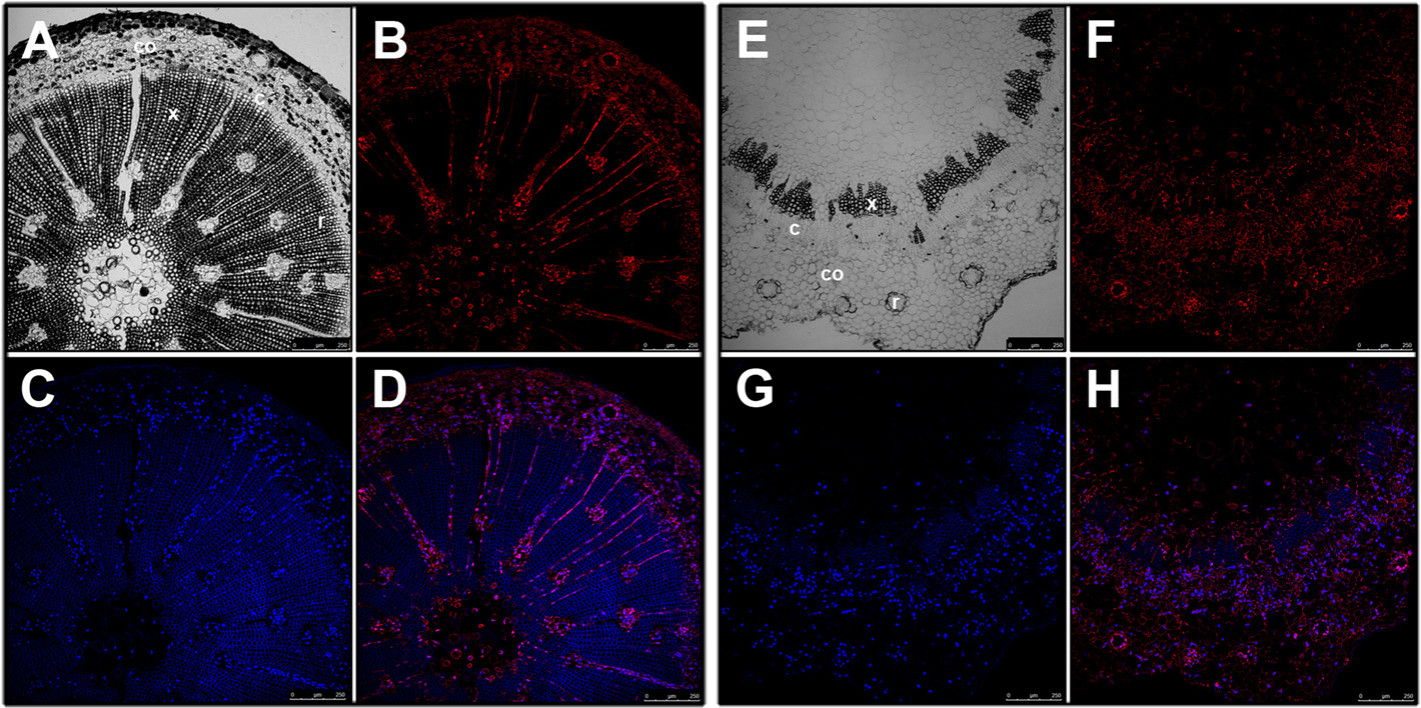
**Endogenous distribution of indole-3-acetic acid (IAA) in hypocotyl and epicotyl cuttings from 90-day-old** ***Pinus radiata*** **seedlings.** Transverse sections of the base of hypocotyls **(A, B, C, D)** and epicotyls **(E, F, G, H)** after 24 of culture in the presence of 10 μM indole-3-butyric acid. **A**, **E)** Differential interference contrast (DIC) image, **B**, **F)** Immunodetection of IAA, **C**, **G)** DAPI nuclear staining, **D**, **H)** merged immunodetection of IAA and DAPI staining. c, cambial region; co, cortex; r, resin canal; x, xylem.

### Auxin distribution in rooting-competent and non-competent cuttings in the presence of exogenous auxin and polar auxin transport inhibitors

Auxin-dependent adventitious root formation in pine is associated with a directional flow of auxin in combination with the competition of neighboring cells for free auxin [10]. Tissue-specific auxin gradients can elicit specific cellular responses. The role of the endogenous auxin distribution in rooting-competent and non-competent tissues during adventitious root formation was addressed by analyzing the indole-3-acetic acid (IAA) distribution. Experiments were performed at the time of excision and after 24 h with or without exogenous auxin. The IAA distribution was analyzed by an immune-cytochemical approach using antibodies raised against IAA (Figure 6, Figure 7). IAA was mostly located in the cambial region of rooting-competent hypocotyls, including the cells positioned centrifugal to the resin canals after excision, and during the initial 24 h of root induction (Figures 6 A, B, C, D). Treating rooting-competent hypocotyls with 1-N-naphthylphthalamic acid (NPA), a polar auxin transport inhibitor, resulted in the mislocalization of endogenous auxin, which was also distributed in the pith, in the vascular cylinder and in the cortex (Figures 6 E, F, G, H). No auxin accumulation was detected in the cambial cells in non-competent hypocotyls or epicotyls. Auxin was mainly located in the xylem parenchyma of hypocotyls (Figures 7 A, B, C, D), and in the cortex of epicotyls (Figures 7 E, F, G, H). No signal was observed when tissues were hybridized in the absence of the antibody (Additional file 6).

## Discussion

Plants do not lose their developmental potentialities during differentiation and retain a certain level of plasticity [54], either by maintaining pro-embryonic or meristematic cells in the adult tissues or by a major developmental reprogramming to acquire the embryonic or meristematic status [55]. The plasticity of plant tissues results in the regenerative capacity of cells other than those of meristem, lateral root initials or zygotes.

A decline in the regenerative capacity of somatically differentiated cells in an ectopic location is associated with age and maturation in forest tree species [13]. Efforts have been made to identify genes associated with plant cell fate switches [34,38]; however, pluripotency or indeterminacy genes, with high expression levels in non-differentiated embryonic cells or at the very early stages of development, significantly reduced or even no expression levels in adult tissues that have lost their regenerative capacities, but maintained in tissues with regenerative capacities or induced after the reprogramming of adult cells during regeneration [56], have not been described. We have made use of embryogenic cultures maintained under non-differentiated proliferating conditions or subjected to differentiation, as well as adult tissues from plants of different ages showing different adventitious rooting capacities in response to auxin, to identify genes, changes in gene expression levels and regulatory mechanisms associated with the competence and reprogramming of adult tissues to form adventitious roots in pine (Figure 1).

In our previous work, two members of the *GRAS* gene family of *P. radiata*, *PrSCL1* and *PrSHR*, were associated with the adventitious root formation in rooting-competent cuttings [16,17]. GRAS proteins are involved in a diverse suite of physiological and developmental processes ranging from light and hormone signal transduction to organ identity and tissue differentiation [57,58]. Among them, SCR and SHR are involved in root patterning, establishing the quiescent center’s identity and in maintaining the stem cell status of the initial cells in the root meristem [43,44]. Additionally, they have been involved in root tip regeneration [59] and in cell reprogramming [38]. GRAS proteins have been identified as homologous proteins to the STAT proteins in animals [60], which have also been associated with differentiation, reprogramming and regeneration [61,62].

A large gene family encodes GRAS proteins in pine. Supporting cDNAs were identified for at least 32 unique members in *P. taeda* (Additional file 1), a number close to that described in *P. abies* (Additional file 1) and *Arabidopsis* [63-65], higher than the number described in *P. pinaster* and *P. glauca* (Additional file 1) [65,66], and lower than the number described in *Oryza sativa*, *Populus trichocarpa* and *Brassica rapa* [63,65,67]. Eighteen members were identified in *P. radiata* (Additional file 1) [16,17]. Pairwise sequence similarities among predicted polypeptides for each GRAS member of the different pine and spruce species confirmed that they may represent intra- or inter-specific alleles of the same genes, similar to those described for other gene families in conifer species [68,69]. The proteins belonging to the AtSCL26 group showed a lower degree of identity, which could be related to a high number of duplication events, perhaps to acquire new functions (Figure 2 and Additional file 2).

A phylogenetic analysis showed that conifer GRAS proteins do not form a separate cluster (Figure 2 and Additional file 2) and most are included in the major GRAS subfamilies [16,17,52,57,58]. The HAM family contains the AtSCL26 subfamily, which may be the result of a high number of duplication events for conifer sequences compared with their angiosperm counterparts. Conifers diverged from angiosperms 300 million years ago [70]. The phylogenetic relationship between conifers and angiosperms highlights the ancient diversification of this family, which may precede the transition to terrestrial environments, as suggested by Engstrom [71] based on comparisons among GRAS proteins from angiosperms, bryophytes and lycophytes, but not gymnosperms. The ancient diversification and the non-clustering of conifer sequences suggests functions or modes of action for these proteins in primary constitutive or induced processes [72-77].

An analysis of the polypeptide sequences shows a high degree of conservation in the representative GRAS core motifs (Additional file 3) [52,57,58], which are involved in transcriptional regulation, indicating that the transcriptional regulatory machinery is also conserved in conifers. The N-terminal domain of the predicted GRAS proteins is highly variable in pine (Additional file 4), similar to the N-terminal domain of angiosperm GRAS proteins [57,58]. Homopolymeric stretches, such as those characterizing angiosperm GRAS proteins [63,64,78], were not found in conifer GRAS proteins, except for the proline and asparagine stretches found in PtSCL5 and PtSCL12. The amino acid compositional profile of the N-terminus of GRAS proteins from pine is very similar to that of the intrinsically disordered proteins and contains an enrichment in disorder-promoting residues (Additional files 4 and 5). However, the C-domain shows a compositional profile similar to that of fully structured proteins, as described for other GRAS proteins [57,58]. Disordered proteins lack a well-defined three dimensional structure, resulting in an extreme structural flexibility that enables them to form highly specific complexes with different proteins or nucleic acids in a reversible and transient low-affinity interaction, depending on the changing physiological, developmental or environmental conditions [53,79]. Intrinsic disorder has been described for several families of plant transcription factors, and intrinsically disordered proteins have been associated with key cellular and signaling processes [80-82]. The intrinsic disorder could be a way to increase functional diversity and the complexity of biological networks without increasing the size of the families, or even, the size of the genome, and it was proposed as the mechanism involved in the functional divergence within GRAS subfamilies [57,58]. Despite the highly variable sequence of the N-terminus, GRAS proteins in pine show conserved disordered profiles when compared with GRAS proteins from angiosperm species of the same subfamily (Additional file 5) [57,58]. This is in agreement with previous suggestions [83,84], indicating that the pattern of protein disorder could be more conserved through evolution than the amino acid sequence in the N-terminus. Similar results have been described for the mammalian Myc proteins [85]. Consequently, mutations that do not affect the general disorder pattern would allow the conservation of specific protein interactions and, hence, functions.

The conservation of the protein motifs and structures, the absence of a particular conifer subfamily, and the intrinsically disordered N-terminal domain can account for the versatile roles of these proteins in tree biology and for the molecular mechanisms regulating their expression levels and functions. The dynamic ability of intrinsically disordered proteins to recognize multiple molecular partners reveals the need for a synchronous spatio-temporal connection between the functionally appropriate *GRAS* genes and proteins participating in specific functions.

The expression of *GRAS* genes in the different organs, at the embryonic-postembryonic developmental transition, as well as during adventitious rooting, in response to auxin showed unique and overlapping patterns, indicating a differential regulation and tissue-specific functions (Figure 3 and Figure 4). Individual genes within each group may have acquired different and specialized functions, some of which may relate to competence and the reprogramming of adult cells to form adventitious roots. Many pine *GRAS* genes show relatively high levels of mRNA during the transition from the polarization stage (M1) to the late maturation stage (M3), indicating that they play roles in embryo development (Figure 3B). A subset of these genes, *PrSCR*, *PrSHR*, *PrSCL1*, *PrSCL6*, *PrSCL8* and *PrSCL12*, increase their mRNA levels during the early maturation stage. At this stage, embryo polarization occurs, but tissue differentiation has not been yet completed; therefore, these tissues, along with the proliferating embryogenic masses, may be sources of non-determined or pluripotent cells associated with the establishment of tissue domains [47]. However, *PrSCL10* shows a relatively high level of mRNA after 7 days of proliferation, when initials are developed. Consequently, these genes play key roles in the initial establishment of embryo tissue domains or hormone gradients [86]. Among them, *PrSCR* and *PrSCL6* are highly expressed in organs of embryonic origin, such as hypocotyls, cotyledons and shoot apices. Additionally, *PrSCR*, along with *PrSHR*, *PrSCL1* [16,17], *PrSCL5*, *PrSCL7*, *PrSCL8* and *PrSCL12* showed relatively higher levels of mRNAs in roots than in any other organs tested during vegetative development, indicating a role in the roots (Figure 3A). These results suggest that the expression of these genes is not only restricted to embryonic development but extended to other processes. We then analyzed if the expression levels of genes associated with the early stages of embryo formation could be significantly reduced or even non-existent in cuttings that have lost their rooting capacity, but maintained in rooting-competent tissues or induced after the reprogramming of adult competent cells to form adventitious roots.

*PrSCL6* and *PrSCR* maintain relatively high levels of mRNA in rooting-competent hypocotyls, while other *GRAS* genes are expressed in both rooting-competent hypocotyls and rooting-non-competent hypocotyls or epicotyls (Figure 3C). These results indicate that *PrSCL6* and *PrSCR*, in addition to their functions in embryo development, are associated with an embryonic characteristic that could result in the competence for adventitious organogenesis in cuttings. An analysis of these genes during adventitious root formation in competent and non-competent tissues indicated that *PrSCL6* is auxin-induced in rooting-competent hypocotyls only, and the expression increases during the initial 48 h, which is the time required for auxin action and for the reorganization or dedifferentiation of cambial cells [10,11,16,17]. *PrSCL6* is not detectable in rooting-non-competent hypocotyls or epicotyls (Figures 4 A, B). Similar results are also obtained when *PrSCR* expression is analyzed; however, *PrSCR* is not induced in rooting-competent hypocotyls, but its mRNA levels are maintained at higher levels in these tissues than in non-competent hypocotyls or epicotyls, in which *PrSCR* is not detectable during the initial stages of rooting (Figures 4A, B). Therefore, both genes could be associated with embryonic cells or with the very early stages of development. Their mRNA levels were significantly reduced or even lost in older and more mature rooting cuttings that had lost their rooting capacities, but were maintained in competent hypocotyls or increased after the reprogramming of adult competent cells during adventitious root formation. This would make them candidate genes for rooting competence and cell reprogramming.

The mRNA levels of other genes, such as *PrSHR PrSCL1*, *PrSCL2, PrSCL10* and *PrSCL12,* changed in an auxin-, age- or developmental-dependent manner during adventitious rooting in competent and non-competent cuttings (Figures 4A, B) [16,17]. The localized increases of *PrSHR* and *PrSCL1* mRNAs in competent tissues [17], which were not detected in non-competent hypocotyl or epicotyl cuttings (Figure 5), suggests their involvement in adventitious rooting. The expression profiles in epicotyls could be associated with the presence of meristematic tissues in these cuttings, such as the shoot axillary meristem or cambium [46,87]. The tissue localization of *PrSCL2*, *PrSCL10* and *PrSCL12* mRNAs would help to show the roles of their mRNA variations in adventitious rooting. Other pine *GRAS* genes do not seem to be related to the adventitious rooting response (Figure 4A).

These results indicate that high levels of *PrSCR* and *PrSCL6* may be related to the degree of determination, competence, and/or the reprograming capacity of tissues to form adventitious roots, while other genes that are also expressed or induced, such as *PrSHR*, *PrSCL1*, *PrSCL2*, *PrSCL10* or *PrSCL12*, could be involved in transcriptional regulatory networks associated with auxin-dependent and auxin-independent pathways in an age- or developmental-dependent manner. Therefore, the participation of these genes in determining whether cells become roots in competent tissues cannot be discarded. The low expression levels of *PrSCR* and *PrSCL6* could make these rate-limiting steps in competence and in auxin-induced processes. Additionally, all these genes are expressed or induced at the very early stages of adventitious root formation before the onset of cell divisions leading to the formation of a root meristem. A set of 26 of the 500 transcription factors expressed during the early events, which occur in the initial 24 h, leading to the regeneration of *Arabidopsis* plants from protoplasts, were not expressed during senescence [38].

The functional analysis of genes based on their subfamilies indicates a possible role in determination and patterning. *SCR* and *SHR* are involved in root meristem determination [43,44] and, along with other transcription factors, have been involved in reprogramming in *Arabidopsis* [38]. Additionally, *PrSCL1*, which may be related to the rooting process, has been associated with the adventitious and lateral root meristem of pine and chestnut [16,46], and with the shoot axillary meristem in chestnut [46]. Also, *PrSCL2* and *PrSCL12* are members of two GRAS subfamilies (the Ls and HAM families, respectively), which have been associated with the determination of lateral meristem [88,89]. Although *PrSCL10* is included in the PAT family of GRAS proteins, which is associated with light responses [90], members of this subfamily have also been associated with cell defense [91,92]. Therefore, this subfamily is also functionally diverse. Overall, a role in adventitious root competence, reprogramming and determination could be envisaged for a subset of the pine *GRAS* genes.

The asymmetrical increases of *PrSCL1* and *PrSHR* transcript levels previously described in the cambial region and rooting-competent cells [17] were not detected in non-competent cuttings (Figures 5 A, B, C, D). In these cuttings, expression spread into the cortex and dividing cells. The asymmetrical increase in mRNA during the earliest stages of adventitious root formation in similar cell types at different developmental stages suggests the presence of specific cellular signaling pathways or specific factors in pine, perhaps distributed in cell-type- and developmental-stage-specific contexts in the tissues involved in rooting, which could be crucial for rooting capacity [18,46]. The nature of these signaling pathways or factors is unknown. *De novo* organ formation and cell specification are processes involving rearrangements of tissue polarity, with the temporal and spatial distribution of auxin being a very important player, contributing to tissue polarization and patterning [93]. No differences in auxin uptake, accumulation or metabolism were found between rooting-competent and non-competent hypocotyls and epicotyls at the base of the cuttings [10]. However, an asymmetric auxin distribution was detected in rooting-competent tissues after excision and was maintained during the initial 24 h of root induction (Figures 6 A, B, C, D) at locations where *PrSHR* and *PrSCL1* are expressed [17]. An asymmetrical distribution was not observed in non-competent hypocotyls or epicotyls (Figure 7). Treatments with NPA, which inhibits rooting [10] and does not change the number of cell layers in the vascular cylinder, cortex or pith, changed the auxin distribution pattern (Figures 6 E, F, G, H), indicating that polar auxin transport, which resulted in an accumulation of auxin at the base of the cutting [10], as well as auxin localization and distribution at the tissue or cellular levels. This result indicated that rooting-competent tissues could retain an intrinsic capacity to maintain or accumulate auxin after excision, which could be crucial for rooting. The cellular capacity of initial cells to produce auxin gradients may be a mechanism involved in the determination and maintenance of meristem, the induction of lateral primordia at the shoot meristem, and the formation of lateral roots or adventitious roots [20,94-96]. Auxin distribution largely depends on the dynamic expression and subcellular localization of the PIN auxin-carrier proteins [97]. However, PIN activity can be modulated by endogenous or exogenous signals, such as other hormones, stress or tissue-specific factors, to trigger developmental decisions that could initiate regeneration by triggering cell fates or other local changes [37,87,98-101]. No differences in the wounding stress response were observed between competent and non-competent cuttings [102]; therefore, other tissue-dependent signals could also trigger re-patterning either by inducing cell-fate respecification or by re-establishing the auxin distribution. Transcription factors are main players in regulatory modules controlling auxin gradients, positional information and the development of polarity fields, resulting in a cross regulatory network involved in organ formation [103-107]. The differential expression of genes, such as *PrSCR* and *PrSCL6*, in rooting-competent and non-competent cuttings, as well as the differential responses of genes, such as *PrSCL1* or *PrSHR* [Figure 4, [16-18] to exogenous auxin during adventitious rooting may indicate the local involvement of specific GRAS transcription factors in the rooting capacity by participating in the auxin distribution, control of cell-type divisions, or other mechanisms. The auxin-related increase of *PrSCL1* mRNA in competent tissues after 24 h of root induction [17] could be associated with auxin localization in these tissues at the same time (Figures 6A, B, C, D). The overlap in the temporal and spatial distribution of auxin (Figures 6A, B, C, D), and the increase of the auxin-independent *PrSHR* mRNA [17] could indicate a possible crosstalk between the signaling pathways, perhaps establishing response domains that activate a cascade of other *GRAS* genes or root determining factors before the resumption of cell divisions. Sabatini et al. [44] proposed that *SCR*- and *SHR*-expressing cells are competent to acquire quiescent center identity, with auxin distribution being the cue that specifies a subset of cells within the SCR or SHR expression domains. However, the SHR pathway regulates root development through a transcriptional regulatory network and also by affecting the expression of genes involved in cytokinin and auxin signaling in *Arabidopsis*, resulting in the fine-tuning of hormonal responses [87,99,108]. Additionally, formative divisions that generate the root’s ground tissue are controlled by SHR in *Arabidopsis*, which specifically regulates the spatiotemporal activation of specific genes involved in cell division, and by SCR, both activating a D-type cyclin involved in formative divisions [109].

## Conclusions

Adventitious root forming treatments induce root meristem patterning genes, such as *GRAS* genes, before the onset of cell division in competent cells. The same *GRAS* genes also may play a role during the earliest stages of embryogenesis, initial-forming and polarization. The capacity to maintain or recruit root meristem or embryonic programs in response to a specific stimulus seems to be key in switching cells into different developmental programs, both in herbaceous and woody plants, including forest tree species [34-36,38,110]. However, whether this pattern of expression represents a maintenance, a dedifferentiation or a transdifferentiation to an embryonic or root identity, or it represents a different adult developmental program unique to regeneration, as was described in *Arabidopsis* [111], remains unknown.

## Methods

### Plant material, root induction and somatic embryogenesis

Pine (*P. radiata* D. Don) seeds were germinated and seedlings were grown as previously described [16]. The seedlings were treated daily with water, and, after 21 days, weekly with 2 g/l of a commercial soluble fertilizer (NPK 20-7-19 [w/w/w]). Cuttings for adventitious root induction were prepared according to [16]. Briefly, hypocotyl cuttings from 21-day-old seedlings, including the intact epicotyl, and hypocotyl or epicotyl cuttings from 90-day-old pine seedlings were prepared by severing the hypocotyl or epicotyl at its base, and trimming it to a length of 2.5 cm from the cotyledons (hypocotyls) or from the apical buds (epicotyls). All but one apical tuft of needles were removed from the epicotyls to obtain a foliar surface similar to that of the hypocotyls. Root induction was conducted by exposing the cuttings to 10 μM IBA continuously (Figures 1 E, F, G). Cuttings without IBA treatment were used as controls. IBA was obtained from Sigma (St. Louis, MO, USA) as IBA-K and dissolved in distilled water. For experiments on auxin immunolocalization, hypocotyls from 21-day-old seedlings were also treated with NPA in the presence and absence of auxin for 24 h. NPA was obtained from Duchefa (Haarlem, Netherlands) and dissolved in DMSO. Hypocotyls treated with DMSO were also used as controls in these experiments. Conditions for root induction were the same as described for seedling growth [16]. Embryonal suspensor masses and somatic embryos were also used for analyses (Figures 1 A, B, C, D). Embryogenic line M95, provided by Dr. Christian Walter (Scion, Rotorua, New Zealand), was proliferated and maintained by bi-weekly subcultures of individual clumps onto EDM6 medium [112]. For somatic embryo maturation, 500 mg of embryogenic tissue was suspended in 25 ml of EDM6 liquid medium. The tissue suspension was collected pouring 5 ml aliquots onto a filter paper disk (80 g/m2 43–48 μm; Filter Lab, ANOIA; Barcelona, Spain) in a Büchner funnel. A vacuum pulse was applied to drain the liquid, and the filter paper with the attached cells was placed into a 90 mm diameter Petri dish with maturation medium, which was based on the formulation of EMM1 medium [112] supplemented with 15 mg · L^−1^ abscisic acid, 30 g · L^−1^ sucrose and 6 g · L^−1^ Gelrite®. Cultures were maintained in darkness at 23 ± 1°C. The pH of the media was adjusted to 5.8 before autoclaving. Solutions of amino acids and abscisic acid were filter sterilized and added to the cooled autoclaved medium.

### RNA extraction, quantification and cDNA synthesis

For analysis of gene expression during adventitious rooting, 30 basal segments, 1 cm long, of the hypocotyl or epicotyl cuttings were pooled from each treatment and time point as specified in each experiment, immediately frozen in liquid nitrogen and stored at −70°C until used for RNA isolation. Total RNA isolation and quantification from cuttings have been previously described [16]. RNA was also extracted from different organs of plant seedlings as specified in each experiment. Samples of embryogenic tissues were used for expression analysis experiments at different stages of development: proliferative tissues 7 and 14 days after the last subculturing to fresh proliferation medium (Figures 1 A-B), somatic embryos at the early maturation stage of development (Figure 1C) and somatic embryos at the late maturation stage (Figure 1D). Tissues were frozen in liquid nitrogen and stored at −70°C until used for RNA isolation. Total RNA was extracted using the RNeasy® Plant mini kit (Qiagen, Hilden, Germany), following manufacturer’s instructions. Between 50 and 100 mg of embryogenic tissue or embryos in extraction buffer, were ground with a pestle in 1.5-ml Eppendorf tubes. RNAs were digested with RQ1 DNase (Promega, Madison, WI, USA) following the manufacturer’s instructions, and then purified using the Amicon® Ultra columns (Merck Millipore, Darmstadt, Germany). The RNA concentration and quality were determined using a ND-1000 Spectrophotometer (NanoDrop Technologies Inc., USA). RNA was prepared from at least two biological replicates. cDNA synthesis was performed using 1 μg of total RNA. For quantitative RT-PCR, RT reactions were performed using 200 ng random primers with SuperScript™III reverse transcriptase (Invitrogen Corporation, Carlsbad, CA, USA) according to the manufacturer’s instructions.

### Phylogenetic analysis

The conserved C-terminal region of the GRAS proteins, plus as much of the N-terminal region as the shortest protein sequence allowed, were used for the phylogenetic analysis as previously described [16,17]. The polypeptides were aligned with Clustal Omega and subsequently analyzed with programs from the PHYLIP package (Phylogeny Interference Package, version 3.67, Department of Genetics, University of Washington, Seattle, WA, USA) at the Mobyle portal (http://mobyle.pasteur.fr/) [113]. A bootstrap analysis was performed with SEQBOOT and generated 1000 replicates that yielded a set of distance matrices with PROTDIST using the Dayhoff PAM matrix algorithm. A set of un-rooted trees was generated by the neighbor-joining method using NEIGHBOR, and a consensus tree was obtained with CONSENSE. A putative SCL encoded by a *Physcomitrella patens* EST [114] was used as the outgroup. The tree was drawn using TreeDyn at the Phylogenie portal (http://www.phylogeny.fr/version2_cgi/one_task.cgi?task_type=treedyn) [115].

### Pattern of protein intrinsic disorder

Natively disordered regions of GRAS proteins were predicted using both the Protein Disorder Prediction System server (http://prdos.hgc.jp/cgi-bin/top.cgi) [116] and the IUPRED method (http://toolkit.tuebingen.mpg.de/quick2_d) [117].

### Quantitative RT-PCR (qRT-PCR)

RNA extraction, quantification and cDNA synthesis were previously described [16]. Primer design, efficiency analyses, and polymerase chain reactions were carried out as previously described [16]. An 18S rRNA gene (*Ri18S*) was used as a control [16]. Pine *GRAS* specific primers were designed based on *P. radiata* sequences obtained in our laboratory (see list of primers below). Expression ratios were obtained from the equation 2^^-ΔΔCT^ (Applied Biosystems, Technical Bulletin #2, P/N4303859B). Results are expressed as mean values ± standard error from at least three biological replicates.

Primers for amplification of *P. radiata GRAS* genes are as follows: PrSCR F: TGTCACGGGCTCAGACACAA, PrSCR R: GGAAGGAACCTCCATGGCTC, PrSCL1 F: TCAATGTCTGGCAAATCGTCC, PrSCL1 R: GCGCCCAGTCTCTTCAATTCT, PrSCL2 F: TCAGTGGCGTATTGTGATGGA, PrSCL2 R: AGAGAGAAACCCCGACGATTC, PrSHR F: GAACCAGTGCAAGGAGCATTG, PrSHR R: AAATCCTGCCTCCTTGAGCCT, PrSCL5 F: TCTAAACCCTTGCGCAGTAGC, PrSCL5 R: CCCATGTGCTGCAAGCCTA, PrSCL6 F:ACCCAGAGAATGAGAAAGGCC, PrSCL6 R: TCTTTCTTCAGACCCCATCCA, PrSCL7 F: CCTTGCCCGAGACATAGTGAA, PrSCL7 R: AAGCCTGCCATGGTCATTCTA, PrSCL8 F: GCTGGCTTTACCGTATACCCC, PrSCL8 R: CCCCCTTTTCTGCCTTCAGT, PrSCL10 F: AGAATGGAGTTTGGAGGCGTT, PrSCL10 R: GCACCCTGGAGCTATCTGCA, PrSCL12 F: ACCTCCTCTGCCTCTTTCGTT, PrSCL12 R: ACGGCGTCCATGTTGATGT, PrSCL13 F: CCTTGAGGCTGTCCACATGA, PrSCL13 R: TGCCTTCTATAGGCCGCTTCT, PrSCL14 F: GGCCAATCACAATGGACCTG, PrSCL14 R: TTGGAAGCACATTGCATGCT, PrSCL16 F: TTATGAGTAGTGCGCCCGG and PrSCL16 R: GTTGCTTACGCTGCATTCCTC.

### *In situ* hybridization

For analysis, 1-cm basal segments of hypocotyl and epicotyl cuttings from 90-day-old seedlings treated with 10 μM IBA for 24 h, as well as corresponding controls were used. The basal 1 cm of the cuttings were embedded and frozen in Jung Tissue Freezing medium (Leyca Microsystems, Heildelberg, Germany) in dry ice. The basal 5 mm of samples were cut into 10-μm transverse sections and collected on 3-aminopropyl-triethoxisilan glass slides. Cryostat sections were dried on a hot plate at 40°C and fixed in 3:1 (v/v) ethanol:glacial acetic acid for 10 min followed by 5 min in 70% ethanol. To generate *PrSHR* specific probes, a 350 bp fragment corresponding to the 3′-untranslated region of *PrSHR* [lacking the poly(A) tail] was cloned into the PCR® II vector (Invitrogen Corporation, Carlsbad, CA, USA) and amplified. The PCR fragment, flanked by the SP6 and T7 promoters, was used as the template for synthesis of both sense and antisense DIG-labeled probes, with T7 or SP6 polymerase, respectively, according to the manufacturer’s instructions (DIG RNA Labelling Kit SP6/T7, Roche Biochemicals, Indianapolis, IN, USA). The probes were partially hydrolyzed to an average length of 200 nucleotides by alkali treatment. The *in situ* hybridization was performed as described by Sánchez et al. [118]. Sections were treated with Proteinase K at 1 μg · mL^−1^ for 30 min at 37°C. After Proteinase K pre-treatment, sections were incubated overnight at 43°C with the RNA probes in a hybridization solution containing 40% deionized formamide. After washing four times in 2XSSC (1XSSC 150 mM sodium chloride and 15 mM sodium citrate) at 37°C, slides were treated with RNase A (5 μg · mL^−1^) at 37°C for 30 min, and washed twice with 0.1XSSC at 37°C. The hybridization signal was detected by using the DIG Nucleic Acid Detection Kit (Roche Biochemicals, Indianapolis, IN, USA) for 12 h in the dark following the manufacturer’s instructions. Sections were dehydrated through an ethanol series (v/v) (50% and 70% for 30 s each, and 99% for 1 min twice), air dried and mounted in Eukitt (O. Kindler, GmbH & Co., Freiburg, Germany). Photographs were taken with an Olympus digital camera on a Nikon microscope under bright-field illumination.

### Auxin immunolocalization

The 1-cm basal segments of hypocotyls or epicotyls from 21- and 90-day-old seedlings treated with 10 μM IBA for 24 h, and the corresponding controls, were excised and fixed in 4% paraformaldehyde in phosphate-buffered saline (PBS) at 4°C overnight. The 1-cm basal segments of hypocotyls from 21-day-old seedlings treated with 10 μM IBA + 10 μM NPA for 24 h and the corresponding controls were also excised and fixed. The segments were then washed three times, 10 min each, in PBS, and post-fixed in 0.1% paraformaldehyde in PBS at 4°C until use. Cryosections were incubated with 5% bovine serum albumin (BSA) in PBS for 5 min and then, with an anti-IAA mouse monoclonal antibody (Sigma-Aldrich, St. Louis, MO, USA) diluted 1/100 in 1% BSA overnight at 4°C in a wet chamber. After washing in 1% BSA five times, 5 min each, the signal was revealed with ALEXA 568 conjugated anti-mouse antibodies (Molecular Probes, Eugene, OR, USA), diluted 1:25 in PBS for 45 min in the dark. The sections were counterstained with DAPI after washing in PBS, mounted in Mowiol and observed in a Leica SP5 confocal microscope. Confocal optical sections were collected using LAS AF confocal scanning. Controls were performed by replacing the first antibody with PBS.

### Availability of supporting data

The data sets supporting the results of this article are included within the article and its additional files. The nucleotide sequences of *P. radiata* GRAS genes have been deposited in the GenBank database under the following accession numbers: PrSCR, KM264388; PrSCL2, KM264389; PrSCL3, KM264390; PrSCL4, KM264391; PrSCL5, KP244290; PrSCL6, KM264392; PrSCL7, KM264393; PrSCL8, KP244291; PrSCL9, KM264394; PrSCL10, KM264395; PrSCL11, KM264396; PrSCL12, KM264397; PrSCL13, KM264398; PrSCL14, KM264399; PrSCL16, KP244292; and PrSCL18, KM264400.

## Additional files

Additional file 1:
***GRAS*** **genes of** ***Pinus radiata, Pinus taeda, Pinus pinaster*** **and** ***Picea abies.*** Genes were grouped according to the different GRAS families. Additional file 2:
**Phylogenetic tree of GRAS proteins from conifer and angiosperm species.** Accession no. in parentheses; accession no. or gene references of conifer sequences from Figure 2. *Arabidopsis thaliana* SCR (U62798), *A. thaliana* SHR (AF233752), *A. thaliana* SCL1 (AF210731), *A. thaliana* SCL3 (NM_103925), *A. thaliana* SCL4 (NM_126075), *A. thaliana* SCL5 (NM_103942), *A. thaliana* SCL6 (NM_116232), *A. thaliana* SCL7 (NM_114925), *A. thaliana* SCL8 (NM_1246), *A. thaliana* SCL9 (NM_129321), *A. thaliana* GAI (Y15193), *A. thaliana* GRS (CAA75493), *A. thaliana* RGL1 (AY048749), *A. thaliana* RGL3 (AL391150), *A. thaliana* SCL11 (NM_125336), *A. thaliana* SCL13 (AF419570), *A. thaliana* SCL14 (NM_100627), *A. thaliana* SCL18 (NM_104434), *A. thaliana* SCL19 (AC009895), *A. thaliana* SCL21 (AF210732), *A. thaliana* SCL22 (NM_115927), *A. thaliana* SCL23 (NM_123557), *A. thaliana* PAT1 (AF153443), *A. thaliana* SCL26 (NM_116894), *A. thaliana* SCL27 (NM_130079), *A. thaliana* SCL28 (NM_104988), *A. thaliana* SCL29 (NM_112237), *A. thaliana* SCL30 (NM_114527), *A. thaliana* SCL31 (NM_100626), *A. thaliana* SCL32 (NM_114855), *Brasica napus* SCL1 (AY664405), *Castanea sativa* SCL1 (DQ683579), *Cucumis sativus* SCR (AJ870306), *Lilium longiflorum* SCL (AB106274), *Lycopersicom esculentum* LS (AF098674), *Oryza sativa* MOCI (AY242058), *O. sativa* SHR1 (XM_468819), *O. sativa* SHR2 (NP_911918), *O. sativa* GAI (NM_001057567), *O. sativa* CIGR1 (AY062209), *O. sativa* CIGR2 (AY062210), *O. sativa* SCR (BAD22576), *O. sativa* SCR1 (NP_001065617), *O. sativa* SCR2 (NP_001066027), *Petunia hybrida* HAM (AF481952), *Pisum sativum* SCR (AB048713) and *Zea mays* SCR (AF263457). *Physcomitrella patens* PpSCL (BJ976460) was used as the outgroup. Branches with bootstrap values lower than 500 were collapsed. SCL, SCARECROW-LIKE; SCR, SCARECROW; SHR, SHORT-ROOT. Additional file 3:
**Alignment of pine GRAS amino acid-deduced sequence from the C-terminal region in each GRAS subfamily.** Pine members from each subfamily and representative members from other species were aligned. Conserved amino acids are displayed in dark grey. Similar amino acids are displayed in light grey. Specific conserved domains are underlined. Specific pairs of conserved residues are indicated with asterisks. Additional file 4:
**Deduced amino acid sequence of GRAS proteins from pine.** Basic and acidic amino acids, as well as stretches of different amino acids are highlighted in the N-terminal region. Additional file 5:
**Prediction of intrinsic disorder for the N-terminal region of pine GRAS proteins in each GRAS subfamily.** Pine members from each subfamily and representative members from other species were compiled using Clustal. Predicted disordered domains are outlined. Conserved amino acids are displayed in dark grey. Similar amino acids are displayed in light grey. Additional file 6:
**Endogenous distribution of indole-3-acetic acid (IAA) in hypocotyl cuttings from 21-day-old seedlings, and hypocotyls or epicotyls cuttings from 90-day-old seedlings.** Transverse sections of the base of hypocotyls (A, B) from 21-day-old seedlings, and hypocotyls (C, D) or epicotyls (E, F) from 90-day-old seedlings after 24 h of culture in the presence of 10 μM indole-3-butyric acid in the presence (A, C, E) or absence (B, D, F) of an antibody raised against IAA.

## Abbreviations

IAA: Indol-3-acetic acid
IBA: Indol-3-butyric acid
NPA: 1-N-naphthylphthalamic acid
qRT-PCR: Quantitative reverse transcription-PCR
SCL: SCARECROW-LIKE
SCR: SCARECROW
SHR: SHORT-ROOT
