# Supplementary figures and images for: The GRAS gene family in pine: transcript expression patterns associated with the maturation-related decline of competence to form adventitious roots
Source: BMC Plant Biol. 2014 Dec 30;14:354. doi: 10.1186/s12870-014-0354-8 (PMC4302573; doi:10.1186/s12870-014-0354-8)

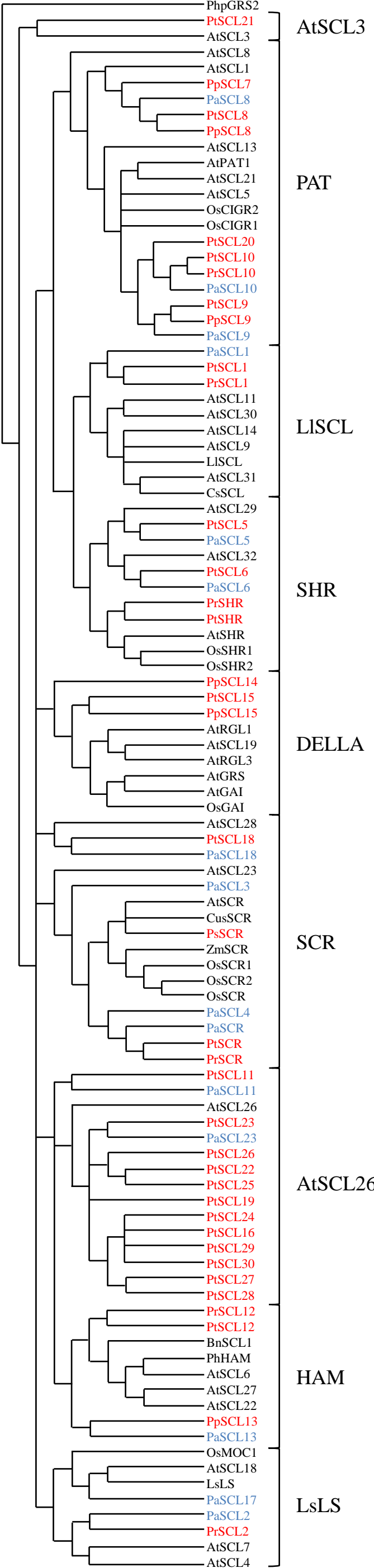

Supplement: Additional file 2: — Phylogenetic tree of GRAS proteins from conifer and angiosperm species. Accession no. in parentheses; accession no. or gene references of conifer sequences from Figure 2. Arabidopsis thaliana SCR (U62798), A. thaliana SHR (AF233752), A. thaliana SCL1 (AF210731), A. thaliana SCL3 (NM_103925), A. thaliana SCL4 (NM_126075), A. thaliana SCL5 (NM_103942), A. thaliana SCL6 (NM_116232), A. thaliana SCL7 (NM_114925), A. thaliana SCL8 (NM_1246), A. thaliana SCL9 (NM_129321), A. thaliana GAI (Y15193), A. thaliana GRS (CAA75493), A. thaliana RGL1 (AY048749), A. thaliana RGL3 (AL391150), A. thaliana SCL11 (NM_125336), A. thaliana SCL13 (AF419570), A. thaliana SCL14 (NM_100627), A. thaliana SCL18 (NM_104434), A. thaliana SCL19 (AC009895), A. thaliana SCL21 (AF210732), A. thaliana SCL22 (NM_115927), A. thaliana SCL23 (NM_123557), A. thaliana PAT1 (AF153443), A. thaliana SCL26 (NM_116894), A. thaliana SCL27 (NM_130079), A. thaliana SCL28 (NM_104988), A. thaliana SCL29 (NM_112237), A. thaliana SCL30 (NM_114527), A. thaliana SCL31 (NM_100626), A. thaliana SCL32 (NM_114855), Brasica napus SCL1 (AY664405), Castanea sativa SCL1 (DQ683579), Cucumis sativus SCR (AJ870306), Lilium longiflorum SCL (AB106274), Lycopersicom esculentum LS (AF098674), Oryza sativa MOCI (AY242058), O. sativa SHR1 (XM_468819), O. sativa SHR2 (NP_911918), O. sativa GAI (NM_001057567), O. sativa CIGR1 (AY062209), O. sativa CIGR2 (AY062210), O. sativa SCR (BAD22576), O. sativa SCR1 (NP_001065617), O. sativa SCR2 (NP_001066027), Petunia hybrida HAM (AF481952), Pisum sativum SCR (AB048713) and Zea mays SCR (AF263457). Physcomitrella patens PpSCL (BJ976460) was used as the outgroup. Branches with bootstrap values lower than 500 were collapsed. SCL, SCARECROW-LIKE; SCR, SCARECROW; SHR, SHORT-ROOT. [file 12870_2014_354_MOESM2_ESM.pdf]

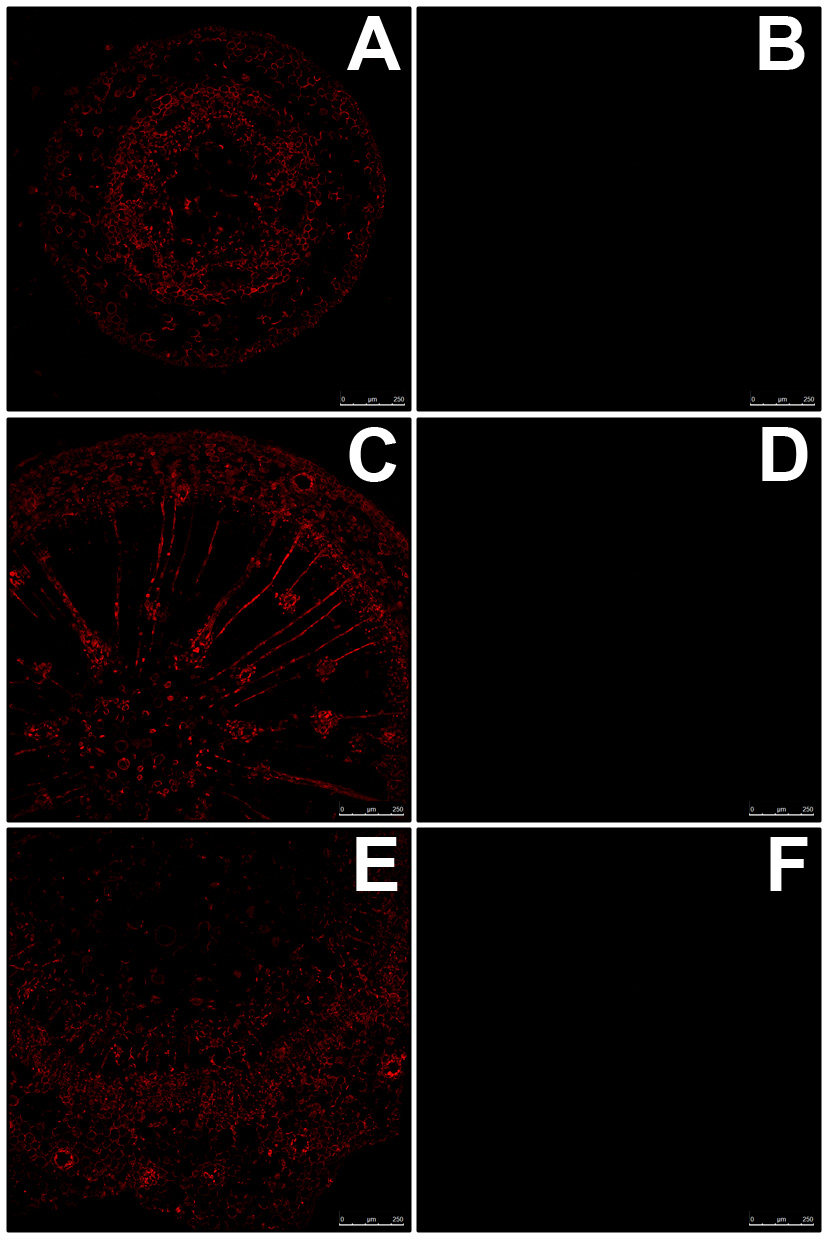

Supplement: Additional file 6: — Endogenous distribution of indole-3-acetic acid (IAA) in hypocotyl cuttings from 21-day-old seedlings, and hypocotyls or epicotyls cuttings from 90-day-old seedlings. Transverse sections of the base of hypocotyls (A, B) from 21-day-old seedlings, and hypocotyls (C, D) or epicotyls (E, F) from 90-day-old seedlings after 24 h of culture in the presence of 10 μM indole-3-butyric acid in the presence (A, C, E) or absence (B, D, F) of an antibody raised against IAA. [file 12870_2014_354_MOESM6_ESM.jpeg]
