# Supplementary material for: The GRAS gene family in pine: transcript expression patterns associated with the maturation-related decline of competence to form adventitious roots
Source: BMC Plant Biol. 2014 Dec 30;14:354. doi: 10.1186/s12870-014-0354-8 (PMC4302573; doi:10.1186/s12870-014-0354-8)
Supplement: Additional file 3: — Alignment of pine GRAS amino acid-deduced sequence from the C-terminal region in each GRAS subfamily. Pine members from each subfamily and representative members from other species were aligned. Conserved amino acids are displayed in dark grey. Similar amino acids are displayed in light grey. Specific conserved domains are underlined. Specific pairs of conserved residues are indicated with asterisks. [file 12870_2014_354_MOESM3_ESM.pdf]

SCR

```
         _________________________LHRI___________________________                          ________________
         * *  ***  *  *       *   *  *             **   *  *   *                                    *
AtSCR    LHLLTLLLQCAEAVSADNLEEANKLLLEISQLSTPYGTSAQRVAAYFSEAMSARLLNSCLGIYAALP-SRWMPQTHSLKMVSAFQVFNGISPLVKFSH
PsSCR    LHLLTLLLQCAEAVSAENLEQANKMLLEISQLSTPFGTSAQRVAAYFSEAISARLVSSCLGIYATLP-VSSHTP-HNQKVASAFQVFNGISPFVKFSH
OsSCR    LHLLTLLLQCAESVNADNLDEAHRALLEIAELATPFGTSTQRVAAYFAEAMSARLVSSCLGLYAPLPNPSPAAARLHGRVAAAFQVFNGISPFVKFSH
ZmSCR    LHLLTLLLQCAEAVNADNLDDAHQTLLEIAELATPFGTSTQRVAAYFAEAMSARLVSSCLGLYAPLPPGSPAAARLHGRVAAAFQVFNGISPFVKFSH
PrSCR    LHLLALLLQCAEAVSADNFEEANTILPQITELSTPYGNSVQRVAAYFAEAMSARLVSSCIGMYSPLP---PIHMSQSQKIVNAFQVFNGISPFVKFSH
PaSCL3   LHLLSLLLQCAEAISMDKIQQAYSILPQLSEFSTPYGNPLQRVAAYFAEGMAARLHNSYLGLCCPLEISRPQLVSINHNFLTAFQTFNSICPFVKFSH
PaSCL4   LHLLALLLQCAEAVSEDNHEEANSILPHIYELSSPYGSSVHRVAAYFAEALSCRLFSSYIGIYSPI-----MNESRSQSIVSAFQLYNGISPFVKFSH
PtSCL18  LELANLLIACAEAVTSKNVQLVNRLLSRLGELASPESPPMHRVAAYFTEGLTLRVTKLWPHIFQPI---VGDLDLCDEDLITGFQLLNHTTPIPKF--

         ______VHIID____________________________________________     ________________LHRII____________
              *             *  *       *   *                         *      *      *        **     *
AtSCR    F--TANQAIQEAFEKEDSVHIIDLDIMQGLQWPGLFHILASRPGGPPHVRLTGLGTSMEALQATGKRLSDFTDKLGLPFEFCPLAEKVGNLDT--ERL
PsSCR    F--TANQAIQEAFEREERVHIIDLDIMQGLQWPGLFHILASRPGGPPYVRLTGLGTSMETLEATGKRLSDFANKLGLPFEFFPVAEKVGNIDV--EKL
OsSCR    F--TANQAIQEAFEREERVHIIDLDIMQGLQWPGLFHILASRPGGPPRVRLTGLGASMEALEATGKRLSDFADTLGLPFEFCPVADKAGNLDP--EKL
ZmSCR    F--TANQAIQEAFEREERVHIIDLDIMQGLQWPGLFHILASRPGGPPRVRLTGLGASMEALEATGKRLSDFADTLGLPFEFCAVAEKAGNVDP--EKL
PrSCR    F--TANQAIQEAFEREQRVHIIDLDIMQGLQWPGLFHILASRPGGPPHVRITGLGTSLEALEATGKRLSDFAHTLNLPFEFHPVADKVGKLDP--ERL
PaSCL3   F--TSNQAILEAFNGESQVHIIDFDIMQGLQWPALFHILAARPGGCPHVRITGLGTSMEALEATGKRLSSFAHTLKLPFEFHPVAEKLGQLDP--AVL
PaSCL4   F--TANQAIQQVFQGENSVHIIDLDIMQGLQWPGLFHILASRPGSPPFVRITGLGTSMEALEATGKRLSNFAETLGIPFEFHAVSDKVGNINENLERL
PtSCL18  FQYTANEVILKAFEDKDRVHIIDFDI-QGLQWPALFHSLATRPRPPSHVRITGIGESKEDLQDTGDRLAGFAEALNLPFEFHAVVDKL--EDVRLWML

                ____________________________________________PFYRE _________________________________________
          *     ***                            *                *          *                 *   *
AtSCR    NVRKREAVAVH-WLQ-H-SLYDVTGSDAHTLWLLQRLAPKVVTVVEQDLSH-AGSFLGRFVEAIHYYSALFDSLGASYGEESEERHVVEQQLLSKEIR
PsSCR    NVSKSEAVAVH-WLQ-H-SLYDVTGSDTNTLWLLQRLAPKVVTVVEQDLSN-AGSFLGRFVEAIHYYSALFDSLGSSYGEESEERHVVEQQLLSREIR
OsSCR    GVTRREAVAVH-WLR-H-SLYDVTGSDSNTLWLIQRLAPKVVTMVEQDLSH-SGSFLARFVEAIHYYSALFDSLDASYSEDSPERHVVEQQLLSREIR
ZmSCR    GVTRREAVAVH-WLH-H-SLYDVTGSDSNTLWLIQRLAPKVVTMVEQDLSH-SGSFLARFVEAIHYYSALFDSLDASYGEDSPERHVVEQQLLSREIR
PrSCR    KVNRGDALAVH-WLH-H-SLYDVTGSDTNTLRLLQRLSPKVITVVEQDLSH-GGSFLSRFVEAIHYYSALFDSLGASYPEDSHDRHLVEQQLLSREIK
PaSCL3   RVRSGDALAVH-WLH-H-SLYDVTGSDTCTLRLLQRLNPKVITMVEQDLNT-VGPFLNRFVEALHYYSAMFDALGACFNEDNMERHTVEQQLLSHEIR
PaSCL4   KVRKGDALAVH-WLQ-H-SLYDVTGSDISTLCLLRRLCPKVITMVEQDLSH-VGSFLNRFVEAIHYYSAVFDSLGAAYPEDSHERHVVEQQLLSCEIK
PtSCL18  HVKEKETVAVNCMLQLHKTLYDNGAALRGILGLLQSTKPAVVVVVEQEANHNDPIFEGRFLQALQYYSAIFDSIDASLPSDSPARLKMEQ-YFAREIR

            _________________________________SAW_______________________________________
                  *   *    *       *                                       *        ***
AtSCR    NVLAVGGPSRSGEVKFESWREKMQQCGFKGISLAG-NAATQATLLLGMFPSDGYTLVDDNGTLKLGWKDLSLLTASAWTPRS------------
PsSCR    NVLAVGGPSRSGEIKFHNWREKLQQCGFRGVSLAG-NAATQASLLLGMFPSEGYTLVEDNGILKLGWKDLCLLTASAWRPPYHTNTIIPHHN--
OsSCR    NVLAVGGPARTGDVKFGSWREKLAQSGFRVSSLAG-SAAAQAVLLLGMFPSDGYTLIEENGALKLGWKDLCLLTASAWRPIQASGR--------
ZmSCR    NVLAVGGPARTGDVKFGSWREKLAQSGFRAASLAG-SAAAQASLLLGMFPSDGYTLVEENGALKLGWKDLCLLTASAWRPIQVPPCR-------
PrSCR    NILAVGGPARTGEIKFDNWRDQLKQTGFKPISLAG-NAATQATLLLGMFPCQGYTLMEENGTLKLGWKGLCLLTASAWRPAHHGLIY-------
PaSCL3   NILAVGGPARTGETKFQNWRTHLQSCGFRQISMRG-NASVQAELLLNMFCCEGYSLVEEGGILKFGWKDLSLYIASAWTSWTS-----------
PaSCL4   NILAVGGPARTGELRFHNWRDQLQQAGFSKLSLAG-NAAAQATLLLGMFPCKGYTLVEESGTLRIGWKDICLLTASAWRPAHHAL---------
PtSCL18  NIIACEGPERIERHEKFEHWKQLMEEGGFTAVPLGEREHCQARILLDMFPCKNYIVKLEDGRLTLDWLHQPLYTASAWAPQEGGNGGASTSSPA
```

SHR

```
                    _______________________LHRI____________________________                            ___________________________
                    *  ***  *  *       *   *  *               **   *  *   *                                     *         *
AtSHR            AKWADSVLLEAARAFSDKDTARAQQILWTLNELSSPYGDTEQK--LASYFLQALFNRMTGSGERCYRTMVTAATEKTCSFESTRKTVLKFQEVSPWATFGHVAANGAILE--
OsSHR1           GRWASQLLLECARSVAARDSQRVQQLMWMLNELASPYGDVEQK--LASYFLQGLFARLTASGPRTLRTLAAAS--DRNTSFDSTRRTALRFQELSPWSSFGHVAANGAILESF
PrSHR            RRWASNLLLECARAIAENEKSRTQHLLWMLNELSSPYGDCEQK--LASYFLQAFFCKITDTGPRCYTTLCSAA--EKTYSFDSTRKMILKFQESSPWTTFGHVAANGAILES-
PtSCL5           SRWAEQLLNPCAVAIANNNIVRIQHLIWVLHDLASISGDANHR--LAAHGLRAFTAKIMHPGAS---LPCSEF---LSADPKLFHKALXQFHEVSPWYQLLYTIANKYLLEAF
PtSCL6           SCMEQLLLHCSS-ALENNDVTLAQQIMWVLNNIASAEGDPNQRVTSCFLRALIARAARMSNSNMLRAMAATTANAHVGDTKRLSVIELAGFVDLTPWHRFGFSSSNGAIAE--

                 __________VHIID______________________________________            ______________________LHRII_____________________
                               *  *       *   *                                   **  *   *     ** *     **          *    *  * *
AtSHR            ------AVDGEAKIHIVDISSTFCTQWPTLLEALATRS-DDTPHLRLTTVVVANKFVNDQTASHRMMKEIGNRMEKFARLMGVPFKFNIIHHVG-----DLSEFDLNELDVK-
OsSHR1           LEVAAAASSETQRFHILDLSNTFCTQWPTLLEALATRSADETPHLSITTVVSAAP-SAPTAAVQRVMREIGQRMEKFARLMGVPFRFRAVHHSG-----DLAELDLDALDLRE
PrSHR            -------FEGEMKLHIVDLSNTFCTQWPTLLEALATRS-DDTPHLRLTTVVTNK-----EATAMKVMKEIGQRMEKFARLMGVPFEFSVIHQH------HLHKLNVGALKIR-
PtSCL5           ED------QNSKALHVIDIGVSHGIQWPTFMEALARRPAGPPSLLRLTIIAD-TLGAPFSAGPP--SNDFPNRLKRYAKTLNLDMEVNVIAQPLD----SLTREALGVKEDEA
PtSCL6           ------AVEGYSVVHILDFGIAHCMQWPTLIDSLSKRPDGPP-YIRLTVSCARPPVPPL---LNMSYEELGTRLSNFARSRNVPFRFRAIPPDPSEEFLVLDQLEPSILDIRE

                         ________________________________________________PFYRE____________________________________________________
                         ***                                      *                                *          *                  *
AtSHR              --PDEVLAINCVGAMHGIASRGSP-----------RDAVISSFRRLRPRIVTVVEEEADLVGEEE---------GGFDDEFLRGFGECLRWFRVCFESWEESFP-RTSNER
OsSHR1             GGATTALAVNCVNSLRGVVPGRARR----------RDAFAASLRRLDPRVVTVVEEEADLVASDPDASSATEEGGDTEAAFLKVFGEGLRFFSAYMDSLEESFP-KTSNER
PrSHR              --PDEALAINCIHSLQRVTKNG-------------RDSILSTFYSMNPKIVTVVEDEVDL----------------THEDFGDCFSECLRFFSLFFDSLEESFS-RTSNER
PtSCL5             LAICGQFRLHQLLGE-GLDKNEGKD--NQLTSVSPRDKFIKFIKDLNPLVFFLSDNDIDHSS----------------PDFIERFKNSVDHLWRFLDSTSVCFKGRECEER
PtSCL6             ---GEALVINCQFRLHYIPDETADQQSSCSSSSCQRDEFLRIIRSFEPTIMTLVDEDSDL----------------TSTSLVSRLKSAFNYLWIPFDALNTFLP-RDSKQR

                   __________    _____________________________________SAW_____________________________________________
                      *                    *      *       *                                               *        ***
AtSHR              LMLERAAGRAIVDLVACEPSDSTERRETARKWSRRMRNSGFGAVGYSDEVADDVRALLRRYKEGVWSMVQCP-------DAAGIFLCWRDQPVVWASAWRPT-----
OsSHR1             LALERGAGRAIVDLVSCPASESMERRETAASWARRMRSAGFSPVAFSEDVADDVRSLLRRYREG-WSMREAG--TDDSAAGAGVFLAWKEQPLVWASAWRP------
PrSHR              LMLERTSARSIVNILACEDSEVYERREKGAQWAWRLKEAGFIHAAFSDDVVDDVRALLKRYKEG-WGHCSNS---------DGLFLTWKEQCAIWASAWKPCL----
PtSCL5             RMVEGEAAMGLVNNVAYDGASRVERNENHLNWANRVREEGFVCEIPSDKIVDCARAMLRKHDSN-WEMRLEDG---------CISLCWKSHPVTFCSLWKPCFNEPT
PtSCL6             QQYESDVGYKIENIIAFEGHQRIERLESKAKWTQRMRKACFQSISFSEDTVAEVKAMLDEHAAG-WGLKKEEDD---------LLLTWKGHNVVFATAWVPSHL---
```

LlSCL

```
                         LHRI
AtSCL9   VDLRSLLIHCAQAVAADDRRCAGQLLKQIRLHSTPFGDGNQRLAHCFANGLEARLAG-TGSQI---YKGIVSKPRS-AAAVLKAHQLFLACCPFRKLSYFITNKTIRDLVGNSQ
AtSCL11  VDLRSLLTQCAQAVASFDQRRATDKLKEIRAHSSSNGDGTQRLAFYFAEALEARITGNISPPVSN---PFPSSTTS-MVDILKAYKLFVHTCPIYVTDYFAANKSIYELAMKAT
AtSCL14  ADLRTLLVLCAQAVSVDDRRTANEMLRQIREHSSPLGNGSERLAHYFANSLEARLAG-TGTQI---YTALSSKKTS-AADMLKAYQTYMSVCPFKKAAIIFANHSMMRFTANAN
AtSCL30  VDMRNLLMQCAQAVASFDQRRAFEKLKEIREHSSRHGDATQRLGYHFAEALEARITGTMTTPIS-----ATSSRTS-MVDILKAYKGFVQACPTLIMCYFTANRTINELASKAT
AtSCL31  VDFRTLLTLCAQSVSAGDKITADDLLRQIRKQCSPVGDASQRLAHFFANALEARLEGSTGTMIQSYYDSISSKKRT-AAQILKSYSVFLSASPFMTLIYFFSNKMILDAAKDAS
CsSCL    VDLRSLLILCAQAVSTGDGRTANELLKQIRQHSTPFGDGSQRLAHFFANGLEARLAG-TSVGTQMFYT--SNRALS-TLEKLKAYQVHLSACPFKRIAYSFSNKMIFHAAERET
LlSCL    VDLRTLLIHCAQTVAIDDRRSANDLLKQIRQHASPFGDGMQRLAHYFADGLEARLAG-MGSEK---YHSFVAKPVS-ATDILKAYGLYMSACPFKKVSFYFSTQMILDTTEKAS
PrSCL1   VDLRSLLLICAQSVAADDTRGANETLKQIRQHASAYGDGSQRLANYFADGIAARLSG-SGGRL---FTMISSGALSSAAEILKAYQLLLVATPFKKISHFMTYQTVLNVAEGET

                   VHIID                                         LHRII
AtSCL9   RVHVIDFGILYGFQWPTLIHRFSMY--G-SPKVRITGIEFPQPGFRPAQRVEETGQRLAAYAKLFGVPFEYKAIA-KKWDAIQLEDLDIDRDEITVVNCLYRAENLHDES-VKV
AtSCL11  KLHIVDFGVLYGFQWPCLLRALSKRPGG-PPMLRVTGIELPQAGFRPSDRVEETGRRLKRFCDQFNVPFEFNFIA-KKWETITLDELMINPGETTVVNCIHRLQYTPDET-VSL
AtSCL14  TIHIIDFGISYGFQWPALIHRLSLSRPGGSPKLRITGIELPQRGFRPAEGVQETGHRLARYCQRHNVPFEYNAIA-QKWETIQVEDLKLRQGEYVVVNSLFRFRNLLDET-VLV
AtSCL30  TLHIIDFGILYGFQWPCLIQALSKRDIG-PPLLRVTGIELPQSGFRPSERVEETGRRLKRFCDKFNVPFEYSFIA-KNWENITLDDLVINSGETTVVNCILRLQYTPDET-VSL
AtSCL31  VLHIVDFGILYGFQWPMFIQHLSKSNPG-LRKLRITGIEIPQHGLRPTERIQDTGRRLTEYCKRFGVPFEYNAIASKNWETIKMEEFKIRPNEVLAVNAVLRFKNLRDVIPGEE
CsSCL    TLHIVDFGIQYGFQWPLLIQFLSKRPEG-APKLRITGIDLPQPGFRPAECIEETGRRLEKYCNRFNVPFEYNAIASQKWETIRIEELKIERNEVLAVNCAFRMKNLLDET-VEG
LlSCL    KIHIVDFGIYFGFQWPSFLQRLSKRPGG-PPKLRITGIDLPQPGFRPAERIEQTGRRIAEYARSFNVPFEYQGIA-AKFETIKIEDLRIAEDEMVVVNCSFSLKNLADET-VAE
PrSCL1   RLHIVDFGILYGFQWPSLIQCLANRPGG-PPMLRITGIEFPQPGFRPAERIEETGRRLEDYAKSFGVPFEYQAIA-TKWENLDVEELGLRSDEVLVVNCLGRLRNLLDET-VVQ

                         PFYRE
AtSCL9   ESCRDTVLNLIGKINPDLFVFGIVNGAYNAPFFVTRFREALFHFSSIFDMLETIVPREDEER--MFLEMEVFGREALNVIACEGWERVERPETYKQWHVRAMRSGLVQVPFDPS
AtSCL11  DSPRDTVLKLFRDINPDLFVFAEINGMYNSPFFMTRFREALFHYSSLFDMFDTTIHAEDEYKNRSLLERELLVRDAMSVISCEGAERFARPETYKQWRVRILRAGFKPATISKQ
AtSCL14  NSPRDAVLKLIRKINPNVFIPAILSGNYNAPFFVTRFREALFHYSAVFDMCDSKLAREDEMR--LMYEKEFYGREIVNVVACEGTERVERPETYKQWQARLIRAGFRQLPLEKE
AtSCL30  NSPRDTALKLFRDINPDLFVFAEINGTYNSPFFLTRFREALFHCSSLFDMYETTLSEDDNCR--TLVERELIIRDAMSVIACEGSERFARPETYKQWQVRILRAGFRPAKLSKQ
AtSCL31  DCPRDGFLKLIRDMNPNVFLSSTVNGSFNAPFFTTRFKEALFHYSALFDLFGATLSKENPER--IHFEGEFYGREVMNVIACEGVDRVERPETYKQWQVRMIRAGFKQKPVEAE
CsSCL    TSPRDAVLNLIRRMKPDIFINSIVNGSYNAPFFLTRFREALFHFSALYDVFDVTIPRDNPQR--VMFEREFYGREAMNVIANEGLERVERPETYKQSQFRISRAGFKQLPLNQE
LlSCL    DCPRTRVLSMIRKLNPALFTLGVVNGSYNAPFFVTRFREALFHFSALFDMLEMNTPRKDEQR--LLIEQNIFGRDAMNVIACEGTERVERPETYKQWQVRNFRAGFTQLPLDRD
PrSCL1   DSPRNIVLNKIRSMNPRVFIQGVVNGAYNASFFITRFREALFHYSALFDALETTVPRDNQQR--FLIEKEIFGREILNVVACEGSERLERPETYKQWQGRTQRAGFVQLPLDRS

               SAW
AtSCL9   IMKTSLHKVHTF-YHKDFVIDQDNRWLLQGWKGRTVMALSVWKPESKA-------
AtSCL11  IMKEAKEIVRKR-YHRDFVIDSDNNWMLQGWKGRVIYAFSCWKPAEKFTNNNLNI
AtSCL14  LMQNLKLKIENG-YDKNFDVDQNGNWLLQGWKGRIVYASSLWVPSSS--------
AtSCL30  IVKDGKEIVKER-YHKDFVIDNDNHWMFQGWKGRVLYAVSCWKPAKK--------
AtSCL31  LVQLFREKMKKWGYHKDFVLDEDSNWFLQGWKGRILFSSSCWVPS----------
CsSCL    IMSLFRAKMKAW-YHKDFILDEDNHWMLQGWKGRIVYASSCWVPV----------
LlSCL    IVKKSKCKVKEL-YHKDFVVDEDGRWLLLGWKGRIIYALSAWTPNSR--------
PrSCL1   ILSKSRDKVKTF-YHKDFGVDEDGNWMLLGWKGRTIHALSTWRPST---------
```

PAT

```
         _________________________LHRI____________________________                          _____________________________
              ***   *  *         *   *  *                     **    *  *   *                           *            *
AtPAT1   RDLRADLVSCAKAMSENDL--MMAHSMMEKLRQMVSVSGEPIQRLGAYLLEGLVAQLASSGSSIYKALNRCPEPAS-TELLSYMHILYEVCPYFKFGYMSANGAIAEAMK
AtSCL1   ATPKQILISCARALSEGKL--EEALSMVNELRQIVSIQGDPSQRIAAYMVEGLAARMAASGKFIYRALK-CKEPPS-DERLAAMQVLFEVCPCFKFGFLAANGAILEAIK
AtSCL5   GDLKGVLYECAKAVENYDL--EMTDWLISQLQQMVSVSGEPVQRLGAYMLEGLVARLASSGSSIYKALR-CKDPTG-PELLTYMHILYEACPYFKFGYESANGAIAEAVK
OsCIGR2  GNLKELLIACARAVEEKNS--FAIDMMIPELRKIVSVSGEPLERLGAYMVEGLVARLASSGISIYKALK-CKEPKS-SDLLSYMHFLYEACPYFKFGYMSANGAIAEAVK
PtSCL7   SS-KQLLVQCATAISEGKE--EMASHIITKLRETVSIQGDPMERLAAYMVEGLAARIASSGNGIYKALN-CKAPPS-TDTLSAMQILFEVCPYFKFGCMVANGAICEAFK
PpSCL8   SSSRKLLVECATAISEGDK--DSALSIIKNLMQVISVYGDPMQRLTAYMVEGLVARLGPSAQSLYNTLK-WKETPIKSDILSATRLLYKVCPYMKFGCVAAISTILEALK
PpSCL9   GDVKELLIACAKAVSDSDNDSIKIDKLIAEIRQVVSISGDPMQRLGAYMVEGLVARMASSGAIIYKTLR-CKEPTS-PELLSYMQVLFEVCPYFKFGYMAANGAIAEAFK
PrSCL10  GDVKSLLIECAKAIADGRN----ADNLIAGLRQVVNIYGDPLHRLAAYMVEGLVARLHFSGGHIYKTLK-CKEPTS-SELLSYMHILYEVCPYFKFGYVAANGAIAEAFK
PtSCL20  GDIKCLLIECAKAIADNRN----ADKLIAELRQAVSIFGDPMQRLGAYMVEGLVARLQFSGGHIYKTLK-CKEPTS-AELLSYMQIFYKICPYFKFGYMSANAVIVEALN

         ____VHIID_______________________________             ________________LHRII________________     _______________
              *  *       *   *                                *   *    *  *       *      *       *      *     ***
AtPAT1   EENRVHIIDFQIGQGSQWVTLIQAFAARPGGPPRIRITGIDDMTSAYARGGGLSIVGNRLAKLAKQFNVPFEFNSVSVSVSEVKPKNLGVRPGEALAVNFAFVLHHMPDE
AtSCL1   GEEEVHIIDFDINQGNQYMTLIRSIAELPGKRPRLRLTGIDDPESVQRSIGGLRIIGLRLEQLAEDNGVSFKFKAMPSKTSIVSPSTLGCKPGETLIVNFAFQLHHMPDE
AtSCL5   NESFVHIIDFQISQGGQWVSLIRALGARPGGPPNVRITGIDDPRSSFARQGGLELVGQRLGKLAEMCGVPFEFHGAALCCTEVEIEKLGVRNGEALAVNFPLVLHHMPDE
OsCIGR2  GEDRIHIIDFHISQGAQWISLLQALAARPGGPPTVRITGIDDSVSAYARGGGLELVGRRLSHIASLCKVPFEFHPLAISGSKVEAAHLGVIPGEALAVNFTLELHHIPDE
PtSCL7   DEQKVHIIDFEIGQGSQYISLLKVLAERPGGPPHLRITAVDDSEDVRYIPGGLDNVGKRLEQYAESIGASLQFISIRRKVGDVQPWMLEINPDEVLAVNFAFQLHHMPDE
PpSCL8   EEEKVHIIDFEIGEGSQYFTLIDKLAARVGGPPKLRITAVDDPEPISRSVGDLYMLQEQLREIAVQRGVHLEFRIISQKAADVQPYMLDCRPGEALAVNFAFQLHHMPDE
PpSCL9   DEDRVHIIDFQIAQGSQWVTLIQALAARPRGPPHVSITGIDDPVSNYARGGGLELVGKRLSKLAEICNVPFEFHPVPVMGPDVQVNMLKYQAGDALAVNFSFQLHHMPDE
PrSCL10  DKDRVHIIDFQIAQGSQWVTLIQAFAARQGGSPHVRITGVDDPQSEYARGQGLNLVGERLSKLAESYQVPFEFHGLSVFGSDVHAEMLKIRPGEALAVNFPLQLHHMPDE
PtSCL20  DKDRVHIIDFQIALGTQWVPVIQAFAARQGGSPHVRITGVDDPVSEFARGQGLQMVGQRLSKLAETYQVPFEFHALPVYASDVHADMLDIRPGEALAVNFHLVLHHIPDE

         _______________________PFYRE___________________________________________________________     ____________________________
                                   *                   *          *                       *   *              *      *    *      *
AtPAT1   SVSTENHRDRLLRMVKSLSPKVVTLVEQESNTNTAAFFPRFMETMNYYAAMFESIDVTLPRDHKQRINVEQHCLARDVVNIIACEGADRVERHELLGKWRSRFGMAGFTP
AtSCL1   SVTTVNQRDELLHMVKSLNPKLVTVVEQDVNTNTSPFFPRFIEAYEYYSAVFESLDMTLPRESQERMNVERQCLARDIVNIVACEGEERIERYEAAGKWRARMMMAGFNP
AtSCL5   SVTVENHRDRLLRLVKHLSPNVVTLVEQEANTNTAPFLPRFVETMNHYLAVFESIDVKLARDHKERINVEQHCLAREVVNLIACEGVEREERHEPLGKWRSRFHMAGFKP
OsCIGR2  SVSTANHRDRLLRMVKSLSPKVLTLVEMESNTNTAPFPQRFAETLDYYTAIFESIDLTLPRDDRERINMEQHCLAREIVNLIACEGEERAERYEPFGKWKARLTMAGFRP
PtSCL7   SVSTKNLRDRLLRMVKSLNPKVVTVVEQEVNTNTAPFLPRFMEALNYYSSVFESLDATIKRDSRDRMNVEKQCLARDIVNIIACEGEERIERYEVAGKWRARMTMAGFSV
PpSCL8   SVSTRNPRDQLLRMVKGLSPKVVTVVEREMNTNTAPFLPRFMEALNYYSAVFESLDVSLKRESRERFNIEKQCLARDIVNIVACEDAERIERYEVAGKWRARMTMAGFTV
PpSCL9   SVNTSNHRDRIIRMAKGLAPKVLTLVEQESNTNTAPFFSRFMETLSYYTAIFESLDVTLTRESKERISVEQHCLARDIVNIIACEGAERIERHELMGKWKSRFTMAGFKP
PrSCL10  SVNTSNHRDRLLRMVKNFSPNVVTLVEQEANTNTAPFFPRFMETLSYYTAMFESLDVTLPRDSKERVSVEQHCLARDIVNVIACEGDERVERHELFGKWRSRLTMAGFKS
PtSCL20  SVSTNNHRDRLLRMVKGFAPSVVTLVEQEANTNTAPFFPRFMETLSYYTAMFESLDLTLPRESKERVSVEQHCLARDIVNIIACEGAERVERHELFGKWRSRLTMAGFKS

         ___SAW_________________________________________
                                          *       ***
AtPAT1   YPLSPLVNSTIKSLL-RNYSDKYRLEERDGALYLGWMHRDLVASCAWK--------
AtSCL1   KPMSAKVTNNIQNLIKQQYCNKYKLKEEMGELHFCWEEKSLIVASAWR--------
AtSCL5   YPLSSYVNATIKGLL-ESYSEKYTLEERDGALYLGWKNQPLITSCAWR--------
OsCIGR2  SPLSSLVNATIRTLL-QSYSDNYKLAERDGALYLGWKSRPLVVSSAWH--------
PtSCL7   YPLSANVKDTVKSLLHQSYCNSYKTKEEGGAMYFGWLDRILIAASAWH--------
PpSCL8   YPINTSVDNSIRPLF-ESRGNNYRLKAEKGALHLGWLDKVLVVVSAWHLESHSMIK
PpSCL9   YPLSSIVNSTIKSLL-EGYCENYTLLERDDTLYLGWLNRPLIAAAAFQ--------
PrSCL10  YPLSAHVNSTIKVVL-NSYNENYRLIEKDEALYLGWLDRDLIVASAWK--------
PtSCL20  HALSSRVNATIKVAL-ESYNQNYRVVEKDDVLYLGWLGRDLYVSSAWK
```

AtSCL3

```
            _______________________LHRI______________________________                          __________________________
              *  *   ***   *   *    *    *  *                 **     *   *                                   *         *
AtSCL3      RGLYLIHLLLTCANHVASGSLQNANAALEQLSHLASPDGDTMQRIAAYFTEALANRILKSWPGLYKALNATQTRTNNVSEEIHVRRLFFEMFPILKVSYLLTNRAILEA
PtSCL21     GQEQQPWGAQKDKRYVFNGNMEQTNLCLEQISVLACVTGDPMQRLATYFMEGLAARITKSWPGLYKALNSTHLQP-SMADLVSARQVFFTLFPYLKFAFNTVHLTILES

            ____VHIID________________________________       ____________________________________LHRII____________________
                   *  *       *   *                        *      *      *        *      *    *      **     *     *
AtSCL3      MEGEKMVHVIDLDASEPAQWLALLQAFNSRPEGPPHLRITGVHHQKEVLEQMAHRLIEEAEKLDIPFQFNPVVSRLDCLNVEQLRVKTGEALAVSSVLQLHTFLASDDD
PtSCL21     MEKEKVVHIIDLDASEAAQWIPLLQSLSRRPGGPPHLKMTGINEKKEVVQQTAAILTEEAERLDIPFQFNQLVTRLDRLTDDMLAVKTGEAVAISAVLQFHALLAEDQD

                                                                                                           ________________
AtSCL3      ------------------------------------------------LMRKNCALRFQNNPSGVDLQRVLMMSHGSAAEARENDMSNNNGYSPSGDSASSLPLPSSGR
PtSCL21     FDHNFGGGGGGGGGGGGGGGGGGRVGINRKRPRQSCTTLGDEDLILRSMFNMEALVKKEGSVGRPKYSPSEIEAAISSRRPNLEAAGARSNVEEVRNGRRSTIVE-GN

            __________________________PFYRE_______________________________________  ________________________________SAW_
                        *               *           *                 *   *             *    *    *    *       *
AtSCL3      TDSFLNAIWGLSPKVMVVTEQDSDHNGSTLMERLLESLYTYAALFDCLETKVPRTSQDRIKVEKMLFGEEIKNIISCEGFERRERHEKLEKWSQRIDLAGFGNVPLSYY
PtSCL21     IERFLRMLRGLSPKVMVVVEQESNHNGEHLMDRFVEALYYYSAMFDSLESTLPRQSIERVTVEKYLFGKQIHNIVACEGSERVERHEKLQGWMKRLDRTGFAQLPLGYT

            _________________________________________
                                          *        ***
AtSCL3      AMLQARRLLQGCGFDGYRIKEESGCAVICWQDRPLYSVSAWRCRK
PtSCL21     TLLHANNLLHTYTSHSYRLLHHEGCLTICWEERPLYSVSAWQSA-
```

DELLA

```
         __________________________LHRI____________________________                  ____________________________VHIID___
           * *   ***  *  *       *   *  *               **   *  *   *                            *          *             *
AtGAI    NGVRLVHALLACAEAVQKENLTVAEALVKQIGFLAVSQI-GAMRKVATYFAEALARRIYRLSPSQ-SP-IDHSLS-DTLQMHFYETCPYLKFAHFTANQAILEAFQGKKRV
AtGRS    NGVRLVHALMACAEAIQQNNLTLAEALVKQIGCLAVSQA-GAMRKVATYFAEALARRIYRLSPPQ-NQI-DHCLS-DTLQMHFYETCPYLKFAHFTANQAILEAFEGKKRV
AtRGL1   TGVRLVHALLACAEAVQQNNLKLADALVKHVGLLASSQA-GAMRKVATYFAEGLARRIYRIYPRD-DVA-LSSFS-DTLQIHFYESCPYLKFAHFTANQAILEVFATAEKV
AtRGL3   TGVRLVQALVACAEAVQLENLSLADALVKRVGLLAASQA-GAMGKVATYFAEALARRIYRIHPSA-AAI-DPSFE-EILQMNFYDSCPYLKFAHFTANQAILEAVTTSRVV
OsGAI    AGIRLVHALLACAEAVQQENFAAAEALVKQIPTLAASQG-GAMRKVAAYFGEALARRVYRFRPADSTLL-DAAFA-DLLHAHFYESCPYLKFAHFTANQAILEAFAGCHRV
PtSCL14  SGIRVVHLLMGCAEAIQRNNLKVASDLVREIRMTVNSAPCGAMGKVASHFVEALARRICGLNGAESNMS-QADAQSEILYHHFYEVCPYLKFAHFTANQAILEAFEGHGSV
PtSCL15  SGVRLVHTLMACAEAVQRGNLAIAREMVKEVRILASAQG-GAMSKVATYFAEALARRIYGFLPQDTLRFNQNDPLSDFLQFHFYQTCPYLKFAHFIANQAILDAFSGHQQV

         _____________________________________          ________________LHRII______________                 ________________
            *        *   *                              *      *      *          **     *                    ***
AtGAI    HVIDFSMSQGLQWPALMQALALRPGGPPVFRLTGIGPPAPDNFDYLHEVGCKLAHLAEAIHVEFEYRGFVANTLADLDASMLELRPS-----EIESVAVNSVFELHKLLGR
AtGRS    HVIDFSMNQGLQWPALMQALALREGGPPTFRLTGIGPPAPDNSDHLHEVGCKLAQLAEAVHVEFEYRGFVANSLADLDASMLELRPS-----DTEAVAVNSVFELHKLLGR
AtRGL1   HVIDLGLNHGLQWPALIQALALRPNGPPDFRLTGIG----YSLTDIQEVGWKLGQLASTIGVNFEFKSIALNNLSDLKPEMLDIRP------GLESVAVNSVFELHRLLAH
AtRGL3   HVIDLGLNQGMQWPALMQALALRPGGPPSFRLTGVGN--PSNREGIQELGWKLAQLAQAIGVEFKFNGLTTERLSDLEPDMFETRT------ESETLVVNSVFELHPVLSQ
OsGAI    HVVDFGIKQGMQWPALLQALALRPGGPPSFRLTGVGPPQPDETDALQQVGWKLAQFAHTIRVDFQYRGLVAATLADLEPFMLQPEGEADANEEPEVIAVNSVFELHRLLAQ
PtSCL14  HVIDLNLMHGLQWPALIQALALRPGGPPLLRLTAIGPRQPDGRDVLQEIGMKLAQFAESVNVEFDFRGVMADKLEDIKPWMFQVK-------PGEVVAVNSVLQLHRLLYM
PtSCL15  HVIDFNLKQGIQWPALIQALALRPGGPPAFRLTGIGPPQPDGTDALQEVGTRLHQFAESVNVKFSFRGYVATSLADIKPWMLDAR------PELEAVAVNSILELHRLLED

         ________________________PFYRE________________________________________________________________   ___________________
                          *                 *          *                                  *   *                  *    *    *
AtGAI    ----RPGAIDKVLGVVNQIKPEIFTVVEQESNHNSPIFLDRFTESLHYYSTLFDSLEG--------------VPSGQDKVMSEVYLGKQICNVVACDGPDRVERHETLSQW
AtGRS    ----RPGGIEKVLGVVKQIKPVIFTVVEQESNHNGPVFLDRFTESLHYYSTLFDSLEG--------------VPNSQDKVMSEVYLGKQICNLVACEGPDRVERHETLSQW
AtRGL1   ----HPGSIDKFLSTIKSIRPDIMTVVEQEANHNGTVFLDRFTESLHYYSSLFDSLEG---------------PPSQDRVMSELFLGRQILNLVACEGEDRVERHETLNQW
AtRGL3   ----QPGSIEKLLATVKAVKPGLVTVVEQEANHNGDVFLDRFNEALHYYSSLFDSLEDGV------------VIPSQDRVMSEVYLGRQILNLVATEGSDRIERHETLAQW
OsGAI    ----QPGALEKVLGTVHAVRPRIVTVVEQEANHNSGSFLDRFTESLHYYSTMFDSLEGGSSGQAELSPPAAGGGGGTDQVMSEVYLGRQICNVVACEGAERTERHETLGQW
PtSCL14  DAPTGSSPIDVVLKSIGSLRPKIVTVVEHEANHNGPVFLDRFVEALHYYSTMFDSLEACN-----------VLPNSMEKFLAELYIQKEICNIVACEGRYRIERHETLSHW
PtSCL15  PIPGRPSAIDRVLASIWSLKPKILTVVEQEADHNRPVFLDRFTEALHYYSTVFDSLEARG-----------LQAQSEEQVMSEVYLGREICNIVACERSERVERHEPLLNW

         ______________________SAW_____________________________________
                 *                                        *        ***
AtGAI    RNRFGSAGFAAAHIGSNAFKQASMLLALFNGGEGYRVEESDGCLMLGWHTRPLIATSAWKLSTN---
AtGRS    GNRFGSSGLAPAHLGSNAFKQASMLLSVFNSGQGYRVEESNGCLMLGWHTRPLITTSAWKLSTAAY-
AtRGL1   NRFGLGGFKPVSIGSNAYKQASMLLALYAGADGYNVEENEGCLLLGWQTRPLIATSAWRINRVE--
AtRGL3   RKRMGSAGFDPVNLGSDAFKQASLLLALSGGGDGYRVEENDGSLMLAWQTKPLIAASAWKLAAELRR
OsGAI    RNRLGRAGFEPVHLGSNAYKQASTLLALFAGGDGYRVEEKEGCLTLGWHTRPLIATSAWRVAAA---
PtSCL14  RIRLGRAGFRPSHLGSNAFKQARMLLTLFSG-EGYTVEENNGSLTLGWHSRPLIAASAWQGS-----
PtSCL15  SVRLRNAGFWPIPLGSNAFKQASMLLSLFSGGEGYRVEENNGCLTLGWHSRPLIAASAWQRC-----
```

HAM

```
         ______________________LHRI________________________                          ______
         * *  ***  *  *      *   *  *            **   *  *   *
PhHAM    -QFIYDQIFQASELLLAGHFSNAQMILARLNQQLS-PIGKPFKRAAFYFKEALQLPFLLPCTS------------TFLPPRSPTPFDCVLKMDAYKSFS
AtSCL6   -VGITEQLVKAAEVIES-DTCLAQGILARLNQQLSSPVGKPLERAAFYFKEALNN--LLHNVS------------QTLN-----PYSLIFKIAAYKSFS
BnSCL1   -FDYIEDLIRVVDCIESDELHLAHVVLSQLNQRLQTSAGRPLQRAAFYFKEALGSLLTGTNRN-----------------QLFSWSDIVQKIRAIKEFS
PtSCL12  VHVIVEQLLKAAEAVELGNLDHAQAILARLNQHLS-PLGKPLQRAAFYFKEALASRILNTTATNTGGDNRNATVTGTATSNISSPLDMVHKISAYKSFS
PtSCL13  KSAILQQLLNATNYVELGSLDIVQAILARLNQYVS-PRGKALQRATYYFKEALGGLRSLQ---QTNSKSYLSPLQLVHKINACKNFS

         __________________VHIID________________________________________          ________________LHRII__
           *         *                *  *       *   *                            *      *      *
PhHAM    EVSPLIQFMNFTSNQAILEALGDVER---IHIIDFDIGFGAQWSSFMQELPS-SNRKATSLKITAFASPSTHHSVEIAIMHESLTQFANDAGIRFELEV
AtSCL6   EISPVLQFANFTSNQALLESFHGFHR---LHIIDFDIGYGGQWASLMQELVLRDNAAPLSLKITVFASPANHDQLELGFTQDNLKHFASEINISLDIQV
BnSCL1   GISPIPLFSHFTANQAILDSLSSQSSSPFVHVVDFEIGFGGQYASLMREIAEKSANGGF---LRVTAVVAEDCAVETRLVKENLTQFAAEMKIRFQIEF
PtSCL12  EASPLAQFAHFTANQAFLEALEGAEA---IHIIDFEIGLGGQWASFLQELAVKLGGAPP-VRITALGS-SASSSLELHLTRDNLCNFAKQLNVPFEFEL
PtSCL13  EISPIPYFAHFTANQVLLEALEAVDK---IHIIDFDMGLGGQWASFLQELALRPGG-PP-VRITALGTAVGYESMEMHLIRENLCTFAENLNIPFTFQV

         __________       __________________________________________PFYRE ____________________________
         **      *        ***                          *                          *            *
PhHAM    INLDTFDPKSYPLSSLRSSDCEAIAINFPIWSISSCLF--AFPSLLHYMKQLSPKIIVSLE----------RGCERTELP--LKHHLLHALQYYEILLA
AtSCL6   LSLDLLGSISWPNSSEK----EAVAVNISAASFS------HLPLVLRFVKHLSPTIIVCSD----------RGCERTDLP--FSQQLAHSLHSHTALFE
BnSCL1   VLMKTFEILSFKAIRFVDGERTVVLISPAIFRRVIG-----IAEFVNNLGRVSPNVVVFVDS---------EGCTETAGSGSFRREFVSAFEFYTMVLE
PtSCL12  LHFDHIESLTLR---ER----EAVAVNLSLLPSTFSSPD-SISRLLRLVKNLAPRAVVVVDAETTATAAASAATTSSAS--FVHHFLEALQFYSFLFD
PtSCL13  VEIPQNEDLNRSMLNLKEG--ETIAVNYSLGMQGLFSKD-SIASILHLINRLRPKIVVVVD----------HENEQAGSS--FAQKFMEALQFYALLFE

         _________________________     _________________________________________SAW__________________________
                       *                              *       *
PhHAM    SIDAANVTPEIGKKIEK-SLLLPSIENMVLGRLRSPDR---MPQWRNLFASAGFSPVAFSNLTEIQAECVVKRTQVGG---FHVEKRQSS--LVLCWKQ
AtSCL6   SLDAVNANLDAMQKIER-FLIQPEIEKLVLDRSRPIERP--MMTWQAMFLQMGFSPVTHSNFTESQAECLVQRTPVRG---FHVEKKHNS--LLLCWQR
BnSCL1   SLDAAAPPGDLVKKIVETFLLRPKISAAVETAANRRSAG--QMTWREMLCAAGMRPVQLSQFADFQAECLLEKAQVRG---FHVAKRQGE--LVLCWHG
PtSCL12  SLDAVNINMDAVHKIEK-FLLAPKIDATISSAA--------APPWKTLFAAAGFSPVAFSNFTETQAEYLIQRLHSRG---FEVEKAHAA--LLLGWQG
PtSCL13  SLEAVHMNMETIEMIEK-FVMSPRICNVVEAAYRRHREGENLPNWRSMFQASGFTPMMMSNFTHKQAESLSRSRQQRFGFCFEAVKKQQEQILLLGWQR

         _________
               ***
PhHAM    QELLSALTWRC----
AtSCL6   TELVGVSAWRCRSS-
BnSCL1   RALVATSAWRF----
PtSCL12  RPLVSATAWRCGPPP
PtSCL13  QILVSVSAWIVNNVV
```

LS

```
        __________________________LHRI____________________________                                  ________
        *      ***  *  *      *     *  *             **   *  *   *
LsLS    PAIQIRQLLISCAELISQSDFSAAKR-LLTILSTNSSPFGDSTERLVHQFTRALSLRLNRYISSTTNHFMTPVETTPTDSSSSSSLALIQSSYLSLNQVT
AtSCL4  LEPPLLKAIYDCARISDSDPNEASKT--LLQIRESVSELGDPTERVAFYFTEALSNRLSP---NSPATSSS---SSSTEDLILS--------YKTLNDAC
OsMOCI  AAPSTRDLLLACADLLQRGDLPAARRAAEIVLAAAASPRGDAADRLAYHFARALALRVD----AKAGHGHVVVGGGAARPASSG-------AYLAFNQIA
PrSCL2  QRSDLLHSLLDCAKIVDTEPERAGQS--VAYLQRIASHHGDPTQRIASHFADALAKRLSKGIEQKPQFQSSDECSKSFEDLTLA--------YKALNDAC
PtSCL17 SAAHLRNLLTECAEFVAQCEW-QRADRMISILSTRGSPRGDSTERLVAQFTRALRHRSDLALARRPPPDGATERELES------------AYLCLNQVT

        __________________________VHIID_________________________________           __________LHRII__________
        *         *              *  *       *   *                                  *      *      * **     *
LsLS    PFIRFTQLTANQAILEAINGNHQAIHIVDFDINHGVQWPPLMQALADRYP----APTLRITGTGN------DLDTLRRTGDRLAKFAHSLGLRFQFHPLY
AtSCL4  PYSKFAHLTANQAILEATEK-SNKIHIVDFGIVQGIQWPALLQALATRTSGK--PTQIRVSGIPAPSLGESPEPSLIATGNRLRDFAKVLDLNFDFIPIL
OsMOCI  PFLRFAHLTANQAILEAVDG-ARRVHILDLDAVHGVQWPPLLQAIAERADPALGPPEVRVTGAGA------DRDTLLRTGNRLRAFARSIHLPFHFTPLL
PrSCL2  PYSKFAQLTGNQAILEAMDK-AEKIHIVDFGIVQGVQWAALLHAFATRPGGK--PHKIKITGIPAPTLGQNPTSSLLATGKRLTEFAKLLDLEFEFCPVS
PtSCL17 PFIRFAHLTANQAILETLDTDSPSIHVVDINIMQGVQWPPFMQALAERPGGP--PSHIRLTGAGA------DAEALRKTGRRLERFAHTLGLRFEFNPWI

                                               __________________________________________PFYRE______________
                                               ***                           *                      *
LsLS    IANNNHDHD-------------EDPSIISSIVLLPDETLAINCVFYLHRLLKD-REKLRIFLHRVKSMNPKIVTIAEKEAN------HNHPLFLQRFIEA
AtSCL4  TPIH--------------------LLNGSSFRVDPDEVLAVNFMLQLYKLLDETPTIVDTALRLAKSLNPRVVTLGEYEVS------LNRVGFANRVKNA
OsMOCI  LSCATTAPHHVAGTSTGAAAAASTAAAATGLEFHPDETLAVNCVMFLHNLAG--HDELAAFLKWVKAMSPAVVTIAEREAGGGGGGGDHIDDLPRRVGVA
PrSCL2  KHMS--------------------EVELSTLKIEQDECIAVNFMLQLYNLLGDSPEPLVKILKLAHALSPKVVTLGEYEAH------LNACQFQVRFRNA
PtSCL17 VDDDI---------------------SSLAISIRPGESLAINCVLHLHELLDEAHNERLKNFLVRIRSLEPKVVALAEREAD-----HNRPTFTDRFVEA

        ____________________________________        ___________________________________SAW__________________
           *                 *   *                        *    *       *
LsLS    LDYYTAVFDSLEATLPPGSRERMTVEQVWFGREIVDIVAMEG-DKRKERHERFRSWEVMLRSCGFSNVALSPFALSQAKLLLRLHYPSEGYQLGVS-SNS
AtSCL4  LQFYSAVFESLEPNLGRDSEERVRVERELFGRRISGLIGPEKTGIHRERMEEKEQWRVLMENAGFESVKLSNYAVSQAKILLWNYNYSNLYSIVESKPGF
OsMOCI  MDHYSAVFEALEATVPPGSRERLAVEQEVLGREIEAAVGPSG-GRWWRGIER---WGGAARAAGFAARPLSAFAVSQARLLLRLHYPSEGYLVQEA-RGA
PrSCL2  LEYFSAFFESMEPNMARDSAERLNVEKHFFAEKIMGIVAFEG-AERKIRLEGRDQWRIVMESAGFKFTNLSHYARSQARILLYNY--CEAYSLDES-SGF
PtSCL17 LNHYSILFDSLEATLPPKSQERLDVEQVWFGREITNIVALEG-HGRNERHQKFERWSELMNECGFTSLPLSEFALSQARLLLRLHYPSEGYQLHT-QNKA

        ________________
            *        ***
LsLS    FFLGWQNQPLFSISSWR----
AtSCL4  ISLAWNDLPLLTLSSWR----
OsMOCI  CFLGWQTRPLLSVSAWQPSSS
PrSCL2  LSLAWQNRPLLTVSAWCCC--
PtSCL17 AFLGWQNSPLFSVSSWH----
```

AtSCL26

```
            _______________________LHRI______________________________
             * ** **   *  *        *   **  * **       *  *   *   **
AtSCL26     GLRLVHLLVAAADASTGANKSRELTRVILARLKDLVSPGDRTNMERLAAHFTNGLSK-----------------------------------------------------
PtSCL11     QQDLLQILVKCAEALSEG--HHGLVRSMVSKLQEFSSPLG-DPLQRISFYVSEVLKN-----------------------------------------------------
PtSCl16     GLRLIHLLTACAEAMLEG--AQDLVEVILCRLRQLVSCTG-STMERVGYYLFDALLH-----------------------------------------------------
PtSCL19     GLRLLHLLTACAEAKSLG--AQDLVEVILCRLRGLVSPTG-FTMERVAYYVFDALLH-----------------------------------------------------
PtSCL22     GLRLVHLLTACAEAMSQE--AQDVVEVILCRLRDLVSPTG-SSMERVAYYLYHAL-------------------------------------------------------
PtSCL23     GLRLLHLLTACAEAISEG--TQDLAEVILCRLRGLVSPTG-STMERVAYYLFNALVCS----------------------------------------------------
PtSCL24     GLRLIHLLTACAEAMSEG--AQDLVEVILCRLRQLVSCTG-STMERVAYYLFDALLH-----------------------------------------------------
PtSCL25     GLRLVHLLTACAEALSQG--AEDVVEVILCRLRELVSLTG-STMERVAYYLYHALLHSNVNSLVHNMEPNDKSSKYHFLGALKLLHVAYPYIQIPHFTANQCILEAVAAA
PtSCL26     GLRLLHLLTACAEAMSQG--AQDLVEVILCRLQGLVSPTG-STMERVAYYLYHAL-------------------------------------------------------
PtSCL27     GLRLLHLLTACAELMSEG--AEDLVEVILCRLRGLVSSTG-STMERVAYYLYRALLH-----------------------------------------------------
PtSCL28     GLRLLHLLTACAEAMCEG--AQDLVDVILWRLRGLVSSTG-TTMERVAYYLYRALLH-----------------------------------------------------
PtSCL29     GLRLLHLLTACAEAMSHG--AQDLVEVILCRLRQLVSSTG-STMERVAYYLLDALLHY----------------------------------------------------
PtSCL30     GLRLLHLLTACVEAMSEG--AEDLVEVILCRLRQLVSCTG-STMERVAYYLFDALLH-----------------------------------------------------

                                                           ________________________________VHIID__________________________
                                                                    *          *                   *  *       *   *
AtSCL26     ------------------LLERDSVLCPQQHRDD----VYDQADVISAFELLQNMSP-YVNFGYLTATQAILEAVKYER------RIHIVDYDINEGVQWASLMQALVSR
PtSCL11     --------------------HSFSVVNQEENDEE------SSLEYDLAYQAFYQLLP-YHKFLHFTSNQAIQEAVDNASN------IHIIDLEIRQGLQWPSFIQSLAHR
PtSCl16     -------------------SNVNSLAH-LELNDK-----TTKEHFLGALKLLHAAYP-YIRIPHFTANECILEAVVAVGGGNN-VGIHIIDFDIMEGMQWPPLMETLRNK
PtSCL19     -------------------SNVNSLPH-LELNDK-----ISKDHFLGALKLLHVAYP-YIRFPHFTANQCILEAVAG--AANN-VGIHIVDFDVMDGMQWPPLMEALRNS
PtSCL22     ---------------ALLHSKVNSLAH-LEPNDN-----SSKDHFLGALKLLHVTYP-YIQIPQFTAYQCILEAVAAGGSGNN-VGIHIIDFDIMEGMQWPPLMETLRNN
PtSCL23     -------------------NNINSLGH-LEPNDKSMSTSSAKEHFLGALKLLHVAYP-YIRIGHFTANQCILEAVAGTGSTN--VGIHIIDFDIMEGMQWPPLMEALRSN
PtSCL24     -------------------SNVNSLPR-LELSDK-----TAKEHFLGALKLLHVAYPYYIRIPQFTANECILEAVVAAGGVNN-VGIHIIDFDIMEGMQWPPLMEELRNN
PtSCL25     GGGSTMERVAYYLYHALLHSNVNSLVHNMEPNDK-----SSKYHFLGALKLLHVAYP-YIQIPHFTANQCILEAVAAAGGGNN-VGIHIIDFDIMEGMQWPPLMETLRNN
PtSCL26     -----------------LQSNGNSLAH-LERNDK-----SSKEDFLVALKLLHVAYP-YIQIPHFTANQSILEAVAAAGSDKN-VGIHVIDFDIMECMQWPPLMEALKSN
PtSCL27     -------------------FNVDSLAHLMEPNDE-----SSNDNFLGALQLLHVAYP-YVRIPHFTANQCILEAVAGAGCTNNNVRIHIIDFDIMEGMQWPPLMESLKN-
PtSCL28     -------------------SNADSFAHLMEPTDE-----SSNDSFLGALQLLHVAYP-YVRIPHFTANQCILEAVAGAGGINNNVRIHIIDFDIMEGMQWPPLMESLKN-
PtSCL29     -----------------SNVNINSLAH-LELDDK-----TGKEHFLGALKLLHVAYP-YIRIPHFTANECILEAVVAAGGGNN-VGIHIIDLDIMEGMQWPPLMEALRNN
PtSCL30     -----------------SNVNIDSLTH-LELDDK-----TGKEHFLGALKLLHVAYP-YIRIPHLTANECILEAVVAAGGGNN-VGIHIIDFDIMEGMQWPPLMEALRNN

                                                __________LHRII______________        ____________________________________
                                                 *   *  * *          *  *    *        ***                            *
AtSCL26     NTGPSAQHLRITALSRATNGKKSVAAVQ----ETGRRLTAFADSIGQPFSYQHCKLDTNAFSTSSLKLVRGEAVVINCMLHLPRFS-HQTPSSVISFLSEAKTLNPKLVT
PtSCL11     PG-GPPKLLKITAIGQDEKR----------LKQTGRRLLEFAESMEIAFAFHPVVVDLENLDESALNIEAHETIAVNCSVVLHRLLCKSGK--IHGLLALLRKLNPAVLT
PtSCl16     ---DDVRHLRITTIKWADDEEDEFLRYS---GHTGRWLSEYAASAGIPFWYE--KMDLKGVKD-ILPSQEGEIVVVNCMWQLPHMLNPRNKSHLLEFLVGAHHLNPVAVT
PtSCL19     GKNYDVSHLRITAIKWTEDEDDGCLLYS---GHTGRRLSAYASSVGIPFSFQ--EINLKALKE--VVPQETEIVIVNCIWQLPHMLNLRNKSQLLEFLVGSRRLNPKAVT
PtSCL22     G----VGYLRITAIKWDEDE-EECVVYSG--RHTGRRLSEYASSVGIPFSFE--ETDLKGLKEGLLPSREGEIVIVNCIWQLPHMVNLRNKSELMEFLVGARHLNPEAVT
PtSCL23     GD-YEIDHLRITTVKWVDDDDDAAVLYTECCKHTGRRLSEYAESLGIPFLFQ--ETDLKGLKD--LMSEDGEIVIVNCMWQLPHMVKLRNKKQLLEFLGGARHLNPEAVT
PtSCL24     ---YDVRHLRITAIKWADDEEDEFLHYS---GHTGRRLSEYATSAGIPFSYQ--ETDLNDMKD-ILPSEEGEIVVVNCMWQLPHMLNLRNKSHLLEFLVSTRHLNPVAVT
PtSCL25     GN-YDVAHLRITAIRWAEDN-EEWLLYSG---HTGRRLSEYASSVGLPFSFQ--EADLKELKEGLLPSREGEIVMVNCIWQLPHMVNLRNKSEVMEFLVSVRHLNPEAVT
PtSCL26     GN-YDVGRLRITAIRWAEQD-GECPLYSG---HTGRRLSEYASSVGIPFSFQ--ETDLKGLKE-VLRSHEGEIVIVNCMWQLPHMFNLRDKSQLLEFLVSARHLNPKAVT
PtSCL27     ---IDVAHLKITAIKWADEE-DECVLYS---GNTGRRLSEYASSAGIPFSYQ--ETDLEGLKD-ILPSEEGEILVLNCMWQLPHMLNLRNKSHVLQFLVGARRLNPVAVT
PtSCL28     ---IDVAHLTITAIKWAAEEEDECVLYS---GHTGRRLSEYASSAGIPFSYQ--ETDLEGLKG-IPLSEEGEIVVVNWMWQLPHMLTLRNKSHLLQFLVGARCLNPVAVT
PtSCL29     ---DDVPHLRITAIKWPDDDEDQCLHYSS--AHTGRRLSEYATSAGIPFSYQ--ETDLKGLND-ILPSHEREIVVVNCMWQLPHMLNLRNKSHLFEFLVGSRHLNPAVVS
PtSCL30     ---DDVPHLRITAIKWPDEEEDQCLHYS---AHTGRRLSEYATSAGIPFSYQ--ERDLKGVKD-ILSSQEGEIVVVNCMWQLPHMLNLRNKSHLFEFLVSTRHLNPVAVT
```

(AtSCL26 cont.)

```
                ___PFYRE______________________________________________________         _________________________________SAW___
                              *              *                   *                        *     *       *
AtSCL26         LVHEEVG--LMGNQGFLYRFMDLLHQFSAIFDSLEAGLS-IANPARGFVERVFIGPWVANWLTRITA---NDAEVESFASWPQWLETNGFKPLEVSFTNRCQAKLLLSLF
PtSCL11         VMEIECN---QSMPSTVGRFLQCLIFHRAVFNSIEAIVE-KNNPDRVLIERVYVAPNISNVLVHDKE---NSGMYACIDSWRKFLRHSGFKDSPLSNYSKCQANLLLGMY
PtSCl16         LATGPHG-TESDSANFVDSLVNRLKELCAIFDSLEAGLP-EHGLARTMVERIFFGPAMSKRVMSSAR-GDDRPADTTNSNVVDLPLRCGYEECNISNDNMIYAKYNVLMC
PtSCL19         LATGPHG-AESGAADFLDNFTRRLWELCTIFDSLEAGLP-EHGLARAMVERIFFGPAMSRRVTSGTACGDHGAADTTKSNVVDLPIKCRYGEGSISSSTIMYAKGTLLCS
PtSCL22         LATGAHG-AEGNMSNFLDSFSRCLEELCTIFDSLEAGLP-QHGLARAMVERFFFGPAMAKRIVSS---SACGPPDTRKSNVVDLPLECGYGEGSISNDNMLYAKYTLQSG
PtSCL23         VVTGPHGNDIAERGDFIESFAGCLKDLCTIFDSLEAGLP-EHGLARAMVERMFFGPAMSRRLMKS--------PENGESNVVDIAVKCGYGEGTITSENVMYAKYTLELC
PtSCL24         LATGPHG-IESETANFLESFARRLEELCAIFDSLEAGLP-EHGLARAMVERIFFGAAMSKRVMSSAR-GDDRVADTTNSNVVDLPLRCGYEEGSISNDNMINAKCNLLWN
PtSCL25         VATGPHG-SEGNMSNFLESFCRCLEELCAIFDSLEAGLP-QHGLARAMVERFFFGPAMAKRIVNS---IECDPPDTRKSNVLDLPQECGYGEVSISNDNMLYAKYTLHNC
PtSCL26         LATGPHG-AERNRANFWESFCRCLQDLCAIFDSLEAGLP-HHGLARAMVERFFFGHAMAKTIVSS---SACGPPDNRKSNVVDLPLDCSYSAGSISKENMLYAKYTLQSV
PtSCL27         LATGPHG-TESNTANFLESFVRRLEELCAIFDSVEAGLP-EHGLASAMVERFFFGPAMSKRVMSSVR-GHDSPADSTDSNVADLPLKCGYGGQGISNENVIYAKYSLPCS
PtSCL28         LATGPHG-TESGTANFLESFVRRLEELCAIFDSVEAGLP-EHGLASAMVERFFFGPAMSKRVLS------ASSDDSRDSNVVDLPFNCGYGEGSISNENMICAKYSLPCS
PtSCL29         IATGPHG-TENESGNFVESFARRLEELCAIFDSLEAGLPPEHGLARAMVERIFFGAAMSKRVISSAR-GDDRPVDATNSNVVDLPLRCGYEECSVSNENMIYAKSSVLMC
PtSCL30         LATGPHG-IESETANFLESFARRLEELCAIFDSLEAGLP-EHGLARAMVERIFFGPAMSKRVMSSVR-GDDSLADIRDSNVVDLPLRCGYEECSISNDNMIDAKCNVLCS

                __________________________________
                                       *        ***
AtSCL26         N------DGFRVEELGQNGLVLGWKSRRLVSASFWASCQTNQ----------------------
PtSCL11         PN-----DSFKLHQDG-VSITLAWQDTPIVSVSVWTC---------------------------
PtSCl16         ---SVSGGCYEVELVGEHRLVLKSGCTPLVWVSTWKSPQL SEFDSLNFLEKN------------
PtSCL19         ----GGNASYEVELVGRHRLVLKWASTPLVWISTWKSVNP------------------------
PtSCL22         VT-----GYYEVELVGDHRVVLKWGCTPLVWVSTWKTTR-------------------------
PtSCL23         K---GSGRCYEVALVGRNRLVLKWASTPLVWVSTWKSP--------------------------
PtSCL24         ---GSSG-CYGVELAGHHRVVLRWGSTPLVWVSTWKSP--SGIVRQGVPG--------------
PtSCL25         VGR----GSYDVELAGDHRLVLKWGCTPLVWVSTWTTWK-------------------------
PtSCL26         GR-----GSYEVEMVGDHRLVMKWGCAPLVWVSTWKST--------------------------
PtSCL27         ----GGRGCYEIEMVGDHRLVLKWGSTPLVWCGYSGWGISNENLIYAKYSLPCSGRRGCYEIEM
PtSCL28         -------GSYEVEKLGDNRVVLKWGSTPLVWVSTWKSPSSHHHDLTNQHWNDTDFILG------
PtSCL29         SG-SGSGGCYEVELVGHHRLVLKWGCTPLVWVSTWKTPSSSCGYPIHVTN--------------
PtSCL30         ----GSSGCYEVEMVGHHRVVLRWGSTPLVCVSAWKSPSSSCISNRR-----------------
```
