# Supplementary material for: The GRAS gene family in pine: transcript expression patterns associated with the maturation-related decline of competence to form adventitious roots
Source: BMC Plant Biol. 2014 Dec 30;14:354. doi: 10.1186/s12870-014-0354-8 (PMC4302573; doi:10.1186/s12870-014-0354-8)
Supplement: Additional file 4: — Deduced amino acid sequence of GRAS proteins from pine. Basic and acidic amino acids, as well as stretches of different amino acids are highlighted in the N-terminal region. [file 12870_2014_354_MOESM4_ESM.pdf]

acidic basic Ser stretches

other stretches GRAS region

>PrSCR

MAACIAMNTRFRGGDASGEEVFVVGETDSNNSMIIPASEGENRRKRTGMELNGPLFRRINPSESSDYGGQIKW
NNLSSGLRESQSPVPLASAAGWQYPSSATVDSVSHRISAPSRTPGLGLANSVLSGESNGQPFNGAYSNMPFF
QNSNMERQVSDSCSQWNKWGSVVQPEVGLGCDGTGQQLSSLNAVQTMDEAAAAVWVDGMIKDLMMQSSPNVS
MAQIIQSLQEIALYNPHIASVLEYRLRSLGQSELGLAGNTQIGRDQNVGMNVSSDGRGFKRSMDQSDHHRRD
MNSQQQIMVDWKSIGANPDSARRSARASQLDDDENRLIDNIHGRGLDVDDQRLRRGNDDLRLRIQSTESHDI
QREHQPDSALKLYLDSSGYDQTSWASSKDQFSETAFMANQSMWVQPRQPDNNSQPPVLSLQHQANEDDPLRV
APSNDNYITMNRRINTNSNQDPSVNQTPQQQGPSDITASDEEGLHLLALLLQCAEAVSADNFEEANTILPQI
TELSTPYGNSVQRVAAYFAEAMSARLVSSCIGMYSPLPPIHMSQSQKIVNAFQVFNGISPFVKFSHFTANQA
IQEAFEREQRVHIIDLDIMQGLQWPGLFHILASRPGGPPHVRITGLGTSLEALEATGKRLSDFAHTLNLPFE
FHPVADKVGKLDPERLKVNRGDALAVHWLHHSLYDVTGSDTNTLRLLQRLSPKVITVVEQDLSHGGSFLSRF
VEAIHYYSALFDSLGASYPEDSHDRHLVEQQLLSREIKNILAVGGPARTGEVKFDNWRDQLKQTGFKPISLA
GNAATQATLLLGMFPCQGYTLMEENGTLKLGWKGLCLLTASAWRPAHHGLIY

>PrSCL2

MSMGGVNMNMNMSPWLSAPTAWQHDGLMSFPPGICTDVVGDLCSTTTNSYLSEPNSVLDLSRASMLFSPMEQ
FQDFTQHLHDGIGAKQMPLLGGVEPPAPAFKDNALYHHEALVKKERPPIKEPGVGDGSSAALRSSVFRSPEL
HTFGFESPVSNRNDFVSPDVCTEWMDCLMSDLQPEAIAGNGNAAIASCFDSSNPCESTESSELPNGGSGYRR
HSSEDAAAWQSEFGILGEAFKRSSPTSWTPDTTTEQLLCSSPSADLLSAASSELLSTNSLKGKAALQEQYGG
AAGGGHEFQTLHHRMPNVHQSVCPPKQQLIREPSTPPQKRQQGQQEQDPLKGKREEDPPKPEIDALDRHMQG
QRSDLLHSLLDCAKIVDTEPERAGQSVAYLQRIASHHGDPTQRIASHFADALAKRLSKGIEQKPQFQSSDEC
SKSFEDLTLAYKALNDACPYSKFAQLTGNQAILEAMDKAEKIHIVDFGIVQGVQWAALLHAFATRPGGKPHK
IKITGIPAPTLGQNPTSSLLATGKRLTEFAKLLDLEFEFCPVSKHMSEVELSTLKIEQDECIAVNFMLQLYN
LLGDSPEPLVKILKLAHALSPKVVTLGEYEAHLNACQFQVRFRNALEYFSAFFESMEPNMARDSAERLNVEK
HFFAEKIMGIVAFEGAERKIRLEGRDQWRIVMESAGFKFTNLSHYARSQARILLYNYCEAYSLDESSGFLSL
AWQNRPLLTVSAWCCC

>PtSCL5

MAESEECNGGYESLLDWLDIGDESSSMDPFINGDSCPIADQDHEDFWEAALPTMDIQSETQFVMEPSFSSND
SVAPAAQKGDAESFWVEPPVNDVEDSPKITKKRKRARVSGESTKGKLWNVFENNAASPHTGESLDHGNHCLA
PETKLSSLHGAESDNQLDLEATRNNSNNNNNNSSNSNNKGRSGGRQSSPPNLPKESRWAEQLLNPCAVAIAN
NNIVRIQHLIWVLHDLASISGDANHRLAAHGLRAFTAKIMHPGASLPCSEFLSADPKLFHKALXQFHEVSPW
YQLLYTIANKYLLEAFEDQNSKALHVIDIGVSHGIQWPTFMEALARRPAGPPSLLRLTIIADTLGAPFSAGP
PSNDFPNRLKRYAKTLNLDMEVNVIAQPLDSLTREALGVKEDEALAICGQFRLHQLLGEGLDKNEGKDNQLT
SVSPRDKFIKFIKDLNPLVFFLSDNDIDHSSPDFIERFKNSVDHLWRFLDSTSVCFKGRECEERRMVEGEAA
MGLVNNVAYDGASRVERNENHLNWANRVREEGFVCEIPSDKIVDCARAMLRKHDSNWEMRLEDGCISLCWKS
HPVTFCSLWKPCFNEPT

>PtSCL6

MKGELEIMGINHQADFQISRHAPVLFSEKRMKLEESGQYLVRNEAAALSPQDCFEATGGQIGWILSESSELG
QPSSVGLTAVTCQESNIDVNSIKARQQSLQFADTSDRPDCTRQVQCIIKNSPKLKSLDSKLAFTPQLRSPSM
AATHREWNASTDLNTNRSHYYEELSTAPADQDQSWPLLGVSIAKGLGNLGACHDASCMEQLLLHCSSALENN
DVTLAQQIMWVLNNIASAEGDPNQRVTSCFLRALIARAARMSNSNMLRAMAATTANAHVGDTKRLSVIELAG
FVDLTPWHRFGFSSSNGAIAEAVEGYSVVHILDFGIAHCMQWPTLIDSLSKRPDGPPYIRLTVSCARPPVPP
LLNMSYEELGTRLSNFARSRNVPFEFRAIPPDPSEEFLVLDQLEPSILDIREGEALVINCQFRLHYIPDETA
DQQSSCSSSSCQRDEFLRIIRSFEPTIMTLVDEDSNLTSTSLVSRLKSAFNYLWIPFDALNTFLPRDSKQRQ
QYESDVGYKIENIIAFEGHQRIERLESKAKWTQRMRKACFQSISFSEDTVAEVKAMLDEHAAGWGLKKEEDD
LLLTWKGHNVVFATAWVPSHL

>PrSCL10

MSLASPCQESMRGDDNSYRTNSWYRGNLSGVPEISRNMGMQYMPGGIGLNLQRGWGNSFRSCSTDLSPKQNG
NFAIDHTSTTGERSAGTLKMIDQRSPISPLSPKGIGSDGLPQTSQSQRLYSSERIQMHDDLIHSSSHNHGSF
NDQVYIHESFPCNNELSRESSICIQSSDGSPLSSQHSQSYSSDSRYFHSAEDAADLVGNAYSGNGQPANLKI
LEEIETALLGPDGDDPLDMDYAYGDHTLNSNSEWSLEALSDFNDFLDQTSNPGYSQINNADSSRVPDNLQQL
TNHGDRNVKEETVNEVNLKIEHFSPSSSDMEKLEIEELGSPQGDVKSLLIECAKAIADGRNADNLIAGLRQV
VNIYGDPLHRLAAYMVEGLVARLHFSGGHIYKTLKCKEPTSSELLSYMHILYEVCPYFKFGYVAANGAIAEA
FKDKDRVHIIDFQIAQGSQWVTLIQAFAARQGGSPHVRITGVDDPQSEYARGQGLNLVGERLSKLAESYQVP
FEFHGLSVFGSDVHAEMLKIRPGEALAVNFPLQLHHMPDESVNTSNHRDRLLRMVKNFSPNVVTLVEQEANT
NTAPFFPRFMETLSYYTAMFESLDVTLPRDSKERVSVEQHCLARDIVNVIACEGDERVERHELFGKWRSRLT
MAGFKSYPLSAHVNSTIKVVLNSYNENYRLIEKDEALYLGWLDRDLIVASAWK

>PtSCL12

MNGMLSRTSGNSLPPAQLKHQILQSKTSPDQAQKRHCKSSVWQRSRSGGEIEPTSVLDIRSPSPTSTLSSSL
GGSSESAGAVAAVSSGLLCEAVSDGGGGGSGSGGAETLLLESSGKEAFNNGGGSTGSPARWYSNRGGSGNGG
REGFGWRLNNGEQQPGGGGMVEGKSEVKKEDPQQHSRAEEWGSGGGAGTAGGMDDLESMLFESGAAPPDQSL
MRWLLGEIEDPKDLPPPQIKSATSGGSTSAPQFVDPSIEPNFADPGAFAFSNNNLSDVIAPPQHASVPSSFR
PSYATLPNLPQQQFIPPPPPPPPPPPFAYNGPMPTPFGPNAYHPEVLFSGPAVYGSNPVQHLGPDFPRFN
MAADPNRNNNNLLFDMPHPPPPKRFGLEHQLWQQTFKQQEYMNMMKPQQQQELLQSLHRRQQFLHQAPPPSP
HHQMRQKAVVNHTFKVGGSGAVADEVHVIVEQLLKAAEAVELGNLDHAQAILARLNQHLSPLGKPLQRAAFY
FKEALASRILNTTATNTGGDNRNATVTGTATSNISSPLDMVHKISAYKSFSEASPLAQFAHFTANQAFLEAL
EGAEAIHIIDFEIGLGGQWASFLQELAVKLGGAPPVRITALGSSASSSLELHLTRDNLCNFAKQLNVPFEFE
LLHFDHIESLTLREREAVAVNLSLLPSTFSSPDSISRLLRLVKNLAPRAVVVVDAETTATAAAASAATTSSA
SFVHHFLEALQFYSFLFDSLDAVNINMDAVHKIEKFLLAPKIDATISSAGAPPWKTLFAAAGFSPVAFSNFT
ETQAEYLIQRLHSRGFEVEKAHAALLLGWQGRPLVSATAWRCGPPP

>PtSCL14

MDPMERAAKVLGSSPGHKNMMGCSSSGVKIEPEIDGLLANAGYTVKASDLAHVAQRLEQLESIMGTVQDPGI
SHLASEAVHYNPSDLAGWIESMFGELNPGADMPVPFGDRGSLIDSSQVHKPIQDDPSLSAMDLALIHEYGLQ
FNGSQASNPQGFSPDSDPSVRCNIFSGPPLRSGDSTTHTNFQARSFSAQSSDEGSSLSSTRLGTAQQSIDNG
AQESGIRVVHLLMGCAEAIQRNNLKVASDLVREIRMTVNSAPCGAMGKVASHFVEALARRICGLNGAESNMS
QADAQSEILYHHFYEVCPYLKFAHFTANQAILEAFEGHGSVHVIDLNLMHGLQWPALIQALALRPGGPPLLR
LTAIGPRQPDGRDVLQEIGMKLAQFAESVNVEFDFRGVMADKLEDIKPWMFQVKPGEVVAVNSVLQLHRLLY
MDAPTGSSPIDVVLKSIGSLRPKIVTVVEHEANHNGPVFLDRFVEALHYYSTMFDSLEACNVLPNSMEKFLA
ELYIQKEICNIVACEGRYRIERHETLSHWRIRLGRAGFRPSHLGSNAFKQARMLLTLFSGEGYTVEENNGSL
TLGWHSRPLIAASAWQGS

>PtSCl16

MEAGMQGASWFSEHQWDHIFDSPPHVDWDSNTTTTQSVAVEQLDVDLDAIDWLSWHDLIIDDINASSVNNMS
LPADLQAMEEYLMDAELQDDNNNMEEALISSPRKDAIVFQSPIDSTDAITLRHPALIAGDQLVSKSIVTGNT
CSYSVDVDHGSVEYKGLRLIHLLTACAEAMLEGAQDLVEVILCRLRQLVSCTGSTMERVGYYLFDALLHSNV
NSLAHLELNDKTTKEHFLGALKLLHAAYPYIRIPHFTANECILEAVVAVGGGNNVGIHIIDFDIMEGMQWPP
LMETLRNKDDVRHLRITTIKWADDEEDEFLRYSGHTGRWLSEYAASAGIPFWYEKMDLKGVKDILPSQEGEI
VVVNCMWQLPHMLNPRNKSHLLEFLVGAHHLNPVAVTLATGPHGTESDSANFVDSLVNRLKELCAIFDSLEA
GLPEHGLARTMVERIFFGPAMSKRVMSSARGDDRPADTTNSNVVDLPLRCGYEECNISNDNMIYAKYNVLMC
SVSGGCYEVELVGEHRLVLKSGCTPLVWVSTWKSPQLSEFDSLNFLEKN

>PtSCL21

MVKDKSNDLGGAQNDKSNDVRICTKGQEQQPWGAQKDKRYVFNGNMEQTNLCLEQISVLACVTGDPMQRLAT
YFMEGLAARITKSWPGLYKALNSTHLQPSMADLVSARQVFFTLFPYLKFAFNTVHLTILESMEKEKVVHIID
LDASEAAQWIPLLQSLSRRPGGPPHLKMTGINEKKEVVQQTAAILTEEAERLDIPFQFNQLVTRLDRLTDDM
LAVKTGEAVAISAVLQFHALLAEDQDFDHNFGGGGGGGGGGGGGGGGGGGRVGINRKRPRQSCTTLGDEDLI
LRSMFNMEALVKKEGSVGRPKYSPSEIEAAISSRRPNLEAAGARSNVEEVRNGRRSTIVEGNIERFLRMLRG
LSPKVMVVVEQESNHNGEHLMDRFVEALYYYSAMFDSLESTLPRQSIERVTVEKYLFGKQIHNIVACEGSER
VERHEKLQGWMKRLDRTGFAQLPLGYTTLLHANNLLHTYTSHSYRLLHHEGCLTICWEERPLYSVSAWQSA
