# Supplementary material for: The GRAS gene family in pine: transcript expression patterns associated with the maturation-related decline of competence to form adventitious roots
Source: BMC Plant Biol. 2014 Dec 30;14:354. doi: 10.1186/s12870-014-0354-8 (PMC4302573; doi:10.1186/s12870-014-0354-8)
Supplement: Additional file 5: — Prediction of intrinsic disorder for the N-terminal region of pine GRAS proteins in each GRAS subfamily. Pine members from each subfamily and representative members from other species were compiled using Clustal. Predicted disordered domains are outlined. Conserved amino acids are displayed in dark grey. Similar amino acids are displayed in light grey. [file 12870_2014_354_MOESM5_ESM.pdf]

SCR

```
PrSCR    MAACIAMNTRFRGGDASGEEVFVVGETDSNNSMIIPASEGENRRKRTGMELNGPLFRRIN 60
AtSCR    MA---------------ESGDFNGGQPPPHSPLRTTSSGSSSSNNRGPPPPPPPPLVMVR 45
ZmSCR    MP------------------------PPPPPPPLTPYCRRCPPPHLPPPPPSSPNHFLLH 36

PrSCR    PSESSDYGQIKWNNLSSGLRESQSPVPLASAAGWQYPSSATVDSVSHRISAPSRTPGLGL 120
AtSCR    --------KRLASEMSSNPDYNNSSRPPRRVS--------HLLDSNYNTVTPQQPPSLTA 89
ZmSCR    YLHQLDHQEAAAAAMVRKRPASDMDLPPPRRH--------VTGDLSDVTAAAAAGVGGSG 88

PrSCR    ANSVLSGESNGQPFNGAYSNMPFFQNSNMERQVSDSCSQWNKWGSVVQPEVGLGCDGTGQ 180
AtSCR    AATVSSQPNPPLSVC-GFSGLPVFPSDRGGRNVMMSV----------------------- 125
ZmSCR    APSSASAQLPALPTQ-LHQLPPAFQHHAPEVDVPAHP----------------------- 124

PrSCR    QLSSLNAVQTMDEAAAAVWVDGMIKDLMMQSS-PNVSMAQIIQSLQEIAL-YNPHIASVL 238
AtSCR    QPMDQDSSS--SSASPTVWVDAIIRDLIHSS--TSVSIPQLIQNVRDIIFPCNPNLGALL 181
ZmSCR    APAAHAQAGGEATASTTAWVDGIIRDIIGSSGGAAVSITQLIHNVREIIHPCNPGLASLL 184

PrSCR    EYRLRSLGQSELGLAGNTQIGRDQNVGMNVSSDGRGFKRSMDQSDHHRRDMNSQQQIMVD 298
AtSCR    EYRLRSLMLLDPSSSS-----------------------------------DPSPQTFEP 206
ZmSCR    ELRLRSLLAADPAPLP-----------------------------------PPPQPQQHA 209

PrSCR    WKSIGANPDSARRSARASQLDDDENRLIDNIHGRGLDVDDQRLRRGNDDLRLRIQSTESH 358
AtSCR    LYQISNNPSPP----QQQQQHQQQ------------------------------------ 226
ZmSCR    LLHGAPAAAPAGLTLPPPPPLPDK------------------------------------ 233

PrSCR    DIQREHQPDSALKLYLDSSGYDQTSWASSKDQFSETAFMANQSMWVQPRQPDNNSQPPVL 418
AtSCR    --QQQHKP-----------------------------------------------PPPPI 237
ZmSCR    --RRHEHP-----------------------------------------------PPCQQ 244

PrSCR    SLQHQANEDDPLRVAPSNDNYITMNRRINTNSNQDPSVNQTPQQQGPSDITASDEEGLHL 478
AtSCR    QQQERENSSTDAP--PQPETVTATVPAVQTNTAEALRERKEEIKR-----QKQDEEGLHL 290
ZmSCR    QQQEEPHPAPQSPKAPTAEETAAAAAAAQAAAAAAAKERKEEQRR-----KQRDEEGLHL 299
                                                                 _______
                                                                    GRAS
```

SHR

```
PrSHR    ----MDRLFTSRLADYQS-------------EHPLSCFNSNKNCEDADTPRHIDSYNNHL 43
PtSCL6   MKGELEIMGINHQADFQISRHAPVLFSEKRMKLEESGQYLVRNEAAALSPQDCFEATGGQ 60
AtSHR    ----MDTLF--RLVSLQ--------------QQQQSDSIITN--QSSLSRTSTTTTGSPQ 38
OsSHR1   ----MDTLF--RLVSLQAA-------SEQQQQQQQSASYNSRSTTSSGSRSSSHQTNASY 47

PrSHR    SQFVLP--------------------------SRRDKSRQCNS------FMEDEDFSFKQ 71
PtSCL6   IGWILSESSELGQPSSVGLTAVTCQESNIDVNSIKARQQSLQFAD----TSDRPDCTRQV 116
AtSHR    TAYHYN-------------------------FPQNDVVEECFN-----FFMDEEDLSSSS 68
OsSHR1   SYYHHSSNSGGGGGGGGGYYYGGQQPPPSQYYYLEPYQEECGNAPHHQLYMDEDFSSSSS 107

PrSHR    FLPFKEMFNSNQTG-KGTTGPNQFSGTRATSSRSSEVP---------------------- 108
PtSCL6   QCIIKNSPKLKSLDSKLAFTPQLRSPSMAATHREWNASTDLNTNRSHYYEELSTAPADQD 176
AtSHR    SHHNHHNHNNPNTYYSPFTTPTQYHPATSSTPSSTAA--------AAALASPYSSSGHHN 120
OsSHR1   SRHFHHGARVQQQQPPASSTPTGTAPTPPLSTSSTAAGAGHGLFEAADLSFPPDLNLDFS 167

PrSHR    DPSLFSELNPSF-PEEFGPAGSRRWASNLLLECARAIAENEKSRTQHLLWMLNELSSPYG 167
PtSCL6   QSWPLLGVSIAKGLGNLGACHDASCMEQLLLHCSSALENNDVTLAQQIMWVLNNIASAEG 236
AtSHR    DPSAFSIPQTPP-SFDFSAN--AKWADSVLLEAARAFSDKDTARAQQILWTLNELSSPYG 177
OsSHR1   SPASSSGGGTAS-SGAVGGGGGGRWASQLLLECARSVAARDSQRVQQLMWMLNELASPYG 226
                                                                 GRAS
```

LlSCL

```
PrSCL1   M--ANEKNGTFDGSKSNGHSNKIGLQRSWLRRETAIPAAQFQGSLSSQKTNGLSNNIGLQ 58
LlSCL    MVKELKVDDFFTDWDAKNG----------FELGWPQPGIDHPTNFG-----DSPNGS--- 42
AtSCL14  M--G-SYPDGFPGSMDELD----------FNKDFDLPPSSNQTL-G--LANGFYLDD--- 41
CsSCL    M--SPNSEDQFSNLIDEYT----------FNQLSPLPTTDLNFLEGPNLLSDVDLGP--- 45

PrSCL1   WSWLQHEAAMPAAQLQDSLSSEKMNGLSDNIGSQRSWSQQEEAVPAAQLLPAAQYQGSLS 118
LlSCL    ---AHVSPNGSALVSPDASATSSNG---------------------------------NV 66
AtSCL14  ---LDFS-SLDPPEAYPSQNNNNNN---------------------------------IN 64
CsSCL    ---TSFEPSITPTDGPEGQPYDPPH---------------------------------VV 69

PrSCL1   SEQTITTTDGDSDDKETYSDIVLKYISDMLMDENMEDKKCMYQECSALQATVKPFYDILG 178
LlSCL    SSSTSFEGDDVYADSEIFSDIVLSYINRMLNEEIIDEKLDIFQGHPELEATEKPFYEILG 126
AtSCL14  NKAVAGDLLSSSSDDADFSDSVLKYISQVLMEEDMEEKPCMFHDALALQAAEKSLYEALG 124
CsSCL    MPPTLQADSTTSPDDNDFSETVFKYMNQILMEENIENKPCMFYDPLGLKDTEKSFYDALG 129

PrSCL1   ENYPPQGMCSKSKIGQNQNQLLNYTKDDVPYKIEDSSWIGEFFNSQ-----MPTGFESPL 233
LlSCL    EKYPPP---SDQPSMYN-NPSPETPDSNIYVKSSSSNSINSVVTSG---NWAFGAIELPQ 179
AtSCL14  EKYPSS---SSASSVDHPERLASDSPDGSCSGGAFSDYASTTTTTSSDSHWSVDGLENRP 181
CsSCL    QKNPPL---PNQNSLESPGLSSSSGDNGTNSTSSSSTVIDALLLSDLGEHNYQPSLS-PP 185

PrSCL1   SANLSAGYSSQYSFSSSGSST-VIDGLPDSPIYEIN-------LSEYFSENKQDSGLG-E 284
LlSCL    NYLIPVDYSSQSSFGSTNSVHNAIEGLGEPTMTN-------IEARDQFSESMLAEQFMRG 232
AtSCL14  SWLH-TPMPSNFVFQSTSRSNSVTGGGGGGNSAVYGSGFGDDLVSNMFKDDELAMQFKKG 240
CsSCL    PILG-EYNAVQSNFNPTDQSQ-FFGNLSDDSVNHFGDGL-AVMAQNIFTNSESILQFRRG 242

PrSCL1   FEEASQVVPKLN-MGISAENGRVMRAPKKETDLELKVGKREDAIGVPFQMNATHSDDENG 343
LlSCL    VEEARKFLPKEDKLVINLEDNGISLPPKLMYDN---GLNEVKEEEKEYTAYGSR------ 283
AtSCL14  VEEASKFLPKSSQLFIDVDSYIPMNSGSKENGSEVFVKTEKKDETEHHHHHSYAPPPNRL 300
CsSCL    LEEASKFLPKSNNLVIDLESNTVSSSEWKKGDDLMVESLVVKGEKESDSSDGSR------ 296

PrSCL1   PKRQKDPHREDLDLEDRQSNKHSAVYSDNVIR---TESFDEVLLCGGRNGKNFADMQVQV 400
LlSCL    -GRKNRHS-DELDLEEGRSNKQSAVDYTEET--LRSEMSDLVLLCPNCDGKEGVSSKTWT 339
AtSCL14  TGKKSHWRDEDEDFVEERSNKQSAVYVEESE---LSEMFDKILVCGPGKPVCILNQNFPT 357
CsSCL    -GRKNHER-EEIELEEGRSNKQTAVYVEESEETELSEMLHKVLLCTDAPPSLYGDNCEPL 354

PrSCL1   LQNGVQKSVQN-GSVKGSQG--PKTVGKKQGKKEVVDLRSLLLICAQSVAADDTRGANET 457
LlSCL    QNEATRSPQN--GHTRGSGS--SKSRGKKPSKTEVVDLRTLLIHCAQTVAIDDRRSANDL 395
AtSCL14  ESAKVVTAQSNGAKIRGKKS--TSTSHSNDSKKETADLRTLLVLCAQAVSVDDRRTANEM 415
CsSCL    QNDASKASPQTKGQVQGANGGKGSRNKKQGKKKETVDLRSLLILCAQAVSTGDGRTANEL 414
                                                  ______________________
                                                                    GRAS
```

PAT

```
PrSCL10   MSLASPCQESMRGDDNSYRTNSWYRGNLSGVPEISRNMGMQYMPGGIGLNLQRGWGNSFR  60
AtSCL5    MRLSVFIIPLVESRQASGIINKQST--------SLLIRFSLYLEASISTKSFFSKSQRIS  52
OsCIGR2   ----------MADTPTSRMIHPFSN--------IPSQNLKQFQYSDNPQHPCHP-----Y  37
AtPAT1    ------------------------------------------------------------

PrSCL10   SCSTDLSPKQNGNFAIDHTSTTGERSAGTLKMIDQRSPISPLSPKGIGSDGLPQTSQSQR 120
AtSCL5    QTQSPICLSAN-----YYQPDNLDMEATQKHMIQEGSSMFYHQPSSVKQMDLSVQTFDSY 107
OsCIGR2   RAPSDTHVVPH-----HYGLKSHSPDAG------------YESQATPNKYTLD-------  73
AtPAT1    ----------------MYKQPRQELEA------------YYFEPNSVEKLRY--------  24

PrSCL10   LYSSERIQMHDDLIHSSSHNHGSFNDQVYIHESFPCNNELSRESSICIQSSDGSPLSSQH 180
AtSCL5    CTLESSSGTKSHPCLNNKNNSSST-------TSFSS--------------NESPISQAN 145
OsCIGR2   ----------SSEGAGCMRHDSPSS-------QSFTTR-------------SGSPLS--- 100
AtPAT1    -----------LPVNNSRKRFCTL-------EPFP--------------DSPP----  45

PrSCL10   SQSYSSDSRYFHSAEDAADLVGNAYSGNGQPANLKILEEIETALLGPDGDDPLDMDYAYG 240
AtSCL5    NNNLSRFNNHSPEENNNSPLSGSSATNTNETELSLMLKDLETAMMEPD------------ 193
OsCIGR2   ------QEDSHSDSTDGSPVGASCVTEDPN-DLKQKLKDLEAVMLGPD------------ 141
AtPAT1    ------YNALSTATYDDT--CGSCVTDELN-DFKHKIREIETVMMGPDS-----------  85

PrSCL10   DHTLNSNSEWSLEALSDFNDFLDQTSNPGYSQINNADSSRVPDNLQQLTNHGDRNVKEET 300
AtSCL5    -----------VDNSYNNQGGFGQQHG--------------------------------- 209
OsCIGR2   -----------SEIVNSLENSVANQLS--------------------------------- 157
AtPAT1    -----------LDLLVDCTDSFDSTAS--------------------------------- 101

PrSCL10   VNEVNLKIEHFSPSSSDMEKLEIEELGSPQGDVKSLLIECAKAIADGR--NADNLIAGLR 358
AtSCL5    ---------------VVSSAMYRSMEMISRGDLKGVLYECAKAVENYDLEMTDWLISQLQ 254
OsCIGR2   ---------------LEPEKWVR-MMGIPRGNLKELLIACARAVEEKNSFAIDMMIPELR 201
AtPAT1    ---------------QEINGWRSTLEAISRRDLRADLVSCAKAMSENDLMMAHSMMEKLR 146
```

GRAS

AtSCL3

```
PtSCL21  MVKDKSNDLGGAQNDKSNDVRICTKGQEQ-------QPWGAQKDKR-------------- 39
AtSCL3   MVAMFQEDNGTSSVASSPLQVFSTMSLNRPTLLASSSPFHCLKDLKPEERGLYLIHLLLT 60

PtSCL21  ---YVFNGNMEQTNLCLEQISVLACVTGDPMQRLATYFMEGLAARITKSWPGLYKALNST 96
AtSCL3   CANHVASGSLQNANAALEQLSHLASPDGDTMQRIAAYFTEALANRILKSWPGLYKALNAT 120

PtSCL21  HLQP-SMADLVSARQVFFTLFPYLKFAFNTVHLTILESMEKEKVVHIIDLDASEAAQWIP 155
AtSCL3   QTRTNNVSEEIHVRRLFFEMFPILKVSYLLTNRAILEAMEGEKMVHVIDLDASEPAQWLA 180

PtSCL21  LLQSLSRRPGGPPHLKMTGINEKKEVVQQTAAILTEEAERLDIPFQFNQLVTRLDRLTDD 215
AtSCL3   LLQAFNSRPEGPPHLRITGVHHQKEVLEQMAHRLIEEAEKLDIPFQFNPVVSRLDCLNVE 240

PtSCL21  MLAVKTGEAVAISAVLQFHALLAEDQDFDHNFGGGGGGGGGGGGGGGGGGGRVGINRKRP 275
AtSCL3   QLRVKTGEALAVSSVLQLHTFLASDDDLMR------------------------KNCALR 276

PtSCL21  RQSCTTLGDEDLILRSMFNMEALVKKEGSVGRPKYSPSEIEAAISSRRPNLEAAGARSNV 335
AtSCL3   FQNNPSGVDLQRVLMMSHGSAAEARENDMSNNNGYSPS---------------GDSASSL 321

PtSCL21  EEVRNGRRSTIVEGNIERFLRMLRGLSPKVMVVVEQESNHNGEHLMDRFVEALYYYSAMF 395
AtSCL3   PLPSSGR--------TDSFLNAIWGLSPKVMVVTEQDSDHNGSTLMERLLESLYTYAALF 373

PtSCL21  DSLESTLPRQSIERVTVEKYLFGKQIHNIVACEGSERVERHEKLQGWMKRLDRTGFAQLP 455
AtSCL3   DCLETKVPRTSQDRIKVEKMLFGEEIKNIISCEGFERRERHEKLEKWSQRIDLAGFGNVP 433

PtSCL21  LGYTTLLHANNLLHTYTSHSYRLLHHEGCLTICWEERPLYSVSAWQSA- 503
AtSCL3   LSYYAMLQARRLLQGCGFDGYRIKEESGCAVICWQDRPLYSVSAWRCRK 482

                                                                GRAS
```

DELLA

```
PtSCL14   MDPMERAAKVLGSSP---------------------------------------------  15
PpSCL15   ---MKRQHFRLQQQQPQPNGHGRCCSTVPVHENPVSMPGSGPPPPSPRTTATAPAAGAA  57
AtGAI     ---MKRDHHHHHQD----------------------------------------------  11
OsGAI     ---MKREYQEAGGSS---------------------------------------------  12

PtSCL14   ------------GHKNMMGCSSSGVKIEPEIDGLLANAGYTVKASDLAHVAQRLEQLESI  63
PpSCL15   AAGGGGGSGSCKGKEVVLKDTS---KQGVGVDMELASMGYSVKSSELEQVAHRLEQLEMM  114
AtGAI     -------------KKTMMMNEE---DDGNGMDELLAVLGYKVRSSEMADVAQKLEQLEVM  55
OsGAI     --GGGSSADMGSCKDKVMAGAA---GEEEDVDELLAALGYKVRSSDMADVAQKLEQLEMA  67

PtSCL14   MGT------VQ--DPGISHLASEAVHYNPSDLAGWIESMFGELNPG--ADMPVPFGDRGS  113
PpSCL15   MCN------GQE-DGIISHLSSEAVHYNPSDLGGWIESMLSELHVPILPPTDQPFQFPQA  167
AtGAI     MSN------VQEDD--LSQLATETVHYNPAELYTWLDSMLTDLNPP--------------  93
OsGAI     MGMGGVSAPGAADDGFVSHLATDTVHYNPSDLSSWVESMLSELNAPLPPIPPAPPAARHA  127

PtSCL14   LIDSSQVHKPIQDDPSLSAMDLALIHEYGLQ-----------------------------  144
PpSCL15   AADQSSTVREASNSVPESSTSTSKGTRSVQNVEQDQQYRLMGPGQGGVRDNGIDRMFSNY  227
AtGAI     ----------------------SSNAEYDLK-----------------------------  102
OsGAI     STSSTVTGGGGSGFFELPAAADSSSSTYALRP----------------------------  159

PtSCL14   ----------FNGSQASNPQGFSPDSDPSVRCNIFSGPPLRSGDSTTHTNFQARSFSAQSS  195
PpSCL15   GGLFSQVLDVSDLLVDDPDVLQEPPPQEASPSTLLLQSSSNSSLEVQSGLDHLEEDVTGR  287
AtGAI     --------AIPGDAILNQFAIDSASSSN--QGGGGDTYTTNKRLKCSNG------VVETT  146
OsGAI     -------ISLPVVATADPSAADSARDTKRMRTGGGSTSSSSSSSSLGGASRGSVVEAA  212

PtSCL14   DEGSSLSSTRLGTAQQSIDN--GAQESGIRVVHLLMGCAEA
PpSCL15   EQKRYRVCDPQLSERTVVVMGADPHESGVRLVHTLMACAEA
AtGAI     -------TATAESTRHVVLV--DSQENGVRLVHALLACAEA
OsGAI     PPATQGAAAANAPAVPVVVV--DTQEAGIRLVHALLACAEA

                                  ______________
                                            GRAS
```

HAM

```
PtSCL12   MNGMLSRTSGNSLPPAQLKHQILQSKT-----------------SPDQAQKRHCKSSVWQ 43
PtSCL13   ---MLDRN--TETRPPLLNLKLVQSSS-----------------IPAQANNKR------P 32
PhHAM     ---MIAIPQGAKGVLGVSSFMPSISSEEQICKKDANFARNDPQICKKDGNFARNEPQICK 57
AtSCL6    ----MPLPFEEFQGKGISCFSSFSSS--------------FPQPPSSPLLSHRKARGGEE 42
AtSCL22   ----MPLPFEQFQGKGVLGFLDSSSSP------------GYKIWANPEKLHGRVEEDLCF 44

PtSCL12   RSRSGGEIEPTSVLDIRSPSPTSTLSSSLGGSSESAGAVAAVSSGLLCEAVSDGGGGGSG 103
PtSCL13   YVRSEYQSEPTSVLDIRSPSPTSTLSVSFGGSTD----------GGFFRENACDG------ 77
PhHAM     KEGNFTRNEPVSVLDTRSPSPSSSSCSYAGKVEG-------------------------- 91
AtSCL6    EEEEVPAAEPTSVLDSLI-SP----TSSSTVSSS-------------------------- 71
AtSCL22   VVNNGGFSEPTSVLDSVRSPSPFVSSSTTTLSSS-------------------------- 78

PtSCL12   SGGAETLLLESSGKEAFNNGGGSTGSPARWYSNRGGSGNGGREGFGWRLNNGEQQPGGGG 163
PtSCL13   ------QLLDCIGVMDSKDGGN------------------------WQLQN--------- 98
PhHAM     ------------------------------------------------------------
AtSCL6    ------------------------------------------------------------
AtSCL22   ------------------------------------------------------------

PtSCL12   MVEGKSEVKKEDPQQHSRAEEWGSGGGAGTAGGMDDLESMLFESGAAPPDQSLMRWLL-G 222
PtSCL13   ------VIRNENEFQVAETEAVNVN--PNVSMGMEYLENILLQS-PEGNNNSHIPCLM-G 148
PhHAM     --------QKEELVKELQPFPLVGLEPEKCSLGFGDLDNLLP-E-LCGADQTLLRWIS-G 140
AtSCL6    --------HGGNSAVGGGGDATT--DEQCGAIGLGDWEEQVP----HDHEQSILGLIM-G 116
AtSCL22   --------HGGPSGGGAAAATFSGADGKCDQMGFEDLDGVLSGG-SPGQEQSIFRLIMAG 129

PtSCL12   EIEDP-KDLPPPQIKSATSG-GSTSAPQFVDPSIEPNFA---DPGAFAFSNNNLSDVIAP 277
PtSCL13   EPCLMGNEYPLQGIKAEEAVLQQTHFNEATDSQLSEFFCGNSDIGPYSFFGDNAGHTISP 208
PhHAM     DMEDPSVSLKQLLLGGNANGFHGSSGFEASAAGSLVHTDNVSFSGSDVSLNANIEKIGSV 200
AtSCL6    DSTDPSLELNSILQT----------------------------SPTFHDSDYSSPGFG-- 146
AtSCL22   DVVDPGSEFVGFDIG----------------------------SGSDPVIDNPNPLFGYG 161

PtSCL12   PQHASVPSSFRPSYATLPNLPQQQFIPPPPPPPPPPPPPFAYNGPMPTPFGPNAYHPEVL 337
PtSCL13   FALNPTSTALFP---------PSNALITPNIHGPNPPPLSFTPD-FKTFKVPLDEKHPELQ 259
PhHAM     IDSNGRPSNGFD------------YLNANLYAKSLPLPPASSFQEQKLGQKPQSFSPNQA 248
AtSCL6    ------------------------VVDTGFGLDHHSVPPSH------------------- 163
AtSCL22   FPFQNAPEEEKF------------QISINPNPGFFSDPPSS------------------- 190

PtSCL12   FSGPAVYGSNPVQHLGPDFPRFNMAADPNRNNNNLLFDMPHPPPPKRFGLEHQLWQQTFK 397
PtSCL13   LP----YGPN------PSYP----------EKNVVFPMPAAPRG-HVGVAHEGFPEFEK 297
PhHAM     LFQNASYGNM------------------FDSSSYDDTNQEQPPSKRHNSGTLGSSVGGL 289
AtSCL6    -------------------------------------VSGLLINQSQTHY----- 176
AtSCL22   -------------------------------------PPAKRLNSGQPGS----- 203

PtSCL12   QQEYMNMMKPQQQQELLQSLHR--RQQFLHQAPPPSPHHQMRQKAVVNHTFKVGG---SG 452
PtSCL13   SLHWQQIQQREFQPAWKTMINRSVRDPLFSAVKQEWPGRTHQGRVDLDEVQKS------- 350
PhHAM     LPKVPFFDPSGDLSMRRQLLGEMQQHFNLLPPQQFQPKPLIVPKLEAACGGNGSLMVPR 349
AtSCL6    --------------------TQNPAAIFY---GHHHHTPPPAKRLNPG------------ 201
AtSCL22   --------------------QHLQWVFPFSDPGHESHDPFLTPPKIAGED---------Q 234

PtSCL12   AVADEVHVIVEQLLKAAEA
PtSCL13   -------AILQQLLNATNY
PhHAM     HQQQEQQFIYDQIFQASEL
AtSCL6    -----PVGITEQLVKAAEV
AtSCL22   NDQDQSAVIIDQLFSAAAE
                 ___________
                    GRAS
```

LsLS

```
PrSCL2    MSMGGVNMNMNMSPWLSAPTAWQHDGLMSFPPGICTDVVGDLCSTTTNSYLSEPNSVLDL 60
LsLS      ------------------------------------------------------------
AtSCL4    MAYMCTDSGNLMAIAQQVIKQKQQQEQQQQQHHQDHQIFG----INPLSLNPWPNTSLGF 56
OsMOCI    ------------------------------------------------------------

PrSCL2    SRASMLFSPMEQFQDFTQHLHDGIGAKQMPLLGGVEPPAPAFKDNALYHHEALVKKERPP 120
LsLS      ------------------------------------------------------------
AtSCL4    GLSGSAFP------------------DPFQVTGGGDSNDPGFPFPNLDHHHATT------ 92
OsMOCI    ------------------------------------------------------------

PrSCL2    IKEPGVGDGSSAALRSSVFRSPELHTFGFESPVSNRNDFVSPDVCTEWMDCLMSDLQPEA 180
LsLS      ------------------------------------------------------------
AtSCL4    -----TGGG---------FR---LSDFGGG---TGGGEFES----DEWMETLIS------ 122
OsMOCI    ------------------------------------------------------------

PrSCL2    IAGNGNAAIASCFDSSNPCESTESSELPNGGSGYRRHSSEDAAAWQSEFGILGEAFKRSS 240
LsLS      ------------------------------------------------------------
AtSCL4    ----GGDSVADGPD---CDTWHDN----------------------PDYVIYGPDPFDTY 153
OsMOCI    ------------------------------------------------------------

PrSCL2    PTSWTPDTTTEQLLCSSPSADLLSAASSELLSTNSLKGKAALQEQYGGAAGGGHEFQTLH 300
LsLS      ------------------------------------------------------------
AtSCL4    PS------------------RLSVQPSDLN--------------------------RVID 169
OsMOCI    ------------------------------------------------------------

PrSCL2    HRMPNVHQSVCPPKQQLIREPSTPPQKRQQGQQEQDPLKGKREEDPPKPEIDALDRHMQG 360
LsLS      -MLGSFGSSSSQSHPHHDEESSDHHQQRRFTATATTIT-TTTITTSPAIQ---------- 48
AtSCL4    TSSPLPPPTLWPPSSPLSIPPLTHESPTKEDPETNDSEDDDFDLEPP------------- 216
OsMOCI    -MLRSLHSSSSSDTDNNSGGCKNNGGGGGEAAAAVEGGGDQRAVAAAAPS---------- 49

PrSCL2    QRSDLLHSLLDCAKIVDTEPERAGQS--VAYLQRIASHHGDPTQRIASHFADALAKRLSK 418
LsLS      ----IRQLLISCAELISQSDFSAAKR-LLTILSTNSSPFGDSTERLVHQFTRALSLRLNR 103
AtSCL4    ----LLKAIYDCARISDSDPNEASKT--LLQIRESVSELGDPTERVAFYFTEALSNRLS- 269
OsMOCI    ----TRDLLLACADLLQRGDLPAARRAAEIVLAAAASPRGDAADRLAYHFARALALRVD- 104
              ________________________________________________________
                                                                  GRAS
```

AtSCL26

```
AtSCL26   ------------------------------MNYPYEDFLDLFFSTHTDP----------- 19
PtSCL11   MVSRLQSPPSCCKATMSTISYNSHAPSFQFYQNPSSDWLLHNKAMDIQSHHQHQQFLFWE 60
PtSCl16   ---------MEAGMQGASWFSEH-QWDHIFDSPPHVDWDSNTTTT--------------- 35
PtSCL19   ---------METGMAGTPWLSEQ-VWDHIFYNPPHADWYGNTNTHSAA------------ 38
PtSCL22   ------MGSMEDGT-GTSWFSEQFDWDHVFTSLPQVDWDTNSA----------------- 36
PtSCL23   -------MQETGMGSYNSWFSEQ-EWDHVFSNNPPDGGITYDVAE--------------- 37
PtSCL24   --MWNMGSSMEAAT-GASWFSEH-QWDHVFDNPPHVDWDTNTTTT--------------- 41
PtSCL25   -------MGGVEDGTGTTLFSEK-DW-HY--SLLDVDWDTNSP----------------- 32
PtSCL26   ---MSSVSSMEAGS-GTAWFSEL-ELDHLFTSLPHEDWDSNSA----------------- 38
PtSCL27   --------------MAASWFCEQ-EWDHIFNNLPPVDSDTDTNYT--------------- 30
PtSCL28   ---------MEATMAAAAWFCEQ-EWDHIFNNLPPVDWDTDTNNT--------------- 35
PtSCL29   ---------MEAAT-GASWFSEE-DLDHIFNTLPRVDWHTNSPST--------------- 34
PtSCL30   ---------MEAGM-GASWLSEY-HWDHLFDNPP----HVDTPST--------------- 30

AtSCL26   ------------LATAASTSSNGYSLNDLD-IDW-------------------------- 35
PtSCL11   PTPLLPANHKLNPFTAFPPTSLESIELSKSDADYTSLWESRQIDQKQHISALILRASERD 120
PtSCl16   -----------------QSVAVEQLDVDLDAIDWLS-WHDLIIDD--------INASSVN 69
PtSCL19   -----------------AAAAEEQMDVDLDAAECPS-WGDLIINK--------SNGCTVN 72
PtSCL22   --------------------AVEQLDVDLDTAQWSP-WDDLLVDD--------NH----- 62
PtSCL23   --------------------GEQLMDVDLDAPEWSS-WDDLIIDVD-------INGCSGD 69
PtSCL24   -----------------QSTAV---DVDLDAVDWSS-WDNLIIDH--------VNGCSVN 72
PtSCL25   --------------------AVELPDVHLDAAQWSS-LDDLLVDD--------NHGCSFN 63
PtSCL26   --------------------ALGHLHMDLDGAQWSS-WDDLIIPD--------NNGCSVN 69
PtSCL27   -----------------PSTPVQQLGADLDAVDWLS-WDDLIIN-----------GCSLN 61
PtSCL28   -----------------PSTPVQQLGADLDAAEWSS-WHDLIID-----------DCSLN 66
PtSCL29   -----------------AVEELM--DVELDTAEWSS-WDDIIIHD--------NNGGSFS 66
PtSCL30   -----------------ALQQL---EVVFDAAEWAS-WDDLFIN-----------GCSVN 58

AtSCL26   -----DCDFRDVIESIMGDEGAMMEPE--------------SEAVPMLHDQ------EGL 75
PtSCL11   YLENKVDFCDNVSVSTVTDEKYVLDGLYQGFDEEGGIVPSKAFGELPFPFFDDGIDVISF 180
PtSCl16   N--------MSLPADLQAMEEYLMDAELQDDNNN------MEEALISSPRKDAI-VFQSP 114
PtSCL19   ND-------QTLPADLQAIREYLLDEEVQDYDSN------IQED--PSPGRDASDPVKNS 117
PtSCL22   ----------SLPTELQVIGEHVIDIDLQEDDNK------IEEYSISSRRRDAN-VFQCP 105
PtSCL23   TSNSNPSPNPSLPANLQAIEEHLMDVELQDVNNN------FQEDAVSCPRIDDAIVANSS 123
PtSCL24   N--------LSLPADLPEMGAHLMDAELQNDNNN------SEEDLISSPRKDAI-FFRRP 117
PtSCL25   N--------LLLSTELEAAGEYLIDADRRDDNNN------IEEDPIWRPQIDPI-FFQCP 108
PtSCL26   K--------LSLPTDLLAIEKYLKYTDVQDDNIN------IEEDPISSPRSDAN-VLQIP 114
PtSCL27   N--------VSLPADLQTIEEYLMDEGLQDDSND------MEQDLISSPTNDAMTVFQCP 107
PtSCL28   N--------LSLPADLQTIEEYLMDEELQDDSND------MEQDLISSPTNDAMTVFQCP 112
PtSCL29   N--------LPLPADLKTIEEYLMDGELHDGYNN------EEDNLILSPPKDAI-VFQCP 111
PtSCL30   N--------LSG--------EYLMDAELQDESNN------IEEDHILSHRKDTI-IFQHT 95

AtSCL26   CNSASTGLSVADG-----------------------------------VSFGEPKTDESK 100
PtSCL11   LGNTQNPCSPSDHSEAVGSIGSCSSASLNSIDKFGNEFVLRDEYPSEFEAVLGLQVPLLG 240
PtSCl16   IDSTDAITLRHPALIAGDQLVS------------KSIVTGNTCSYSVDVDHGS---VEYK 159
PtSCL19   GPKAGDQLLSDTGLVTNSSSESKSNETEKLG--SGSGSGSGSGSSSVEVDDGALKDSEYK 175
PtSCL22   INNEY-PAALIAA----NQPLS------------------YSGPPSVDVDHGALEDMEYK 142
PtSCL23   CKSIETDNAGSSS---VDIDHAG----------------SHENRASKDLKTIS--EVEYK 162
PtSCL24   IDSTDPVTLQHPALIAGDQLVS------------KSTVTDNTGSSSVDVDHGS---VEYK 162
PtSCL25   VDTELHPAALIAA---DRSLSD------------FAVVTNKSVSPSVLIDHGAFEETEYK 153
PtSCL26   IDNEHPAEDQLFS----GSAHV---------------VTNKGDTSSLNIDHGSLQDMECN 155
PtSCL27   IDSSADLVTPEHP--AGDQLVQT----NSSSSESKSMERHNAGSSSVDVSDSS---LEYK 158
PtSCL28   IDSSADPVTLEHP--AGDQLVQT----NSSSSESKSMVTDNPGSSSVDVSDGS---LEYK 163
PtSCL29   VDSTDPVNFDQPTLITGDQLVSDYAVTNSSS-ELKSIVTENAGLSSVDVDHGS---VEYN 167
PtSCL30   IDSADTITSEQSGVIPRDQLVSDYTATDSS--RSESIAPNKADSSSVDVDHGS---VEYK 150
```

GRAS
